# An agent-based model of *Trypanosoma brucei* social motility to explore determinants of colony pattern formation

**DOI:** 10.1371/journal.pcbi.1013698

**Published:** 2026-08-12

**Authors:** Andreas Kuhn, Timothy Krüger, Markus Engstler, Sabine C. Fischer

**Affiliations:** 1 Chair for Computational and Theoretical Biology (CCTB), Biocenter, Julius-Maximilians-Universität Würzburg, Würzburg, Germany; 2 Department of Cell and Developmental Biology, Biocenter, Julius-Maximilians-Universität Würzburg, Würzburg, Germany; CSIC: Consejo Superior de Investigaciones Cientificas, SPAIN

## Abstract

*In vitro* colonies of the unicellular parasite *Trypanosoma brucei* expand radially and establish fingering instabilities, a collective behavior known as social motility. The underlying mechanisms are thought to involve single-cell motility, chemical communication among cells, and mechanical interactions with the liquid boundary, but their relative contributions remain unclear. We aimed to determine which of the mechanisms are necessary to quantitatively reproduce the morphological characteristics of social motility. We developed a two-dimensional agent-based model that simulates colonies of $10^{5}-10^{6}$ cells at single-cell resolution—two to four orders of magnitude larger than previous models. Cells are represented as point particles executing directional random walks with auto-chemotactic alignment and exponential colony growth. The colony boundary is modelled using a grid-based approach in which interactions with agents can locally weaken and expand it. The model was quantitatively evaluated by applying our previously established morphology metrics. We show quantitative agreement of the simulation results and experimental data in terms of colony morphology. Parameter exploration revealed that finger formation arises within a narrow range of trypanosome motility parameters that balance stochasticity and alignment, while boundary conditions modulate the speed of colony expansion. The diffusion coefficient of the chemotactic signal is the key determinant of pattern formation. Realistic behavior occurs at $2\times 10^{-11}$ – 10^-10^ m^2^/s which corresponds to molecules of 12.1–1690 kDa. These results demonstrate that complex colony morphologies can emerge from minimal cell-level rules, suggesting testable hypotheses for the molecular drivers of trypanosome social motility. Furthermore, our approach provides a framework for dissecting the interplay between motility, signaling, and mechanical confinement in other microbial systems exhibiting collective behavior.

## Author summary

*In vitro* colonies of the unicellular parasite *Trypanosoma brucei* expand radially and form finger-like patterns, a behavior known as social motility. The ability of trypanosomes to exhibit social motility has been linked to their successful journey through their insect vector, the tsetse fly, which facilitates their survival and spreading between hosts. However, the mechanisms driving this behavior are not yet clear. Experimental data suggest that the movement and growth of individual cells, their interactions with each other, and their effects on colony boundaries may all play a role. These different factors are difficult to separate in experiments. We employed mathematical modeling to investigate the relative importance of these factors and developed a model that represents individual cells. The simulations reproduced finger formation patterns similar to those observed in experiments. The results show that complex colony shapes can emerge from simple cell behaviors. We found that the speed at which a signaling chemical spreads between cells is crucial, and that the predicted values do not match the properties of the chemicals proposed so far. Identifying and testing candidate signaling molecules in experiments could be the next step. Additionally, our approach may also aid in understanding collective behaviors in other microorganisms.

## Introduction

**Fig 1:**
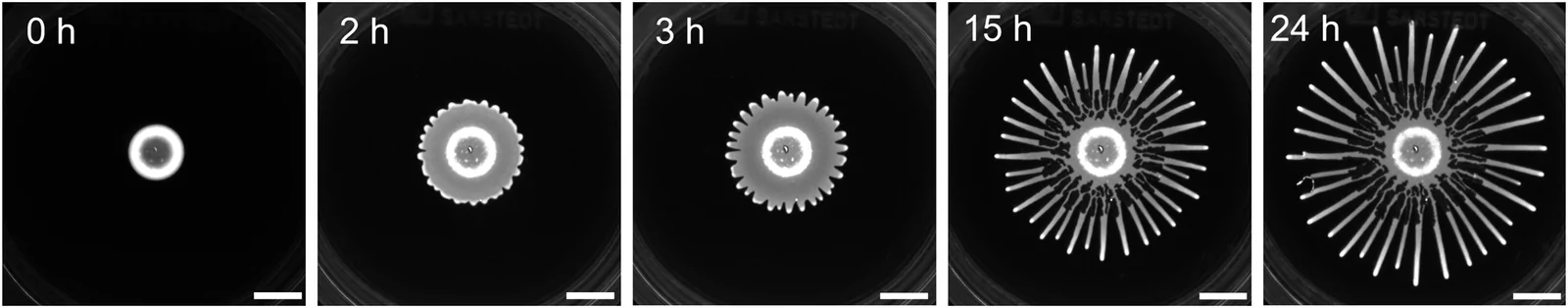
Time series of a *Trypanosoma brucei* colony ($2\times 10^{6}$ cells) exhibiting social motility. Fluorescently labelled cells initially expand radially but form finger-like structures after 2 h at colony boundaries. Scale bar: 5 mm. See see section A.3 in S1 Appendix for methodological details.

Understanding how individual behaviour gives rise to complex collective phenomena is a fundamental challenge across biology. Social motility [1–3] in *Trypanosoma brucei* presents a striking example: densely packed cells swimming in an apparently random manner [4] on agarose gel surfaces form intricate colony-level patterns. These colonies initially exhibit radial outgrowth before entering a second phase characterised by fingering instabilities [5], with fingers growing in perpendicular alignment away from the colony centre (Fig 1).

Although the molecular and cellular mechanisms underlying individual trypanosome motility have been extensively studied [6–10], their collective behaviour remains poorly understood. These finger patterns superficially resemble a microbial colony exhibiting diffusion-limited growth, yet the underlying mechanisms differ fundamentally. In diffusion-limited growth, largely sessile cells proliferate preferentially at colony boundaries where nutrient availability exceeds that in the colony interior [11–13]. Trypanosome colonies, in contrast, are dynamic entities with continuously moving cells that pause only briefly at boundaries [4]. Crucially, nutrients are not limiting in these systems, and cells divide at rates independent of their position within the colony.

Despite visual and conceptual similarities between *T. brucei* colonies and bacterial swarming systems [14–20], it remains unclear to what extent insights from bacterial motility can be transferred. Such bacterial systems have been modelled using continuum approaches, in which population-level behaviour is described through density fields governed by reaction–diffusion equations [16,17,21,22] or by individual-based models [18,23].

*Pseudomonas aeruginosa* stands out as the system most closely resembling *T. brucei* in both colony morphology with similar finger instabilities [24] and as well in microswimmer dynamics with similar swimming modes [25,26] as *Trypanosoma brucei* [27,28]. However, a mechanistic understanding of *P. aeruginosa* swarming remains incomplete and is complicated by system-specific features, such as the secretion of rhamnolipids, which change the physical properties of the colony boundary. This limits the direct transferability of concepts to *T. brucei*. Swarming *Proteus mirabilis* colonies represent another biological system that exhibits notable morphological similarities to *T. brucei*. These flagellated microswimmers produce complex spatial patterns, including circular, terraced, and chiral colony structures, mediated by collective swarming and chemotactic responses [29,30]. However, a fundamental mechanistic difference exists: while the finger-like protrusions in *T. brucei* facilitate outward radial expansion, the radial density fluctuations in *P. mirabilis* are directed inwards and emerge from the interplay between *swimmer* cells that migrate along self-produced attractants towards the colony center, and *swarmer* cells that dominate the leading edge to facilitate outward circular colony expansion [31].

One prominent hypothesis is that social motility results from chemotactic responses at the colony level [32–34], with cells reacting to environmental cues such as exosomes [32], neighbouring *E. coli* colonies [33], or self-generated pH gradients resulting from glucose metabolism [34,35]. Alternative hypotheses suggest that physical interactions, including hydrodynamic coupling, steric alignment between adjacent cells, or interactions with the liquid–solid boundary of the colony, contribute to the emergent patterns observed during collective movement [4]. The relative contributions of these chemical and physical mechanisms are unclear. Addressing this question requires an individual-based model.

Here, we present a two-dimensional agent-based model that simulates individual trypanosomes at populations of $\sim 10^{5}$ to 10^6^ cells, consistent with social motility assays [1,2,4,5,32]. This represents an increase of four orders of magnitude in agent numbers compared to existing models of trypanosome [27,28,36], which examine the swimming dynamics of individual cells, and an increase of two orders of magnitude compared to other microswimmer models [37–40] for collective behaviour.

We employ several key assumptions to make such large-scale simulations computationally feasible over the social motility timescales of up to 3 days. First, we represent each trypanosome as a point particle on a square lattice, with spacing matched to the projected area of real trypanosomes. The complex swimming motion of individual trypanosomes is reduced to a dry active matter model [41] where agents perform a directional random walk with reflective boundary conditions at the colony boundaries, capturing the essential persistent motion of these microorganisms in confined spaces [33] while abstracting away their detailed swimming dynamics [42,43].

Second, we model the complex liquid boundary of the colony on top of the semi-liquid agarose matrix by dividing the simulation space into walkable and non-walkable lattice sites, with the initial spherical colony region designated as walkable. Collisions of the agents with the non-walkable lattice sites on the colony boundary make those sites progressively walkable and part of the colony. Such an approach has been successfully used before to model the growth of bacterial colonies [22,44].

Third, we model the proposed chemical signalling and diffusion that mediates cell-cell communication by a reaction-diffusion equation and negative autochemotaxis similar to previous models for bacteria chemotaxis [16,21,44–47]. For this, we employ a separate, coarser grid. Our dual-grid approach balances biological realism with computational efficiency, allowing us to capture diffusion-mediated interactions while maintaining tractable simulation times.

Through analysis and comparison with experimental observations using our previously established metrics to quantify *Trypanosoma brucei* colonies [5], we demonstrate that these simplified rules enable the reproduction of the morphological characteristics of social motility. We further analyse how the individual components change the emerging behaviour and found that they exhibit distinct effects. The colony morphology is very sensitive to the motility parameters of the single cells. The parameters for boundary interactions mostly affect expansion speed. The diffusion coefficient of the chemotactic signal affects both expansion speed and colony morphology. Hence, it is a key parameter for obtaining finger-like patterns comparable to experiments. We could not identify the chemical component that most likely drives social motility because the optimal diffusion parameter value does not match any of the previously suggested chemical components.

## Materials and methods

### Model architecture

**Fig 2:**
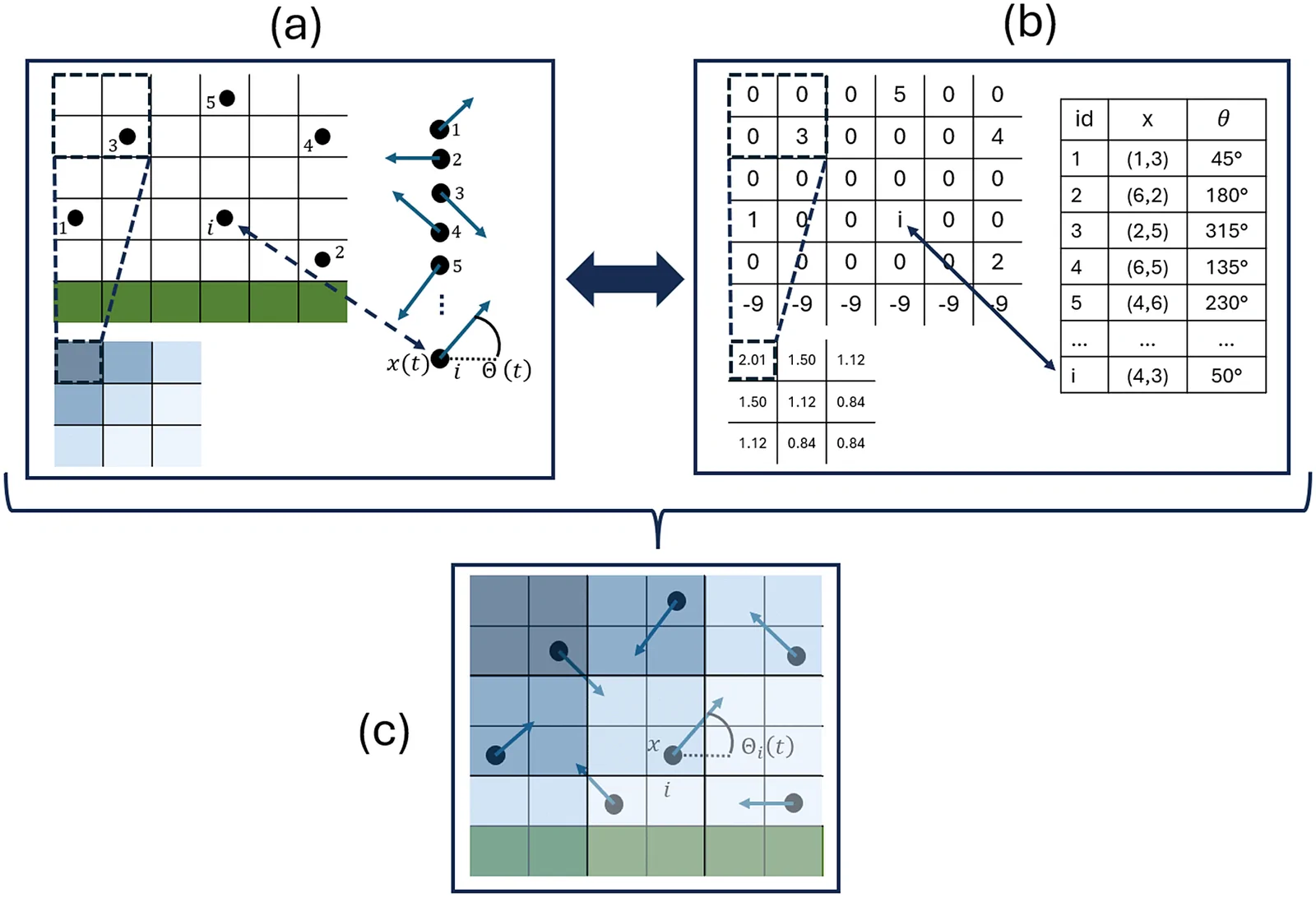
Schematic of the model architecture. (a) Conceptual representation of individual components: ABM grid (left), agent table/list (right), and gradient grid ($s_{f}=2$) (below). Each agent (black dot) occupies one cell in the ABM grid; walkable cells are white, and non-walkable cells are green. The agent table stores the position x(t) and direction θ(t) (blue arrow) of all agents. With scaling factor $s_{f}=2$, four ABM grid cells map to one gradient grid cell, with chemical concentration indicated by blue colour intensity. (b) Computational implementation of the data structures from (a): The ABM grid is a 6 × 6 2D integer array, where negative values indicate the grid strength *k*, zero indicates a free grid cell, and positive values indicate the agent id of the occupying agent. The agent table is a vector containing position (integer tuple) and orientation (floating-point value between 0 and 2π), and the gradient grid is a smaller 3 × 3 2D floating-point array. (c) Conceptual representation of the integrated model: agents move in their current direction through varying chemical concentrations on empty ABM grid cells.

To model trypanosome social motility, we combined an agent-based model for the individual cells with a continuous model for chemical signalling and diffusion. The state of the model is stored in three primary data structures (Fig 2). First, a table of agents representing the trypanosomes contains for each entry *i* a unique identifier (id), the position $x_{i}(t)$, and the orientation $\theta_{i}(t)$ as the angle in the 2D plane relative to the horizontal axis (Fig 2 (a)). Second, a 2D integer array called the agent-based model grid (ABM grid) models the spatial environment. Third, a 2D floating-point array called the gradient grid stores the chemical concentration values s(x). The ABM grid is a square lattice for which the lattice constant Δx was set to 3.75μm, corresponding to a unit cell area of $14.06\;\mu m^{2}$ (see section A.1 in S1 Appendix for more details). Each grid cell stores an integer value *k*, where:

- *k* > 0 indicates the unique id of an agent occupying that cell (only one agent per grid cell is possible),
- *k* = 0 represents an empty, walkable grid cell,
- *k* < 0 denotes a non-walkable grid cell outside the colony, with the absolute value of *k* determining the grid strength.

The gradient grid stores the local chemical concentration at each grid point and handles calculations for chemical diffusion, adsorption, and decay. To reduce computational cost, this grid can operate at a coarser resolution than the ABM grid, controlled by an integer scaling factor $s_{f}$ (e.g., 1, 2, 3, 4, ...). For $s_{f}=1$, both grids share the same resolution; for $s_{f}>1$, the gradient grid is coarser. Consequently, each gradient grid cell corresponds to $s_{f}^{2}$ (1, 4, 9, 16, ...) ABM grid cells.

### Initialisation

The model initialisation proceeds in several sequential steps:

1. Creation of the ABM grid with desired size *L* and corresponding number of grid points.
2. Assignment of the same negative value *k*_0_ (grid strength) to all grid points.
3. Definition of the initial colony geometry by setting all grid points to zero within a circle with radius $r_{colony}$ from the centre of the ABM grid.
4. Random placement of *N* agents on walkable grid points (*k* = 0) with random orientations drawn from a uniform distribution.
5. Creation of the gradient grid, scaled to the ABM grid with factor $s_{f}$ and initialised with a small, randomly distributed chemical concentration drawn from a uniform distribution on the interval $\left[10^{-6},10^{-5}\right]$. This was chosen instead of an initialization with zero to avoid numerical errors that can occur due to limited floating point precision and subsequent solving of equation 2 at each grid point.

### Simulations

**Fig 3:**
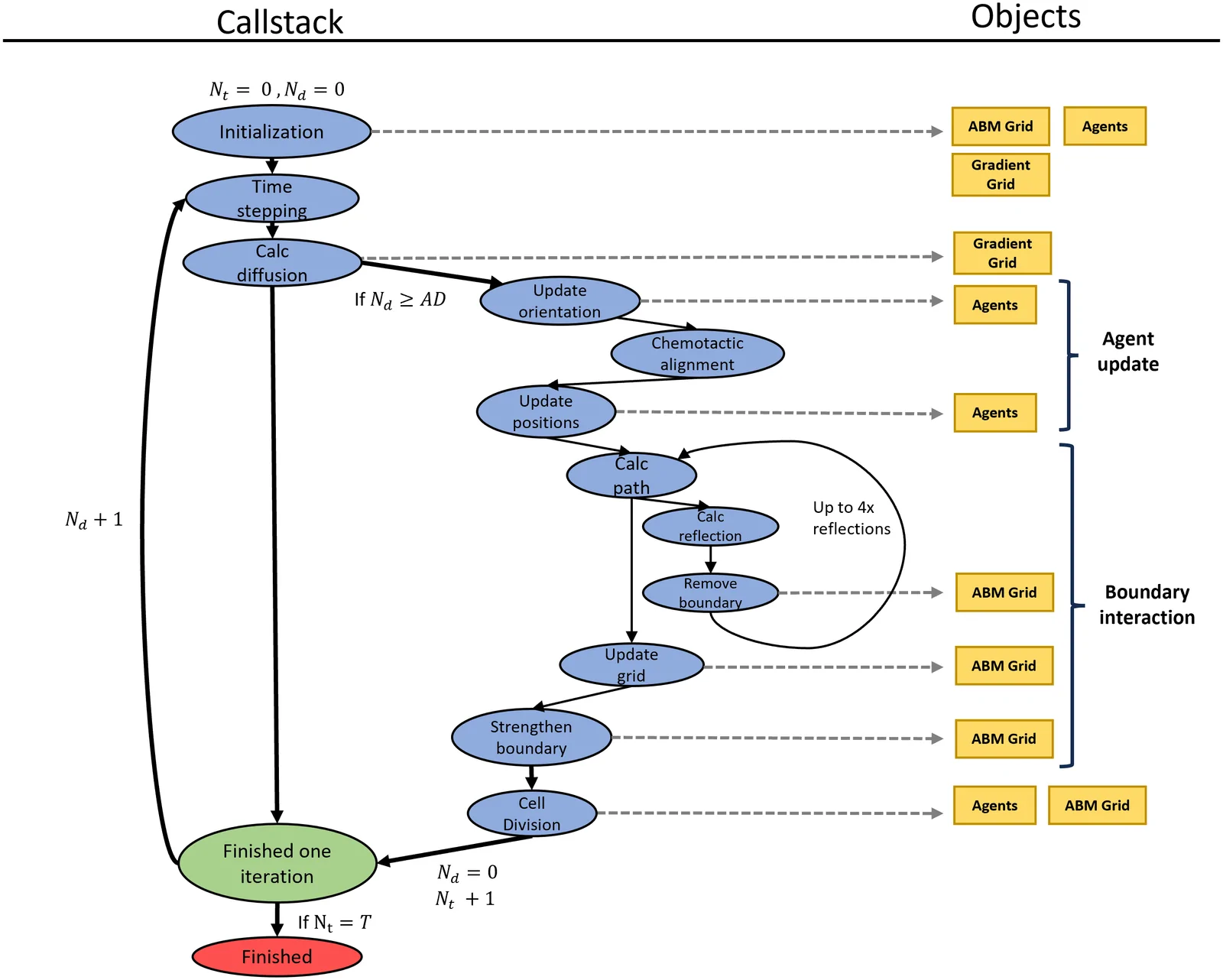
Flowchart of the internal model logic. The blue ovals represent functions that are called in consecutive order, and the yellow boxes represent the objects that they modify. The green oval represents the end of one iteration and the red oval represents the end of one model run. The model has two internal counters: $N_{t}$, which counts the number of agent time steps $\Delta t_{a}$, and $N_{d}$, which counts the number of diffusion time steps $\Delta t_{d}$ per $\Delta t_{a}$ to be exactly *AD*.

The simulations are performed in an iterative manner. Updates of the gradient grid, the agents, and the ABM grid are combined (Fig 3). In the following, we describe the individual parts in detail.

#### Time stepping.

The model updates its components using two different time steps. The first, $\Delta t_{d}$, governs the time evolution of the gradient grid. The second time step, $\Delta t_{a}$, governs updates to agent positions, orientations, and boundary strengths in the agent-based model (ABM) grid. This separation of time scales allows for efficient simulation while maintaining the accuracy of both the chemical diffusion dynamics and agent movement. The ratio of the two time steps $\frac{\Delta t_{d}}{\Delta t_{a}}$ is set as *AD*. Hence, during one iteration, we perform *AD* diffusion steps and one step of updating the agents and the ABM grid (Fig 3).

#### Calculate diffusion.

The model computes the evolution of the chemical field s(x,t) on the gradient grid in space (x) and time (*t*) with a two-dimensional reaction-diffusion equation [16,21,44–47] with diffusion constant $D_{s}$, decay ra*t*e $d_{r}$ and adsorption $a_{d}$ caused by the *N* agents at their ABM grid cells $x_{n}$. As $s_{f}^{2}$ ABM grid cells are mapped to one gradient grid cell, the adsorption is normalized by that factor


$$\begin{array}{c} \frac{\partial s(x,t)}{\partial t}=D_{s}\nabla^{2}s(x,t)-d_{r}s(x,t)+\frac{a_{d}}{s_{f}^{2}}\sum_{n=1}^{N}\delta (x-x_{n}). \end{array}$$ (1)

This equation is discretized and numerically solved using the finite difference method FTCS (forward time-centered space) with Dirichlet boundary conditions where the value of *s* on the boundary is fixed to its initialization value, implemented using the Julia packages ParallelStencils and ImplicitGlobalGrid [48]. This method is commonly used for solving parabolic differential equations [49–51] and was specifically chosen because its time-explicit nature allows easier integration with the agent dynamics compared to implicit methods. The discretized equation


$$\begin{array}{c} s_{i,j}^{t+\Delta t_{d}}=s_{i,j}^{t}+D_{s}\Delta t_{d}\left(\frac{s_{i+1,j}^{t}-2s_{i,j}^{t}+s_{i-1,j}^{t}}{\Delta x^{2}}+\frac{s_{i,j+1}^{t}-2s_{i,j}^{t}+s_{i,j-1}^{t}}{\Delta y^{2}}\right)\\ -d_{r}\Delta t_{d}\;s_{i,j}^{t}+\frac{a_{d}}{s_{f}^{2}}\Delta t_{d}\sum_{n=1}^{N}\delta (x_{i,j}-x_{n}), \end{array}$$ (2)

calculates the concentration *s* at each grid cell with indices (*i*,*j*) at time $t+\Delta t_{d}$, which changes from the value of $s_{i,j}^{t}$ through diffusion from or to neighboring grid cells, the decay of the chemical with rate $d_{r}$, and the adsorption of the chemical with rate $a_{d}$. The Dirac delta function (δ(·)) is only non-zero when $x_{n}$ is one of the $s_{f}^{2}$ ABM grid cells mapping to the gradient grid cell with position $x_{i,j}$ (Fig 2).

The maximum time step size $\Delta t_{d}$ to ensure numerical stability can be derived using von Neumann stability analysis [51–53] (see section A.2 in S1 Appendix for more details)


$$\begin{array}{c} \Delta t_{d}\leq \frac{\Delta x^{2}}{4D_{s}+\frac{d_{r}\Delta x^{2}}{2}}. \end{array}$$ (3)

#### Agent update.

In every time step $\Delta t_{a}$, each agent updates its orientation and position. First, the orientation is updated


$$\begin{array}{c} \theta_{i}(t+\Delta t_{a})=\theta_{i}(t)+\alpha \frac{∠\left([\begin{array}{c} \cos\theta_{i}(t)\\ \sin\theta_{i}(t) \end{array}],-\nabla s(x_{i})\right)}{N_{o}(\nabla s,x_{i})}\Delta t_{a}+\eta_{i}(t)\Delta t_{a}, \end{array}$$ (4)

with turing rate α, noise strength $\eta_{i}$ and the angle between two vectors is calculated as


$$\begin{array}{c} ∠(\vec{g},\vec{u})=\text{atan2}(\vec{g}\times \vec{u},\vec{g}\cdot \vec{u}), \end{array}$$ (5)

with the normalization function


$$\begin{array}{c} N_{o}(\nabla s,x)=\frac{\left|\nabla s(x)\right|}{\max(|\nabla s|)}. \end{array}$$ (6)

The agents try to turn their current direction towards the negative gradient of the chemical field $s(x_{i})$ at their position $x_{i}$. The speed of this negative chemotactic alignment process is determined by the maximum gradient in the system through the normalization factor $N_{o}$ and a tunable turning rate parameter α∈[0,1], creating a relative response: For the case where α=1, an agent at the location of the steepest gradient in the system will completely align its direction in one time step, while agents in regions with weaker gradients will align proportionally slower. When α<1, the alignment speed is linearly scaled down by this factor. The term $\eta_{i}(t)$ models the stochastic component of the agent’s movement and alignment process and is drawn from a normal distribution with mean $0^{\circ }$ and standard deviation η.

Each agent’s position is updated with


$$\begin{array}{c} x_{i}(t+\Delta t_{a})=x_{i}(t)+v_{i}\Delta t_{a}[\begin{array}{c} \cos\theta_{i}(t+\Delta ta)\\ \sin\theta_{i}(t+\Delta ta) \end{array}]. \end{array}$$ (7)

The agents move from their current position $x_{i}(t)$ in the direction $\theta_{i}(t+\Delta t_{a})$ with velocity $v_{i}$, which is drawn from a normal distribution with mean *v*_0_ and standard deviation $v_{\sigma }$. Since $x(t+\Delta t_{a})$ is a vector of two continuous values, it is rounded to the nearest discrete grid cell on the ABM grid, whereas $\theta_{i}(t+\Delta t_{a})$ is not rounded and stored in the agent table. Such an update scheme would be labeled as microscopic dry active matter [41], or in other terminology, modified active Brownian particles with auto-chemotactic alignment.

The value of the time step $\Delta t_{a}$ must strike a balance between two opposing constraints. If $\Delta t_{a}$ is too small, the time discretization becomes overly fine, potentially introducing discretization artefacts: agents may move only one grid cell, or remain stationary, per step, which distorts movement dynamics [54]. Conversely, if $\Delta t_{a}$ is too large, agents may traverse unrealistically long distances in a single step. This results in other discretization artefacts, such as unnaturally straight trajectories, which contradict experimental observations of cell motility [4]. In addition, rapid changes in the local environment, such as the number and identity of neighbouring agents or proximity to boundaries, impair biologically plausible agent-agent interactions. To balance these constraints, $\Delta t_{a}$ was chosen to allow agents to move on average three ABM grid cells per time step in their current direction. This corresponds to a distance of 11.25μm, which is shorter than the typical cell length of a procyclic trypanosome (~15−25μm [4,55]), thereby preserving local agent-agent interactions. For typical model parameters, the ratio $AD=\frac{\Delta t_{a}}{\Delta t_{d}}$ ranges between 1 and 300. If necessary, the diffusion time step $\Delta t_{d}$ is slightly reduced from its maximum allowable value to ensure that *AD* is always an integer. This guarantees a consistent number of diffusion time steps $\Delta t_{d}$ per agent time step $\Delta t_{a}$ throughout the simulation.

#### Boundary interactions.

**Fig 4:**
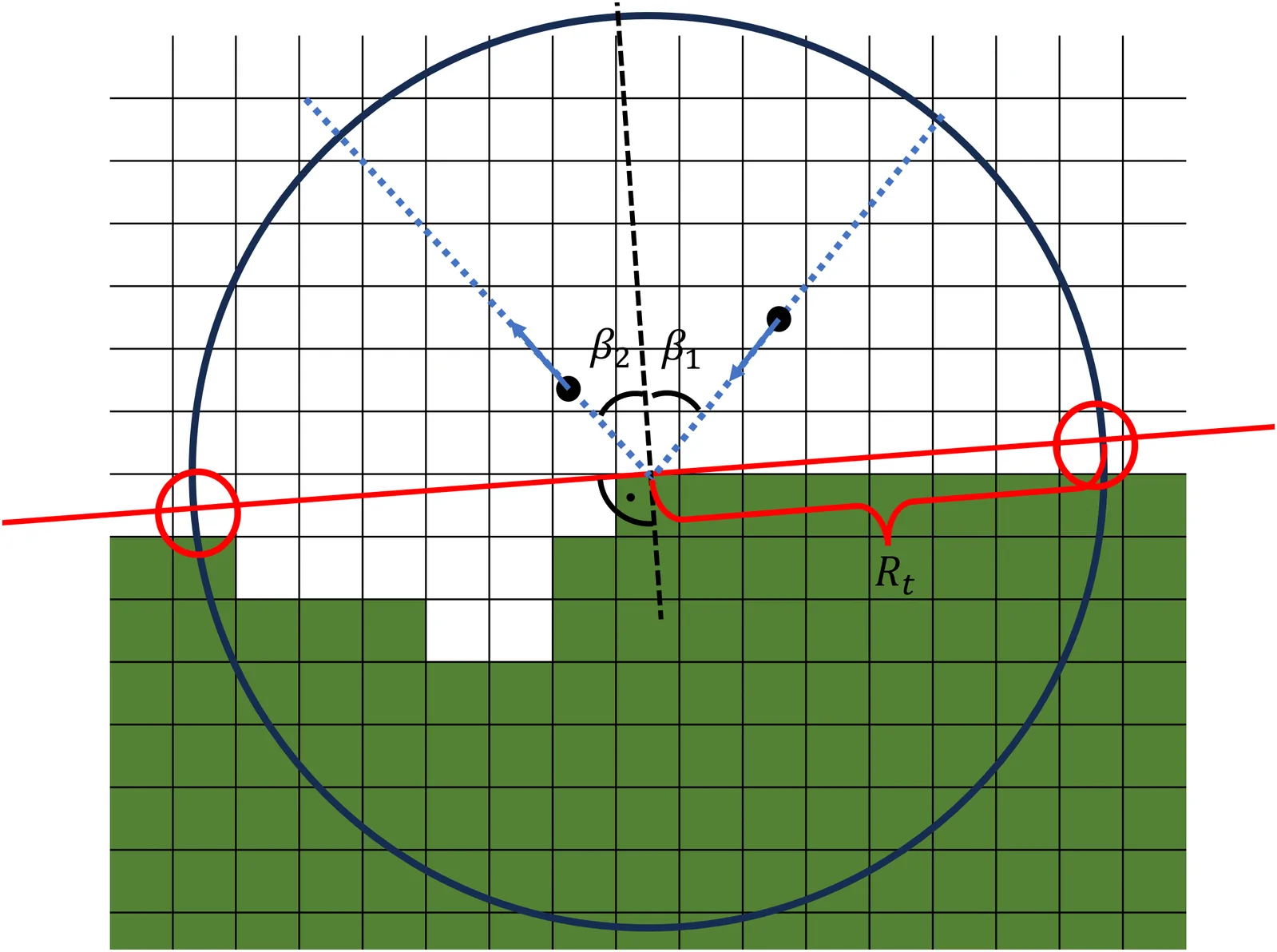
Schematic representation of the boundary reflection mechanism. White grid cells represent empty (walkable) regions, whilst green cells are non-walkable, forming the colony boundary. To approximate the local boundary morphology at the collision point, a circle of radius $R_{t}$ is centred on the collision point. The two boundary grid cells on this circle that are most distant from each other define a tangent line that approximates the boundary morphology. The agent (black dot with blue arrow) then reflects off this tangent with outgoing angle $\beta_{2}$ equal to incoming angle $\beta_{1}$, analogous to specular reflection.

The model simulates agent interactions with the colony boundary through reflective boundary conditions, whereby agents are reflected upon encountering a non-walkable grid cell on their path. Whilst real trypanosomes do not undergo physical reflection from colony boundaries, they typically remain at boundaries for only brief periods (a few seconds) before reorienting [4]. Therefore, reflective boundary conditions represent a justified simplification that captures the average behaviour at the agent numbers simulated here. The lattice constant Δx and time step $\Delta t_{a}$ are set to values so that agents move an average of three grid cells per time step. Since their movement paths can therefore interfere with non-walkable grid cells with *k* < 0, their movement paths are divided into segments of single grid cells. At each segment, the model checks for the presence of non-walkable grid cells with *k* < 0. If one is encountered, the agent is reflected. The reflection process approximates the local boundary morphology by constructing a circle with radius $R_{t}$ around the collision point (the first grid cell in an agent path segment with *k* < 0). The surface tangent is approximated using the two most distant non-boundary points neighbouring a boundary point. The agent is then reflected off this tangent such that the incoming angle $\beta_{1}$ equals the outgoing angle $\beta_{2}$ (Fig 4).

**Fig 5:**
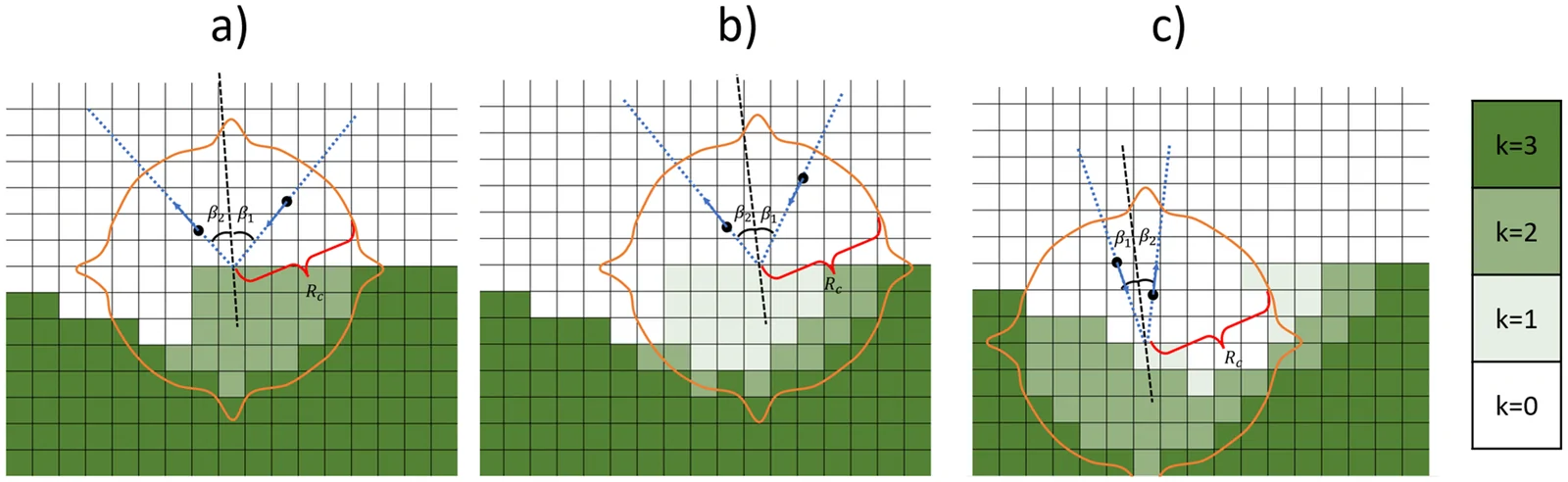
Schematic representation of the boundary removal mechanism for an initial boundary strength k=−3 and three subsequent agent collisions (a, b, c). Upon agent collision with a boundary, the boundary strength *k* increases by 1 in all boundary cells within a hybrid shape combining a circle and a diamond (see section A.5 in S1 Appendix for more details) with radius $R_{c}$. When *k* reaches zero after multiple collisions, boundary cells are converted to walkable space.

If an agent collides with a non-walkable grid cell on the colony boundary, the boundary weakens (Fig 5). This process is modelled through a change in the value *k*, which is initialized at all non-walkable grid cells with a negative number *k*_0_ representing the grid strength. Upon collision, all grid cells with *k* < 0 within a circle of radius $R_{c}$ (distinct from $R_{t}$) centred on the colliding agent have their *k* values increased by 1. To address lattice anisotropy effects, not a perfect circle with radius $R_{c}$ was used, but rather a hybrid shape combining a circle and a diamond (see section A.5 in S1 Appendix for more details). If *k* reaches zero, the non-walkable cells become walkable space and are removed from the boundary. Similar approaches have been successfully used to model bacterial colony growth [22,44]. Due to potentially complex local boundary morphology forming structures such as narrow channels, this collision-reflection-weakening process can theoretically occur many times in a single time step, but never exceeded four iterations in any simulation (Fig 3). Such complex morphology can also create cases where no unambiguous tangent can be determined; in these situations, a fallback mechanism is invoked where the agent is not reflected but simply reverses its original direction. This occurred on average once per 10^8^ agent collisions but is highly dependent on morphology.

Together, the grid strength *k*, the grid recovery rate $g_{rc}$, and the agent collision mechanism constitute a phenomenological model of the complex hydrodynamic interface between the semi-fluid medium (agarose) and the fluid medium (nutrient solution containing trypanosomes). This interface can exchange liquid vertically to increase the volume of the fluid medium whilst remaining laterally separated and maintaining cohesion through surface tension. Meanwhile, the trypanosomes actively drive outward expansion of the fluid medium through organised movement.

#### Cell division.

Cell growth is modelled as exponential, based on population doubling times $\tau_{d}$ observed in experiments [3,4,34]. From the doubling time, we derive the growth rate *r*


$$\begin{array}{c} r=2^{\frac{1}{\tau_{d}}}-1. \end{array}$$ (8)

In each time step $\Delta t_{a}$, each agent has a probability $r\cdot \Delta t_{a}$ of dividing and forming a new agent in a neighbouring empty grid cell. If no neighbouring empty grid cell is available, the agent cannot divide due to spatial constraints. The new agent is assigned a random orientation independent of the parent agent. Throughout the results and discussion sections, we primarily use the doubling time $\tau_{d}$ rather than the growth rate *r* to describe growth dynamics, as it provides a more intuitive interpretation of population expansion.

### Model visualisation

**Fig 6:**
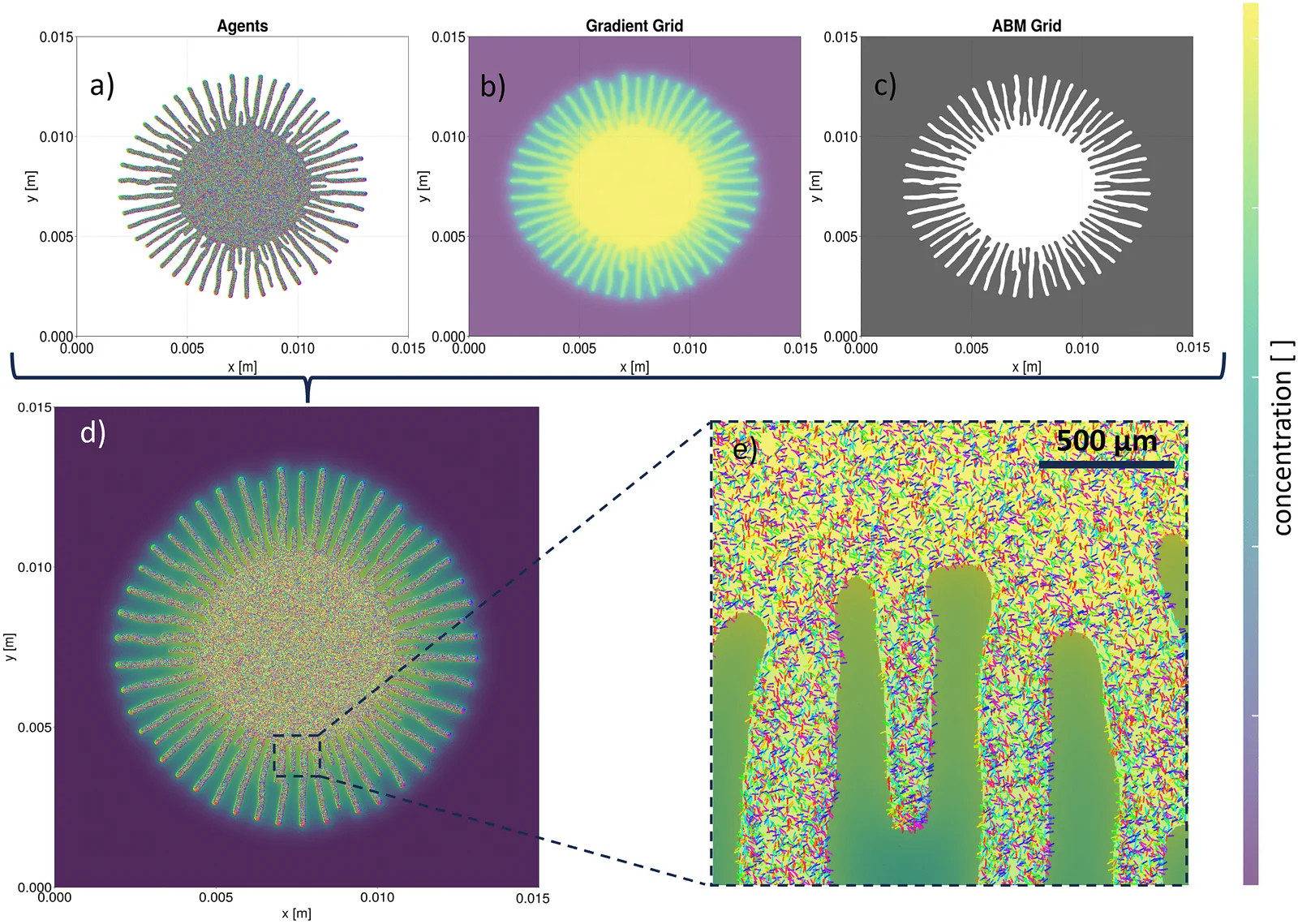
Visualisation scheme of one model simulation after 9 h with default parameters (Table 1) and *L* = *x* = *y* = 15 mm. (a) The $\sim 5\times 10^{5}$ agents are displayed as a vector plot where each agent is represented by an arrow pointing in its current direction. The direction is additionally colour-coded. Due to the high number of agents, it is not possible to distinguish individuals. (b) The gradient grid is visualised by a heat map where relative concentration values map to colours (colourbar on the right), with yellow representing the highest concentration in the system and purple the lowest. (c) The ABM grid is displayed as a binary mask where non-colony regions (*k* < 0) are shaded grey and regions within the colony (k≥0) are transparent. (d) The three components of the model are plotted together in a single plot containing all information. (e) Zoomed-in view of the complete plot to better visualise individual agents in their crowded environment. The agents are colour-coded according to their direction.

The visualisation integrates all three components of the model into a single composite image (Fig 6). The agents are represented by a vector plot, where each arrow corresponds to one agent. These arrows indicate the current direction of movement, with additional colour-coding to distinguish agent orientations.

The chemical gradient is displayed as a heat map with concentration values mapped to a colour scale. Since it is not yet clear which chemical is responsible for chemotactic alignment in trypanosome colonies and concentrations for the candidate chemicals have not been measured either, the absolute values of the concentration are less important than the relative concentration gradients in the system, which drive agent behaviour. Therefore, in all visualisations, the colourbar is auto-scaled to the maximum concentration present in each specific image. This approach allows us to visualize the relative concentration gradients consistently across all images using the same colour scaling, even though the absolute concentration values may differ substantially between different simulation conditions or time points. The ABM grid is visualized as a binary mask where non-colony regions appear gray while colony regions are transparent. These three visual elements are layered to provide a comprehensive view of the entire system, enabling intuitive observation of emerging patterns during simulation.

Most images shown in the subsequent chapters do not display individual agents, as it is not possible to discern individual agents for simulations with realistic agent numbers, and most analysis in the following sections focuses primarily on the morphology of the ABM grid.

### Implementation

The code for implementing the model and the simulations was designed with modularity and expandability as primary considerations. While this approach introduces some computational overhead, as some code parts are not optimized for maximum memory efficiency, it offers significant advantages for future development. The data structures have been chosen to allow a straightforward integration of additional model parameters or agent attributes in subsequent model iterations.

The code is written in the programming language Julia [56], which aligns with the model’s design philosophy. Julia combines the readable, accessible syntax of high-level languages with performance comparable to traditional compiled languages like C and Fortran. This combination is particularly valuable for scientific computing applications where accessibility and runtime performance are critical. Julia’s growing ecosystem of scientific libraries also provides valuable tools for efficient numerical computations, particularly for the reaction-diffusion equation. The code is available at https://github.com/AndreasKuhn-ak/2026-Kuhn-et-al and will be archived at Zenodo upon publication.

### Simulation modes

The simulations can be run in two distinct modes:

The first is the interactive mode, where only the current state of the simulation is stored in memory and the output is displayed with live animation using the **Makie.jl** plotting package [57]. In this mode, the parameter values can be dynamically adjusted during the simulation run to study their influence on model behaviour. The interactive mode can simulate up to 10^6^ agents in real-time on a Ryzen 7 9700X 8-Core CPU and 64 GB RAM @ 6000 MHz or a comparable model, meaning one second in the model corresponds to one second or less in real time. The computation times of the model scale almost linearly with the number of agents and grid cells used.

The second is the non-interactive mode, where the simulation runs with predetermined parameter values without graphical output. Here, the state of the simulation is saved at specified time points for later analyses. This mode is approximately twice as fast as the interactive mode and is primarily used for parameter sweeps in multiple parallel instances on workstations or clusters with sufficient memory per core. The saved data are subsequently used for analyses and visualisation.

### Parameter space

The model possesses a large parameter space, the parameters of which can be classified into four distinct categories:

(a) Parameters corresponding to measurable quantities that have been experimentally determined (e.g., growth rate *r* of trypanosomes in social motility assays). These experimentally measured values were adopted as default values in the model.
(b) Parameters corresponding to measurable quantities that have not yet been measured (e.g., adsorption rate $a_{d}$, decay rate $d_{r}$, and diffusion constant $D_{s}$ of the proposed chemotactic substance [33,35]). The reasons these remain unmeasured vary, but generally stem from either measurement difficulties or a lack of prior scientific motivation to quantify them.
(c) Parameters that do not directly correspond to measurable physical quantities (e.g., boundary strength *k* and grid recovery rate $g_{rc}$). These parameters arise from simplifications in the model. For example, the real colony boundary represents a complex hydrodynamic interface between a semi-fluid (agarose) and a fluid medium (nutrition solution containing trypanosomes), which can exchange liquid while remaining separated by surface tension. Although the properties of this interface could theoretically be measured, incorporating them would require modelling the interface with comparable complexity.
(d) The discretisation of time and space is also a model parameter; the values chosen/calculated for these are explained above and in section A.1 in S1 Appendix.

For parameters in categories (b) and (c), default values were determined through extensive testing, primarily in interactive mode. These values were selected to produce colony morphology dynamics similar to those observed and quantified in experiments [5]. A detailed analysis of their influence is shown in the results section.

All parameters are shown in Table 1 together with their category and default values. There are two values given for the doubling time $\tau_{d}$ as different experiments reported either doubling times of 9 h [3,34] or 20 h [4]. We tested both values. The time steps $\Delta t_{a}$ and $\Delta t_{d}$ are dynamically calculated quantities that change depending on the diffusion coefficient $D_{s}$, the mean agent velocity *v*_0_, the decay rate $d_{r}$, and the lattice constant Δx. Unless otherwise specified, all presented data are from simulated colonies starting with $N=2.5\times 10^{5}$ agents in a circular geometry with a diameter of 3 mm. This represents a similar trypanosome density to that observed in the well-quantified experiments from Kruger et al. [4,5], which serve as a blueprint for our model. In these experiments, approximately 10^6^ agents are situated in colonies with diameters of 6 mm. Initial testing showed that this downscaling of the system by a factor of four did not change the overall behaviour but reduced computation times by a factor of approximately eight. This computational advantage aligns with other experimental observations, which demonstrated unchanged social motility activity in smaller colonies of $N=2\times 10^{5}$ trypanosomes [34], hence its adoption in our simulations.

**Table 1:** Model parameters categorised by type with their default values and units.

Parameter	Description	Type	Default Value	Units	Source
$\tau_{d}$	Doubling time	a	9.0/20.0	*h*	[3,4,34]
*v*_0_	Mean agent velocity	a	5	μm/s	[4]
$v_{\sigma }$	Velocity stdd	a	2.5	μm/s	[4]
$D_{s}$	Diffusion coefficient	b	$7*10^{-11}$	$\frac{m}{s^{2}}$	–
$a_{d}$	Adsorption rate	b	$1*10^{-6}$	s^-1^	–
$d_{r}$	Decay rate	b	$1*10^{-3}$	s^-1^	–
*k*_0_	Boundary strength	c	−8000	–	–
$g_{rc}$	Grid recovery rate	c	7	s^-1^	–
$R_{c}$	Collision radius	c	16	grid cells	–
$R_{t}$	Tangent approx r	c	15	grid cells	–
η	Orientation noise	c	0.2	rad/s	–
α	Turning rate	c	0.1	rad/s	–
$s_{f}$	Grid scaling factor	d	4	–	–
Δx	ABM lattice constant	d	3.75	μm	–
$\Delta t_{a}$	Agent time step	d	2.25	s	–
$\Delta t_{\text{diff}}$	Diffusion time step	d	0.75	s	–

Another performance-relevant quantity to determine before each simulation run is the edge length *L* of the simulated space. The computation time for the diffusion equation on the gradient grid scales with *L*^2^. Since we use Dirichlet boundary conditions, the colony cannot expand beyond the grid boundaries. Therefore, *L* should be large enough to prevent the colony from reaching the boundaries during the simulation period, yet as small as possible to maintain reasonable computation times. After testing, we determined that *L* = 15 mm for simulations running 10 h and *L* = 30 mm for simulations running 20 h satisfied these conditions well, and we used these values throughout our analyses. None of the simulated colonies included in this work reached the grid boundaries.

One important observation from our testing is that the variation between model runs with identical parameters is very small (see section A.4.1 in S1 Appendix for more details). Given this high reproducibility and the significant computational demands, we opted to run each parameter set only once for the simulations presented below, thereby substantially reducing overall computation time without compromising the validity of our conclusions.

### Analysis

The simulation results were analysed by characterising the ABM grid. We measured the area *A* of the colony at each time point as the number of colony grid points and normalised the increase in area with respect to the initial time point. Hence, for a given time point $t^{*}$ and initial time point *t*_0_, we calculated the relative area increase of *t*he simulated colony as $\frac{A(t^{*})-A(t_{0})}{A(t_{0})}$. Furthermore, we counted the number of agents at each time point to obtain the cell number and calculated the sum of adsorbed material on the gradient grid. The cell density was calculated as $\frac{\text{cell number}}{A(t^{*})}$. To quantify the colony morphology, we used surface roughness *W*, coefficient of variation *CV*, relative maximum peak height *MaxP*1, and mean Fourier amplitude $I_{k}$. These morphological metrics are based on an angular metric and a pair correlation metric and were previously introduced for trypanosome colonies [5]. The surface roughness *W* quantifies the overall irregularity of the colony boundary, with higher values indicating more pronounced deviations from circular growth. The coefficient of variation *CV* measures the degree of fluctuation in the radial distribution of colony growth and captures how finger-like the colony structure is. The relative maximum peak height *MaxP*1 indicates the prominence of finger-like structures by measuring how much pixel pairs along the same finger dominate over the average distribution. The mean Fourier amplitude $I_{k}$ quantifies the amount of periodic fluctuations in the colony surface, serving as a measure of how non-circular the expansion pattern is.

## Results

### Simulated colonies show two-phase expansion behaviour

**Fig 7:**
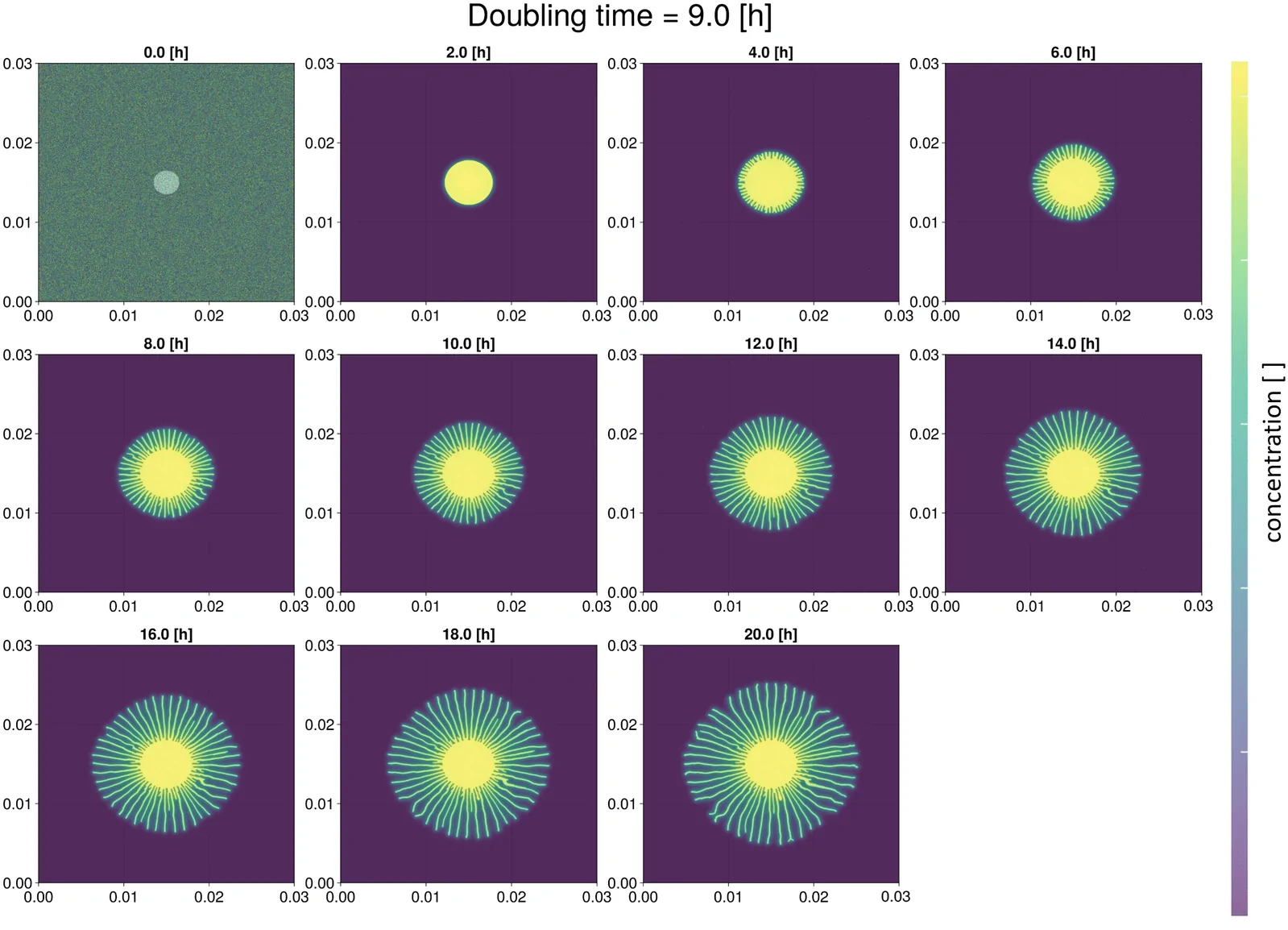
Time evolution of the colony morphology over 20 hours (2-hour intervals) with $\tau_{d}=9$ h and default parameters (Table 1). Initial noise in the gradient grid reflects random initialization. For details of visualisation see Materials and Methods.

**Fig 8:**
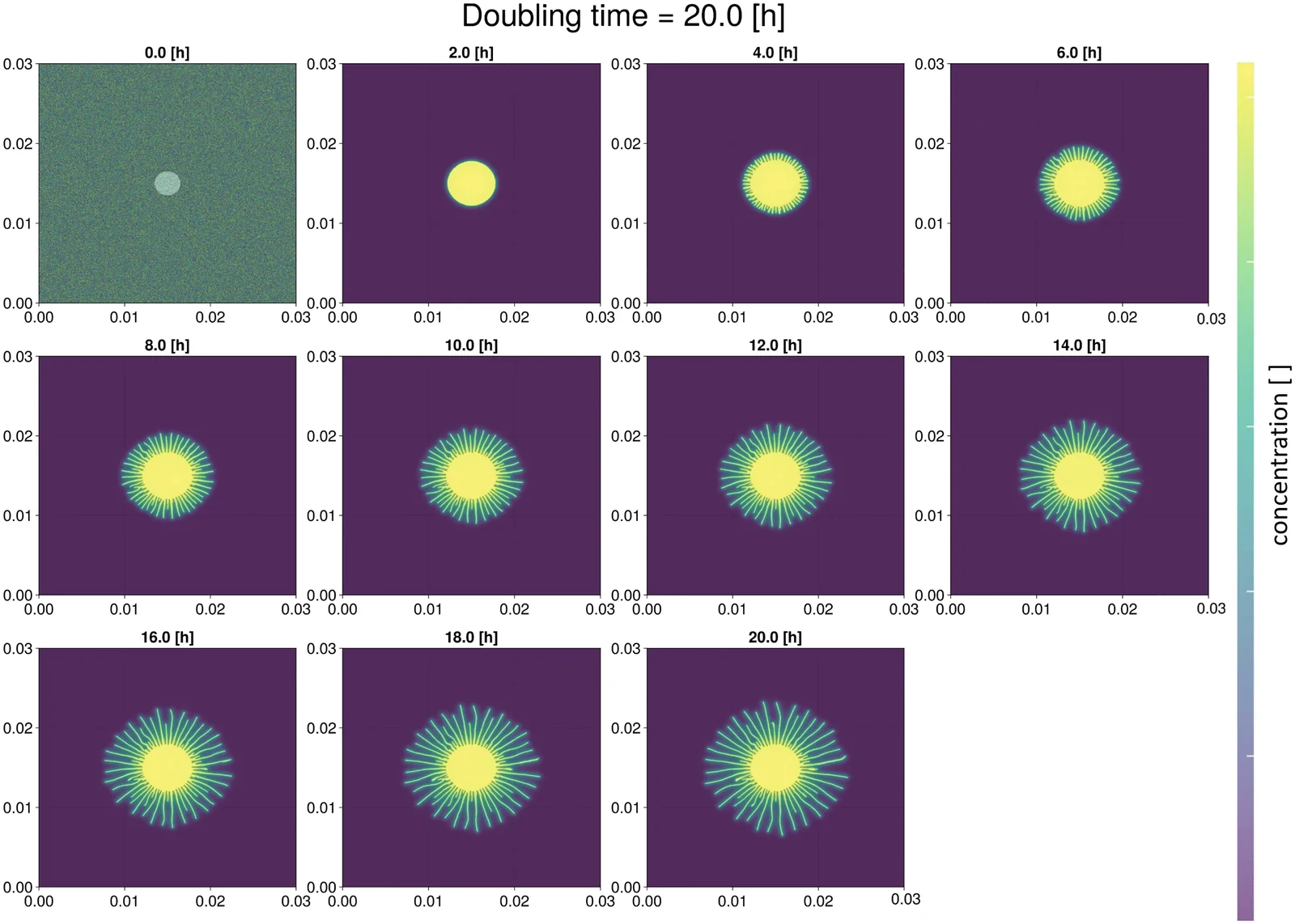
Time evolution of the colony morphology over 20 hours (2-hour intervals) with $\tau_{d}=20$ h and default parameters (Table 1). Initial noise in the gradient grid reflects random initialization. For details of visualisation see Materials and Methods.

To establish baseline behaviour and validate our model against experimental observations, we simulated the system for 20 of simulation time using default parameter values (Table 1) with two biologically relevant doubling times: $\tau_{d}=9$ h (Fig 7) and $\tau_{d}=20$ h (Fig 8), corresponding to different experimental conditions [3,4,34].

Both simulations exhibit a two-phase expansion: initial circular growth (0–2 h) followed by finger-like expansion. The faster-growing colony ($\tau_{d}=9$ h) develops approximately 50 fingers with more uniform morphology, while the slower case ($\tau_{d}=20$ h) produces approximately 45 fingers with greater variability in length.

**Fig 9:**
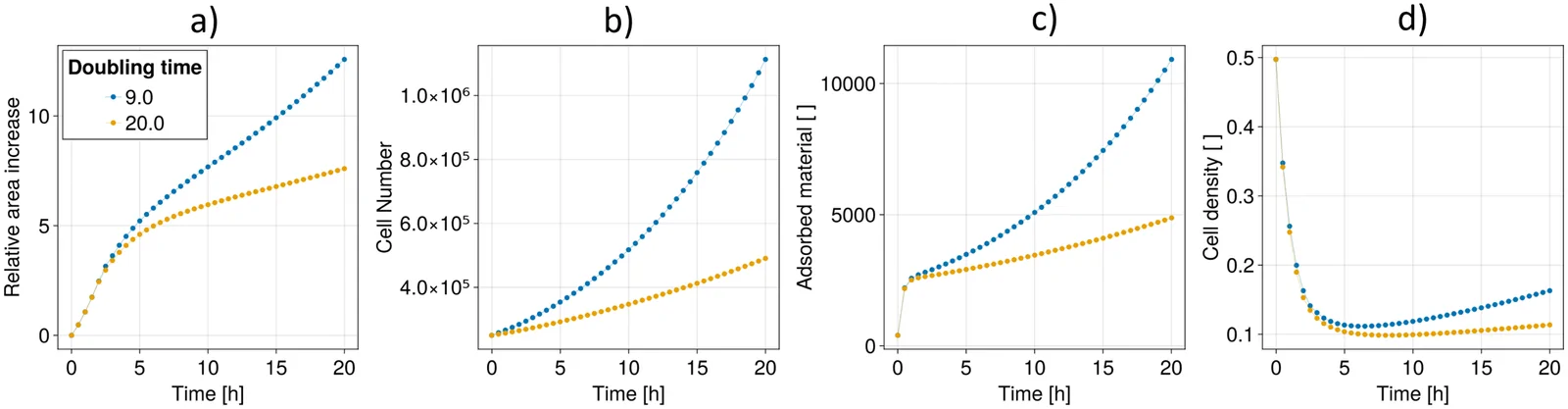
Temporal evolution of colony properties in 30-minute intervals for two doubling times and default parameters (Table 1). The results are for one simulation run each due to very low variability between runs (see Materials and Methods for details). (a) Relative area increase, (b) Cell number, (c) Adsorbed material, (d) Cell density. Note the density minimum at 4 h, coinciding with the morphological transition.

During the circular phase (0–2 h), the relative area increase is very rapid and nearly identical across colonies, despite differences in doubling time (Fig 9). This occurs because the initial strong gradient at the colony boundary that drives outward expansion is produced primarily by the starting cell population rather than by cells added through division. The total cell number increases exponentially with a constant growth rate. Decay and adsorption balance within the first hour; thereafter, the amount of adsorbed material increases exponentially, mirroring cell growth. Cell density reaches a minimum during the first 10 h as colony expansion slows when fingers begin to grow, weakening the local gradient at the boundaries through the increased surface area, whilst cell number continues to increase at the same rate. All measures increase more rapidly in the faster-growing colony.

**Fig 10:**
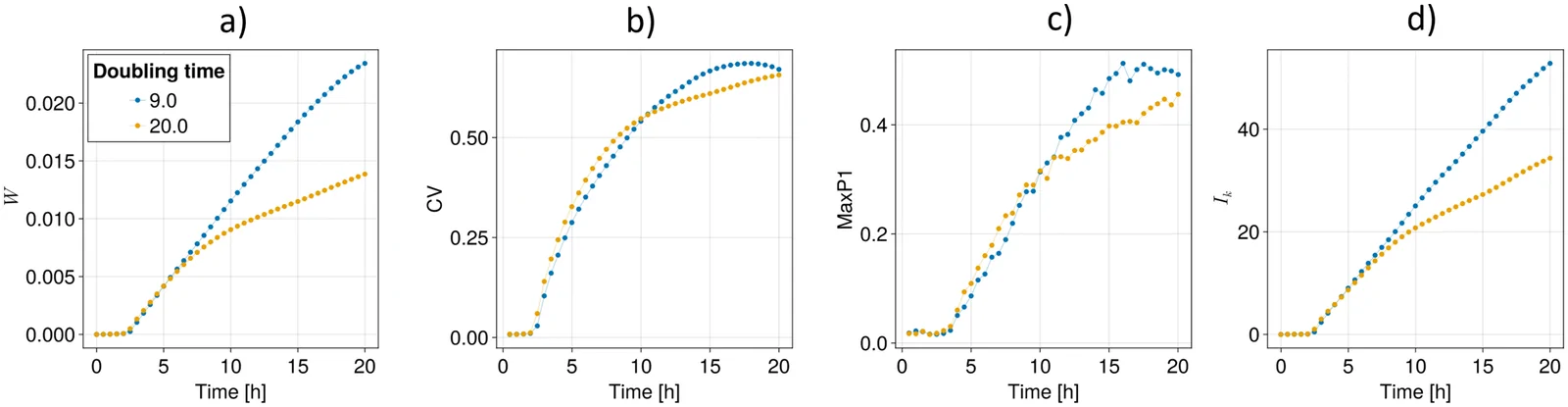
Temporal evolution of morphological metrics in 30-minute intervals for two doubling times and default parameters (Table 1). The results are for one simulation run each due to very low variability between runs (see Materials and Methods for details). (a) Surface roughness *W*, (b) Coefficient of variation *CV*, (c) Relative maximum peak height *MaxP*1, and (d) Mean Fourier amplitude $I_{k}$.

We employed our established morphological metrics that are able to quantify the change from circular to finger-like growth by asserting values of zero to a colony growing perfectly circular and increasing to higher values for more finger-like growth (Fig 10, [5]).

All metrics clearly detect the initial circular growth phase, showing values at or near zero during the first two hours of the simulations. Subsequently, all metrics capture the transition from circular to finger-like growth as their values begin to increase. The coefficient of variation of the angular metric (*CV*) shows the highest sensitivity to this transition as it already increases at 1.5 h.

The roughness (*W*) and the mean Fourier amplitude ($I_{k}$) exhibit patterns of steady increase for both doubling times. In contrast, the *CV* and *MaxP*1 metrics increase more slowly after 10 h. For $\tau_{d}=9$ h, they eventually saturate. Both quantities particularly represent expansion that is perpendicular to the center point of the colony at *t* = 0 h [5]. After 10 h, this becomes less pronounced because single fingers begin to diverge from the initial straight pa*t*hs and create a less uniform expansion front (Fig 7). In summary, the model exhibits the two-phase expansion behaviour observed *in vitro*. Our established metrics for *in vitro* Trypanosoma colonies successfully quantify the behaviour of the *in silico* colonies [5].

### Simulations can reproduce experimental morphological metrics

We compared our results with experimental data from previous work [4,5] (see Section A.3 in S1 Appendix for details of the experimental conditions and measurements). Visual inspection reveals qualitatively similar colony morphologies, though the simulations produce more fingers than observed experimentally. The model also reproduces the experimentally reported perpendicular alignment of trypanosomes at the colony boundary (Fig 6, [4]).

**Fig 11:**
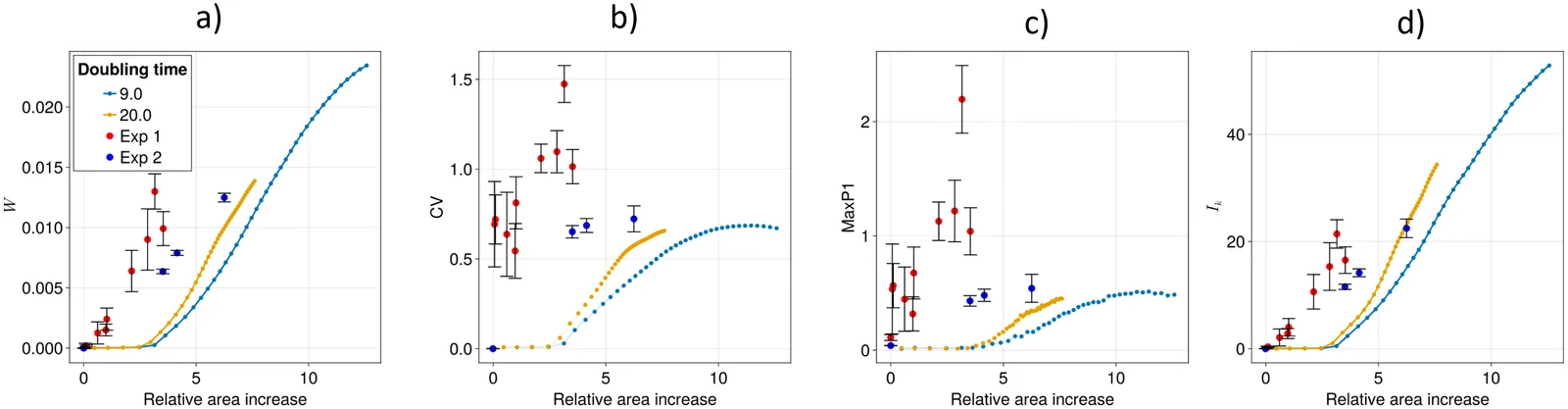
Evolution of morphological metrics relative to area increase for default parameter values (Table 1) with doubling times of $\tau_{d}=9$ h and $\tau_{d}=20$ h and for two experimental data sets (Exp 1 and Exp 2 both with $\tau_{d}\approx 21$ h) [54]. The dots indicate the mean and the error bars the standard deviation. (a) Surface roughness *W*, (b) Coefficient of variation *CV*, (c) Relative maximum peak height *MaxP*1, and (d) Mean Fourier amplitude $I_{k}$.

For a quantitative comparison, we consider two experimental conditions [5]. In Exp 1, 10^6^ cells were seeded, yielding a relative area increase of 1 and approximately 15 fingers after 20 h. In Exp 2, twice as many cells were seeded, yielding a relative area increase of 3.5 and approximately 30 fingers after 20 h. The simulated colonies grow considerably faster, reaching relative area increases of 7.5 and 13 for doubling times of $\tau_{d}=20$ h and $\tau_{d}=9$ h, respectively (Fig 9). To enable meaningful comparison of the morphological metrics despite these differences in growth speed, we plot all data against relative area increase rather than time (Fig 11).

The closest agreement across all four metrics (*W*, *CV*, *MaxP*1, $I_{k}$) is observed between Exp 2 and the $\tau_{d}=20$ h simulation for higher relative area increases, consistent with their finger counts (~30 in Exp 2 and ~45 for $\tau_{d}=20$) being the most similar between experiment and simulation. The remaining discrepancy at low relative-area increases is explained by the more pronounced circular expansion phase in the simulations, which delays the onset of fingering and shifts the increase in the metrics to larger relative-area values than in the experiments. This offset aside, the slope of the metric curves is similar between simulations and experiments. For Exp 1, the discrepancies are larger, which we attribute to the greater difference in finger count. Accordingly, *CV* and *MaxP*1, which are more sensitive to finger number, show the largest deviations, whereas *W* and $I_{k}$ remain more consistent across all conditions and clearly display the characteristic delayed onset followed by a similar slope.

Two non-exclusive explanations may account for the remaining quantitative discrepancies. First, more finely tuned parameter values, particularly the agent movement parameters α and η and/or a smaller adsorption rate $a_{d}$, may bring the simulated finger count and growth dynamics closer to the experimental observations. Second, model misspecification cannot be excluded: the current model neglects nutrient depletion and describes the colony boundary in a simplified, phenomenological manner, both of which could alter the transition from circular to fingered expansion. Despite these quantitative differences, simulations and experiments show consistent qualitative behaviour, with colonies transitioning from circular expansion to a fingered morphology in a manner that depends systematically on growth rate and initial cell density.

### Parameter sensitivity analysis

As the baseline behaviour was established, we performed an initial testing phase with the interactive simulation mode (see Materials and Methods for details). We then systematically varied model parameters that showed the most influence on the behaviour, or are mapped directly to open questions in the field. As a compromise between simulation runtime and realism, all subsequent simulations used $\tau_{d}=9$ h as the default doubling time and were run for 10 h, as this duration was sufficient for the characteristic two expansion phases to emerge (Fig 7). We started our analysis with the agent’s movement, which is mostly determined by the orientation noise η and the turning rate α (Equation (4)).

#### Orientation noise affects colony morphology.

**Fig 12:**
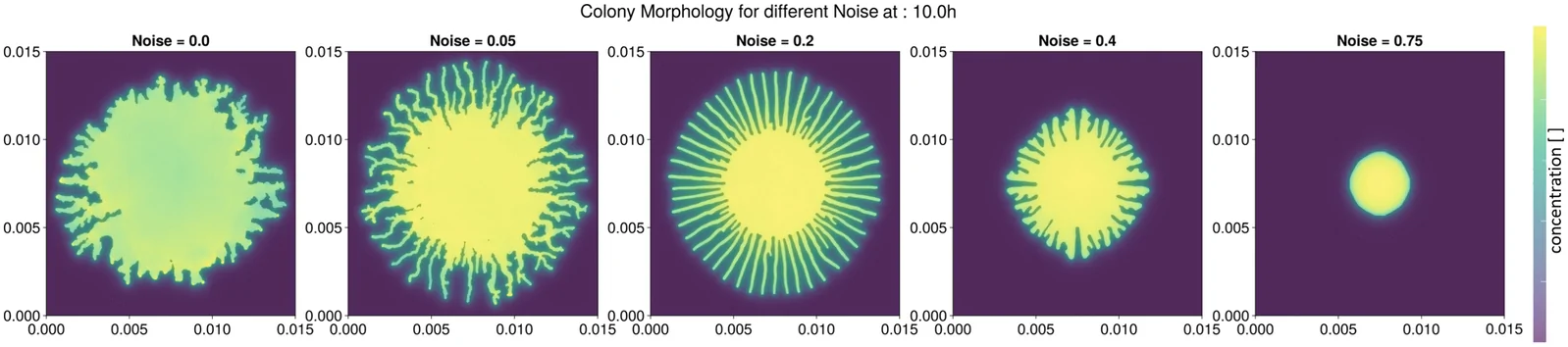
Colony morphology for five different values of the orientation noise η after 10 h with default parameters (Table 1). For details of visualisation see Materials and Methods.

The parameter η is the stochastic component of the direction alignment. The two edge cases η=0 and η=2π represent perfect chemotactic alignment and complete random direction assignment at every time step, respectively. Neither extreme represents realistic microswimmer behaviour, as their active propulsion always has a stochastic components [41]. We varied η in 16 steps over a range of 0.0 – 2π and analysed five parameter values in that range that represent the different model behaviours observed in that range (Fig 12).

For η=0, the colony expands rapidly but irregularly, with small, irregular fingers of varying sizes. This is at first glance counterintuitive, as lower noise should lead to more regular patterns. However, stuck states can emerge: clusters of agents of various sizes form when agents cannot move because their target grid positions are already occupied. In a noiseless environment, there is no mechanism to quickly resolve such states. These heterogeneously distributed clusters generate locally very strong gradients that influence the expansion behaviour of surrounding agents. Whilst exploration of such states might be theoretically interesting, this represents an artefact that would never occur in a real, noisy biological system. Increasing the noise slightly to η=0.05 creates a more uniform gradient and more regular, finger-like expansion by mostly avoiding the aforementioned artefacts. At η=0.2, the expansion pattern becomes much more regular, resembling the patterns observed in *in vitro* colonies. For higher noise values like η=0.4, expansion is significantly slower, and fingers are less distinct. At η=0.75, fingers disappear entirely, and expansion becomes exclusively circular and much slower as the agents now move in an essentially random manner and do not align with the gradient.

**Fig 13:**
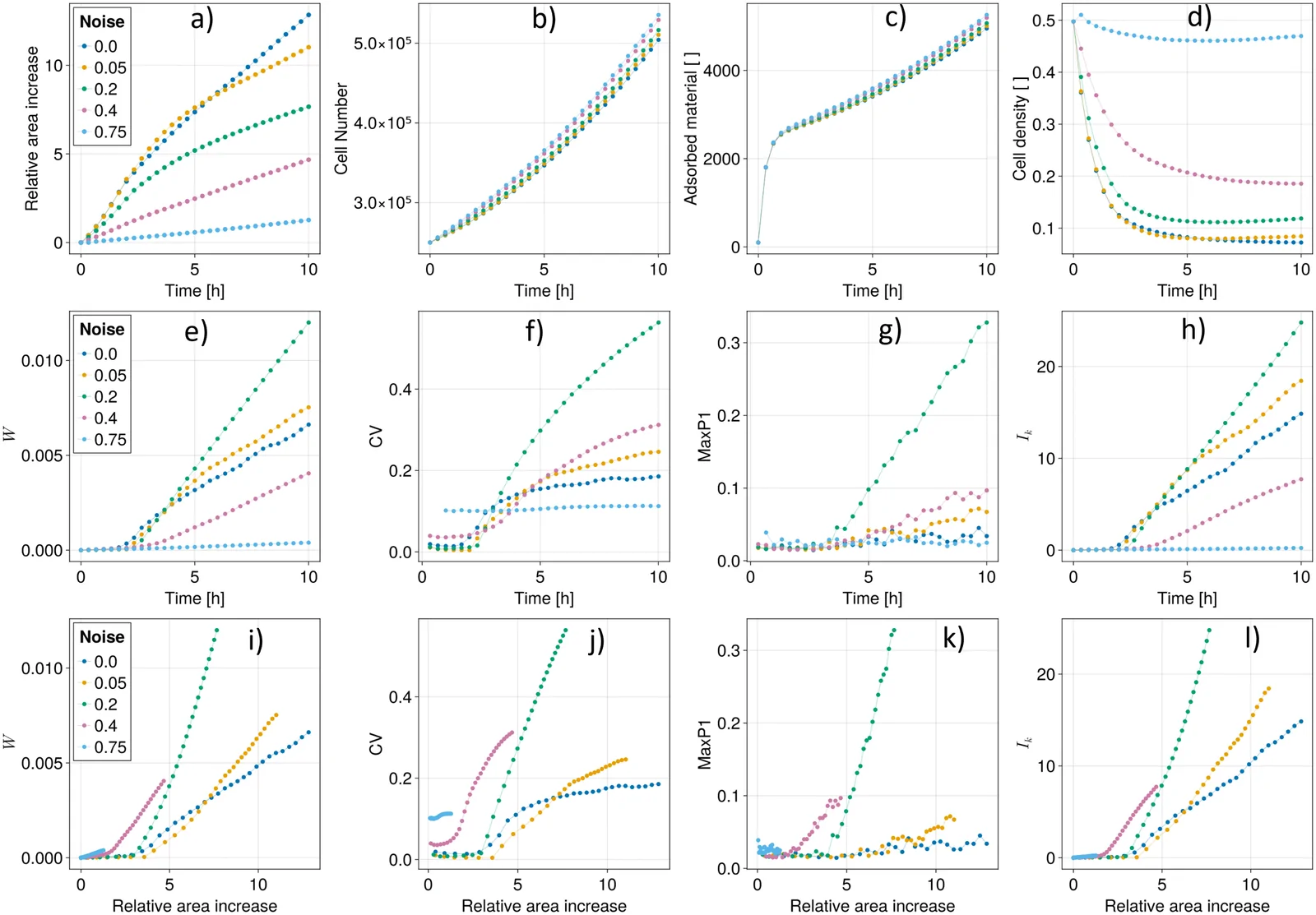
Temporal evolution of morphological metrics over 10 h for default parameters (Table 1) with five different noise values in 20-minutes intervals. The results are for one simulation run each due to very low variability between runs (see Materials and Methods for details). (a) Relative area, (b) Cell number, (c) Adsorbed material, (d) Cell density within the colony, (e) Surface roughness *W*, (f) Coefficient of variation *CV*, (g) Relative maximum peak height *MaxP*1, and (h) Mean Fourier amplitude $I_{k}$. Evolution of morphological metrics relative to area increase: (i) Surface roughness *W*, (j) Coefficient of variation *CV*, (k) Relative maximum peak height *MaxP*1, and (l) Mean Fourier amplitude $I_{k}$.

The area increases faster for lower noise values (Fig 13). Cell numbers and adsorbed material are nearly identical across all systems. The cell density increases for larger levels of noise. The morphological metrics assume the highest values for orientation noise η=0.2. *W* and $I_{k}$ show bigger differences when normalized for area increase, indicating a higher sensitivity to differences in relative area increase (panels i, l). In contrast, *MaxP*1 only assumes values bigger than 0.1 for η=0.2, which has very regular fingers perpendicular to the colony center. Hence, this value is highly sensitive in detecting perpendicular expansion behaviour, independent of area increase. When normalizing for relative area increase, the colony with η=0.4 shows an early but less steep increase in all four metrics, indicating an early transition to finger growth but less pronounced fingers compared to the default parameters with η=0.2. The *CV* shows constant values for η=0.75, which is due to the slowly added, low number of grid cells to the colony, causing the grid anisotropy to become the dominant shaping factor. In summary, the orientation noise η has a huge impact on model behaviour, and colony development similar to *in vitro* colonies only occurs for a small range of parameter values.

#### Turning rate affects expansion speed.

**Fig 14:**
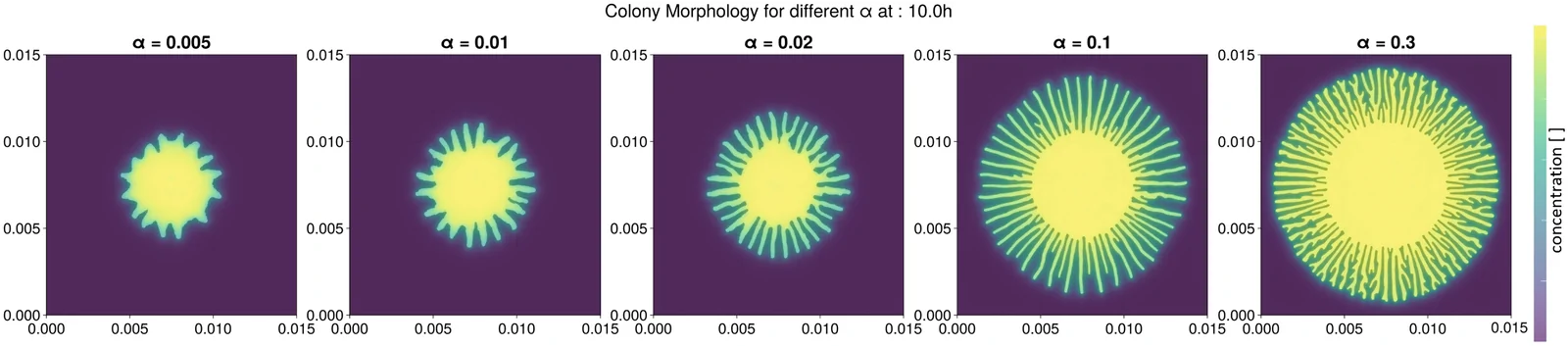
Colony morphology for five different values of the turning rate α after 10 h with default parameters (Table 1). For details of visualisation, see Materials and Methods.

**Fig 15:**
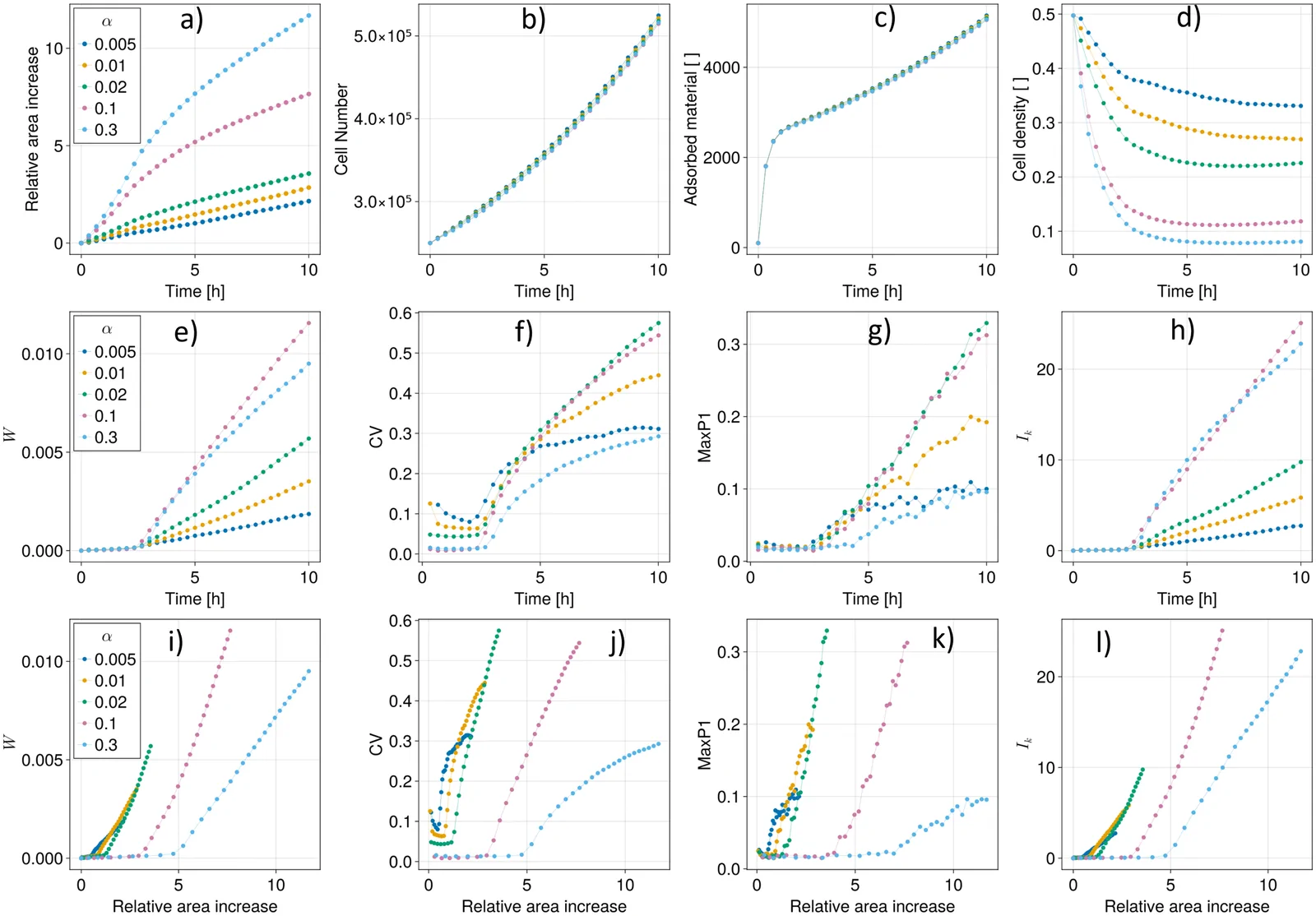
Temporal evolution of morphological metrics over 10 h for default parameters (Table 1) with five different turning rates in 20-minutes intervals. The results are for one simulation run each due to very low variability between runs (see Materials and Methods for details). (a) Relative area increase, (b) Cell number, (c) Adsorbed material, (d) Cell density within the colony, (e) Surface roughness *W*, (f) Coefficient of variation *CV*, (g) Relative maximum peak height *MaxP*1, and (h) Mean Fourier amplitude $I_{k}$. Evolution of morphological metrics relative to area increase: (i) Surface roughness *W*, (j) Coefficient of variation *CV*, (k) Relative maximum peak height *MaxP*1, and (l) Mean Fourier amplitude $I_{k}$.

The turning rate α determines how quickly an agent responds to changes in the surrounding chemical concentration and aligns its orientation parallel to the negative gradient. The two edge cases represent instantaneous alignment (α=1.0) versus no alignment (α=0.0). We varied α in 16 steps over a range of 0.0−1.0 and analysed five parameter values in that range that represent the different model behaviours observed (Fig 14). The area increases more rapidly for higher turning rates, while cell number and adsorbed material remain nearly constant across all systems (Fig 15). The cell density decreases as α is increased. This occurs because higher turning rates increase cellular sensitivity to local gradients, promoting outward movement. However, effective finger formation requires a balanced α value. For very high values (e.g., α=0.3), agents become overly sensitive to small gradient variations within fingers—such as between finger centres and edges—causing them to move perpendicular to finger axes and creating proliferations within fingers. Conversely, when α is very low, overall alignment towards the chemical gradient is weak, resulting in slower expansion and fewer, less pronounced and less regular fingers.

Analysis of the morphological metrics reveals a complex picture. The turning rate α=0.1 shows the highest values for *W* and $I_{k}$, slightly higher than α=0.3. This occurs because both metrics are sensitive to finger formation and area increase, with finger formation being stronger for α=0.1 but area increase being higher for α=0.3. The *CV* and *MaxP*1 metrics, which are less dependent on area increase and more sensitive to finger formation, show the highest values for α=0.02, followed closely by the default value α=0.1. When normalised for area increase, we observe that colonies with lower values of α grow fingers at a much lower area value than for α=0.1 and especially for α=0.3. However, for very low values such as α=0.005, finger formation becomes so irregular that *CV* and *MaxP*1 start to saturate after 5 h or a relative area increase of 1. We find that the duration of the circular expansion phase is independent of the value of α. The value of relative area increase for the onset of fingering, however, increases with increasing turning rate α.

**Fig 16:**
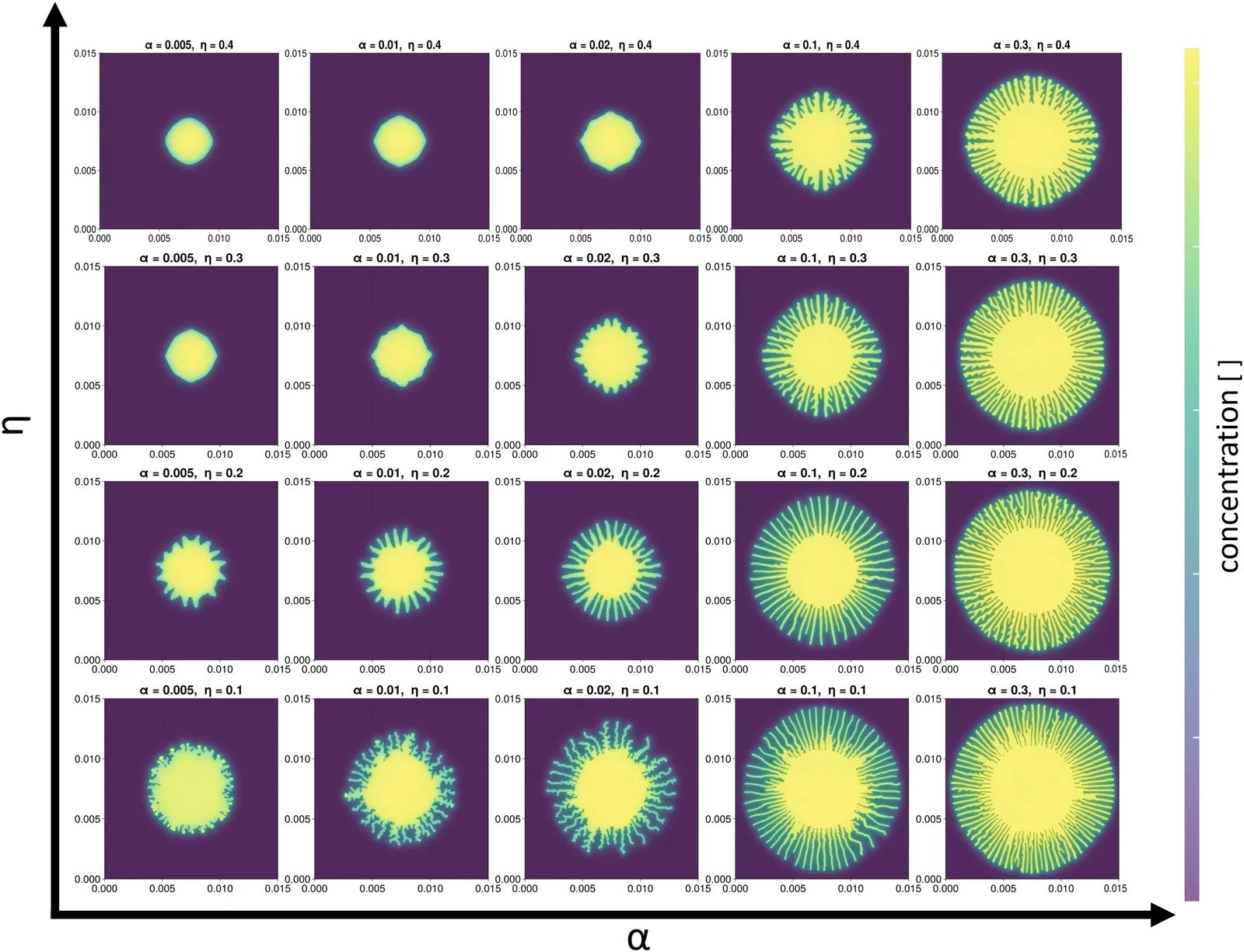
Colony morphology for five different values of the turning rate α (horizontal axis) and four different values of the noise $\eta_{0}$ (vertical axis) after 10 h with otherwise default parameters (Table 1). For details of visualisation see Materials and Methods.

Since both the noise η and the turning rate α influence the movement behaviour of agents, we examined their coupled effects across 20 parameter combinations that represent the different model behaviours observed in that range (Fig 16).

Within the chosen parameter range of η from 0.1 to 0.4 and α from 0.005 to 0.3, the turning rate α has a greater impact on colony morphology. The noise η exhibits an important effect: if it is too high, lattice anisotropy becomes a defining factor in the emerging morphology. However, η cannot be too low either, as this leads to more irregular finger formation, especially pronounced for low values of α. From visual inspection and metric evaluation, the parameter space where expansion behaviour is similar to experiments is quite small at η≈0.2 and α≈0.02−0.1, a range we have already analysed (Fig 15).

#### Grid and boundary properties affect expansion speed.

**Fig 17:**
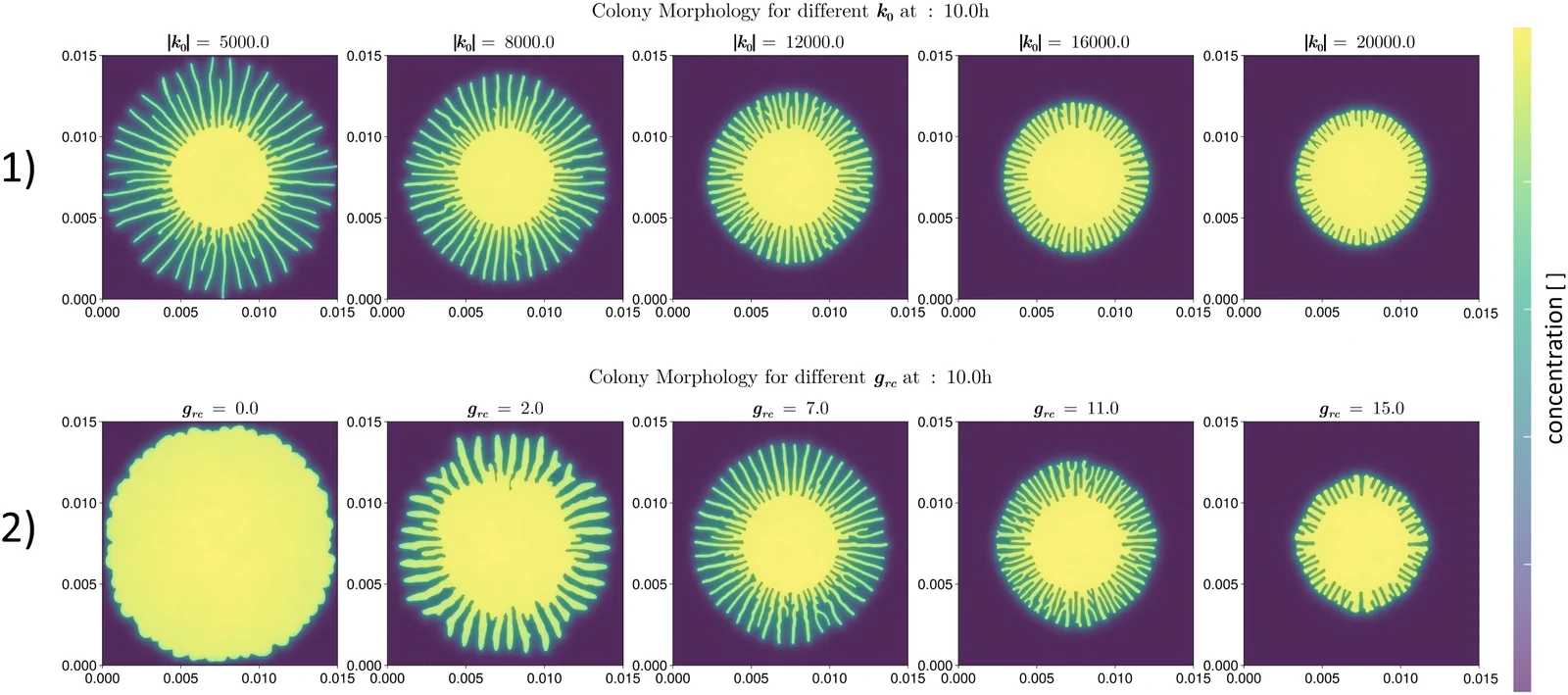
First row: Colony morphology for five different values of the grid strength |*k*_0_| after 10 h with default parameters (Table 1). Second row: Colony morphology for five different values of the grid recovery rate $g_{rc}$ after 10 h with default parameters (Table 1). For details of visualisation, see Materials and Methods.

After analysing the movement properties of the agents, we focused on the boundary properties in order to understand how such environmental factors impact colony expansion. We varied the grid strength *k*_0_ from −1000 to −25000 and the grid recovery rate $g_{rc}$ from 0.0 to 25.0 and analysed five parameter values each in those ranges that represent the different model behaviours observed (Fig 17). To make things more intuitive to understand, we always use the absolute value |*k*_0_| in the following sections to avoid confusion regarding the negative values for *k*_0_.

Both a lower absolute grid strength |*k*_0_| and a lower grid recovery rate $g_{rc}$ cause faster expansion. However, whilst $g_{rc}=0$ causes the expansion behaviour to change to circular growth, a lower |*k*_0_| does not appear to change the expansion pattern. A positive $g_{rc}$ value is required for finger formation, as it counteracts boundary removal from random collisions and instead removes boundaries only where directed, sustained movement occurs. This directed movement is strongest at emerging finger tips, where agents align strongly with the outward-pointing gradient, and weaker at finger bases, where weaker gradients produce more random movement. Consequently, $g_{rc}>0$ stabilises finger bases whilst allowing tips to advance, enabling finger formation. In contrast, *k*_0_ merely sets the total collision threshold for removal without constraining temporal distribution, thus influencing only the expansion timescale without affecting the qualitative pattern.

**Fig 18:**
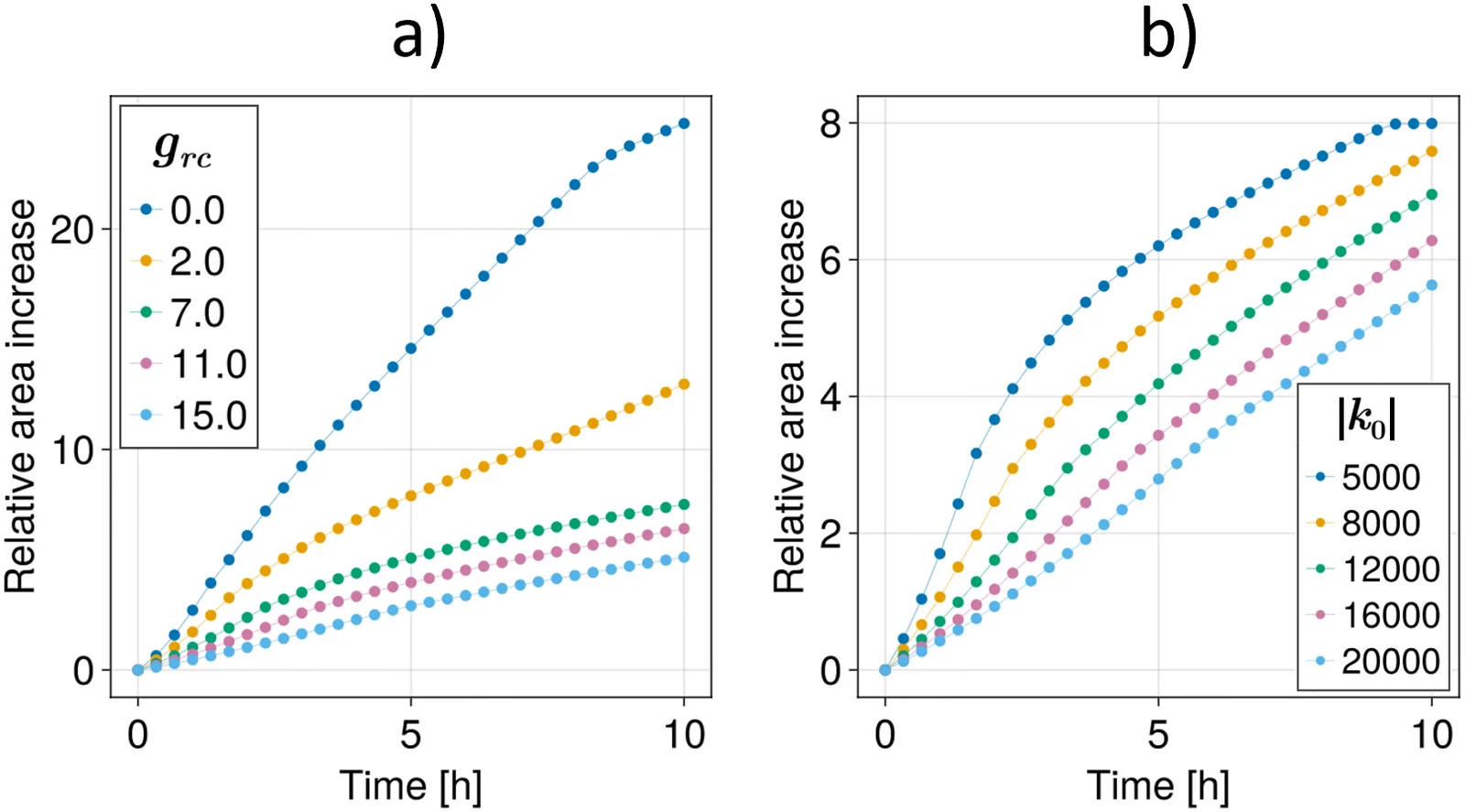
Temporal evolution of relative area increase over 10 h for default parameters (Table 1) in 20-minute intervals with (a) five different grid recovery rate values $g_{rc}$ and (b) five different absolute grid strength values |*k*_0_|. The results are for one simulation run each due to very low variability between runs (see Materials and Methods for details).

Similarly, low values of $g_{rc}$ of 0.0 and 2.0 show a much higher relative area increase (Fig 18), whereas all other changes in both parameters only steadily increase or decrease the relative area. Cell number and adsorbed material are not shown in the main manuscript (but can be seen in Section A.5 in S1 Appendix) as the behaviour is the same as for the default parameters. Cell density reflects the change in relative area.

**Fig 19:**
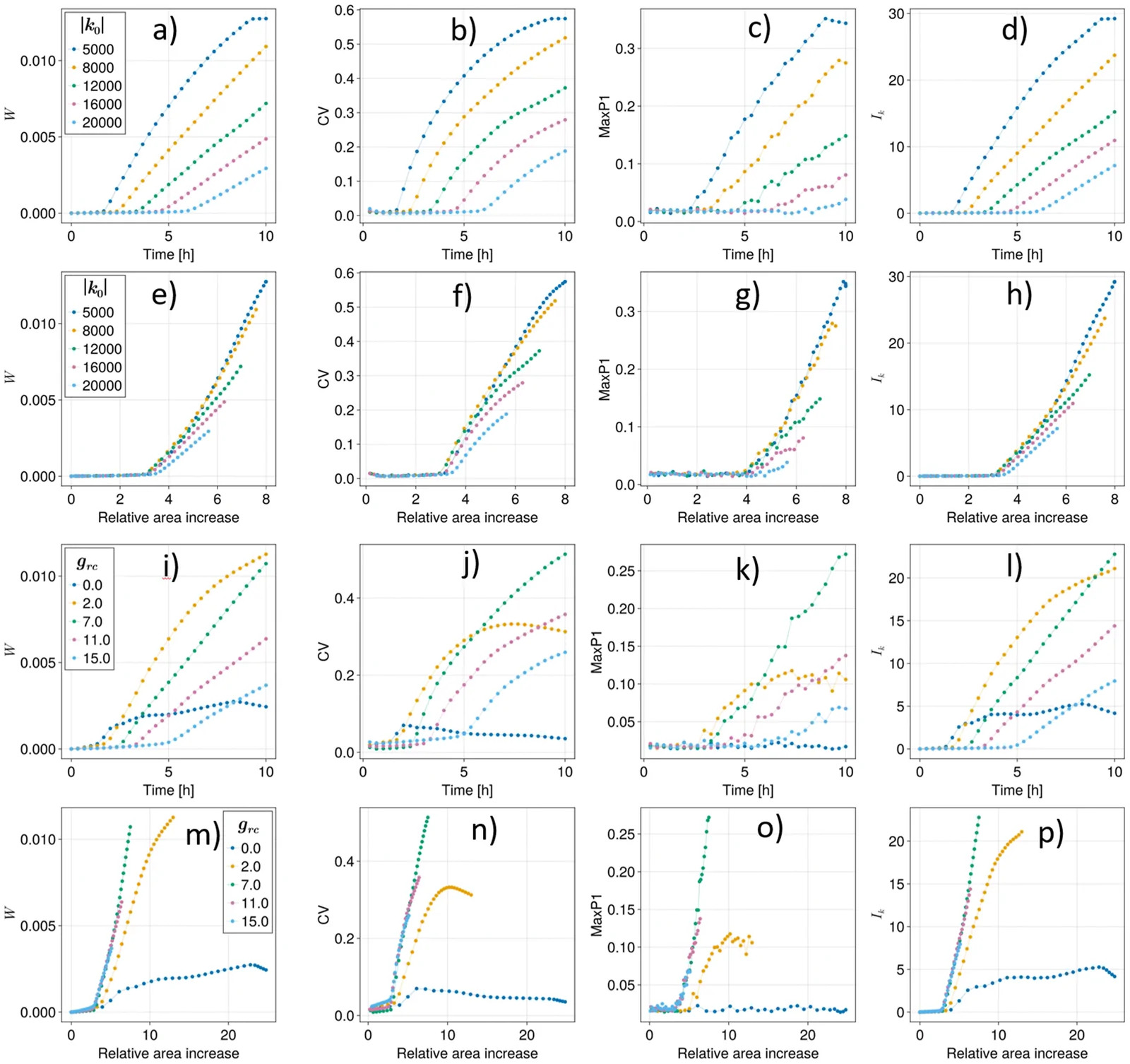
Evolution of morphological metrics over 10 h for default parameters (Table 1) in 20-minute intervals for different grid strength values |*k*_0_| relative to time (first row) and relative to area increase (second row); for different grid recovery rate values $g_{rc}$ relative to time (third row) and relative to area increase (fourth row). The results are for one simulation run each due to very low variability between runs (see Materials and Methods for details).

All morphological metrics relative to time for different values of |*k*_0_| show equidistant spacing with a later increase in values but similar slopes (Fig 19). When normalised by relative area increase, the trend becomes clearer. Except at very high grid strength values of |*k*_0_|, all morphological metrics exhibit a marked increase within the same relative area interval of three to four. Something very similar can be observed for the grid recovery rates $g_{rc}$ bigger than zero, where the time point for the onset of fingering increases with increasing $g_{rc}$. For the evolution with respect to relative area increase, all curves align except for $g_{rc}=0.0$ and to a lesser extend $g_{rc}=2.0$. We conclude that if $g_{rc}>0$, there is a wide parameter corridor for *k*_0_ and $g_{rc}$ that changes the temporal onset of fingering, i.e., the expansion speed, but not the nature of the expansion.

#### Diffusion coefficient affects colony morphology and expansion speed.

Following the previous results demonstrating that boundary properties have less influence on colony expansion behaviour than the movement properties of agents, we turned our attention to another major factor that influences agent movement: the diffusion of the chemotactically active substance. Various substances (pH/protons, glucose, cAMP, exosomes) [32–35] have been proposed as potential mediators of a chemotactic alignment in trypanosomes [33]. However, it remains unclear which specific substance could mediate the chemotactic alignment, as all are present in the experimental system.

The diffusion coefficients of these candidate substances at room temperature in water are well established in the literature, ranging from $9\times 10^{-9}$ m^2^/s (pH/*H*^+^), $6.5\times 10^{-10}$ m^2^/s (glucose) [58], $4\times 10^{-10}$ m^2^/s (cAMP) [59,60] to $7\times 10^{-12}-1.5\times 10^{-12}$ m^2^/s (exosomes, depending on size) [32,61–63]. Although the experimental setup of trypanosome colonies involves a complex liquid medium that consists of multiple components [4] placed on top of an agarose gel, previous studies have shown that the diffusion coefficients of various materials in such media differ only slightly from those in pure water [13,64]. Therefore, we can reasonably use the diffusion coefficients measured in water as reference points.

**Fig 20:**
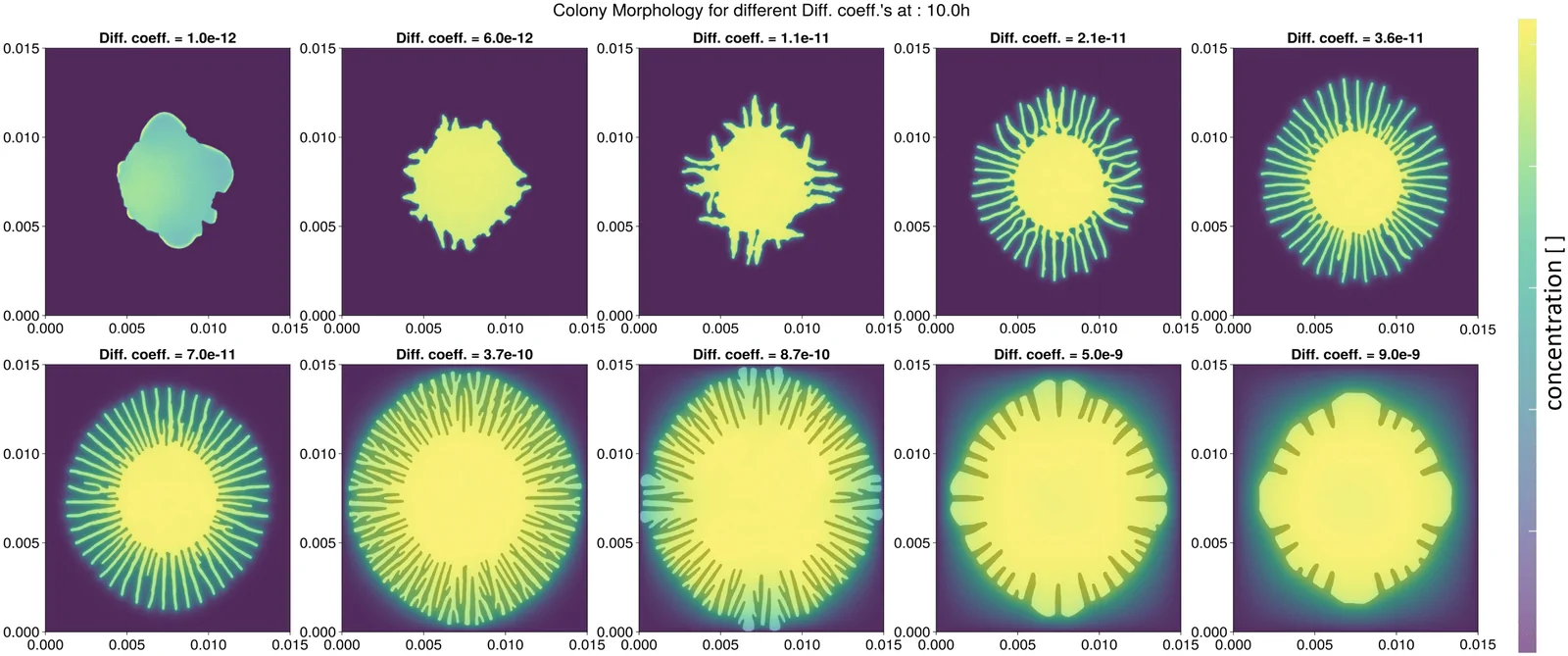
Colony morphology for ten different values of the diffusion coefficient $D_{s}$ after 10 h with default parameters (Table 1). For details of visualisation see Materials and Methods.

We varied the diffusion coefficient $D_{s}$ in our simulations across the range of biologically relevant values and studied its influence on system behaviour (Fig 20). The diffusion coefficient has a tremendous influence on the expansion behaviour. For the lowest value of $D_{s}=1\times 10^{-12}$ m^2^/s, diffusion is so slow that no colony-wide concentration gradient decreasing from the center to the boundaries is established within 10 h. Instead the highest concentration is found at the boundaries of the colony. The resulting colony morphology is anisotropic and does not exhibit a finger-like pattern. For higher diffusion coefficients, a colony-wide gradient can be established, with the highest concentration in the central part of the colony decreasing outward. This gradient becomes more and more pronounced the higher the diffusion coefficient $D_{s}$ becomes. The resulting morphologies change steadily toward the very regular finger-like patterns observed at $D_{s}=7\times 10^{-11}$ m^2^/s. For higher values up to $8.7\times 10^{-10}$ m^2^/s, expansion becomes even faster, but fingers start to branch more and also merge, forming a more circular and less fractal expansion front. For even higher values of $5\times 10^{-9}$ m^2^/s and beyond, expansion becomes slower again. Thick protrusions emerge that are not comparable to the finger-like patterns observed in experiments. Starting at $D_{s}=3.7\times 10^{-10}$ m^2^/s, the adsorbed chemical diffuses significantly beyond the colony fingers, which can be seen in the changing colours outside of the colony boundaries (Fig 20), a trend that increases for larger $D_{s}$ values. At $D_{s}=9\times 10^{-9}$ m^2^/s, the chemical and its decreasing concentration gradient reach far beyond the colony boundaries.

**Fig 21:**
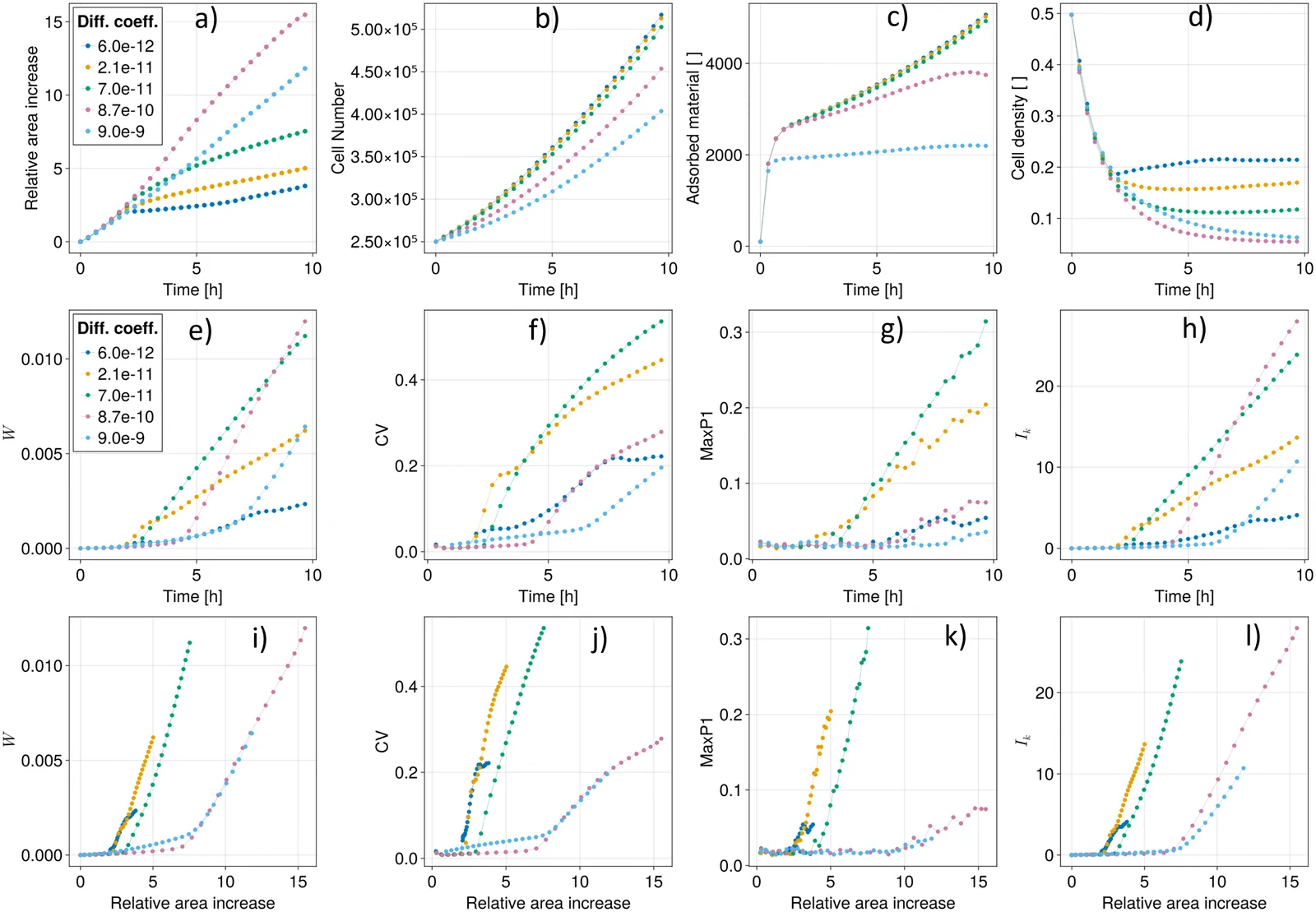
Temporal evolution of morphological metrics over 10 h for default parameters (Table 1) with five representative diffusion coefficient $D_{s}$ values in 20-minute intervals. The results are for one simulation run each due to very low variability between runs (see Materials and Methods for details). (a) Relative area increase, (b) Cell number, (c) Total amount of material in the gradient grid, (d) Cell density within the colony, (e) Surface roughness *W*, (f) Coefficient of variation *CV*, (g) Relative maximum peak height *MaxP*1, and (h) Mean Fourier amplitude $I_{k}$. Evolution of morphological metrics relative to area increase: (i) Surface roughness *W*, (j) Coefficient of variation *CV*, (k) Relative maximum peak height *MaxP*1, and (l) Mean Fourier amplitude $I_{k}$. For details of visualisation see Materials and Methods.

The colony behaviour is the same for the first two hours (Fig 21). After that, the area increases faster for higher diffusion coefficients until $D_{s}=8.7\times 10^{-10}$ m^2^/s. Then, the area increases more slowly for higher values of $D_{s}$. The cell number and adsorbed material are similar for all values of $D_{s}$ until $D_{s}=7.0\times 10^{-11}$ m^2^/s and then decrease with increasing $D_{s}$. The cell density mirrors the relative area increase.

Analysis of the morphological metrics reveals a complex picture. The default diffusion coefficient $D_{s}=7.0\times 10^{-11}$ m^2^/s shows the highest values for *CV* and *MaxP*1, slightly higher than $D_{s}=2.1\times 10^{-11}$ m^2^/s. This occurs because both metrics are sensitive to finger formation independent of area increase, with finger formation being slightly stronger for $D_{s}=2.1\times 10^{-11}$ m^2^/s. This can be seen in *W* and $I_{k}$, which are both sensitive to finger formation and area increase, showing the highest values for $D_{s}=8.7\times 10^{-10}$ m^2^/s, slightly higher than for $D_{s}=2.1\times 10^{-11}$ m^2^/s, as finger formation is stronger in the latter, whilst area increase is stronger in the former. Additionally, a slight preferential growth along the cardinal lattice axes (x and y directions) can be observed in some parameter regimes. This is an effect of a slight lattice anisotropy remaining. See section A.6 in S1 Appendix for more details. When normalised for area increase, all metrics behave very similarly. We observe that colonies with lower values of $D_{s}$ start forming fingers at a lower relative area. However, for the low value of $D_{s}=6.0\times 10^{-12}$ m^2^/s, all morphological metrics also indicate a beginning saturation in finger formation after a relative area increase of approximately 3. The system with default diffusion coefficient $D_{s}=7.0\times 10^{-11}$ m^2^/s shows a later onset of non-circular expansion but a similar increase in the metrics, indicating similar rapid expansion in finger-like patterns. For higher values of $D_{s}$, all metrics show a later onset of increase and a slower slope, indicating a less finger-dominated expansion pattern. In summary, the diffusion coefficient has a clear effect on the onset of fingering with respect to relative area increase and on finger morphology.

Our parameter sensitivity analysis shows that the different parameters analysed exhibit distinct effects on model behaviour. Movement parameters η and α demonstrate a narrow parameter window for producing behaviour similar to experiments. Boundary property parameters *k*_0_ and $g_{rc}$ primarily affect expansion speed with minimal influence on expansion pattern. The diffusion coefficient $D_{s}$ exerts a substantial influence on expansion dynamics. Low values result in slow, irregular colony expansion. Intermediate values enhance both expansion speed and regularity. For high values, this trend reverses such that the colonies expand more slowly and regularly but exhibit progressively reduced finger formation.

## Discussion

Our agent-based model demonstrates that complex colony-level patterns, such as those observed in trypanosome social motility, can emerge in a simplified system consisting only of single-cell motility, cell proliferation, interactions with colony boundaries, and negative autochemotaxis.

### Model parameters

#### Parameter hierarchy and pattern formation.

Model parameters play fundamentally different mechanistic roles. The interplay between diffusion coefficient $D_{s}$, turning rate α, and orientation noise η determines *whether* finger-like growth occurs, whilst doubling time $\tau_{d}$, grid strength *k*_0_, and grid recovery rate $g_{rc}$ affect mainly *how fast* fingers are forming.

#### Doubling time.

The two simulated doubling times demonstrate a non-linear relationship between cell growth and area increase. Newly added cells through division appear to accelerate growth, but much more slowly than the population grows, indicating that some limiting factor is at play. Relative area growth appears to be primarily driven by agents at the colony surface, where density appears similar across both systems. This requires further investigation by comparing with experimental data using cell lines with different doubling times [3,4,34] as well as detailed analyses of agent trajectories in the simulations.

#### Boundary mechanics.

The boundary parameters grid strength *k*_0_ and grid recovery rate $g_{rc}$ exhibit a wide ’parameter corridor’ where changes affect only expansion speed without altering the fundamental nature of colony morphologies. This robustness suggests that the specific mechanical properties of the colony-agarose interface may be less critical than previously thought [4]. However, our current boundary implementation is quite simplistic, and if certain properties are measured, it could be expanded.

#### Orientation noise and turning rate.

The orientation noise η and turning rate α demonstrate a narrow parameter window where finger formation is most pronounced, regular, and most similar to experiments. Such behavior represents a remarkable result. It suggests that finger formation emerges directly from the specific properties of the directional random walk of individual agents. Our results coincide with previous experimental findings where modifying flagellum activity and motility properties of individual trypanosomes can suppress social motility [1,3].

#### Diffusion and chemical signalling.

The diffusion coefficient $D_{s}$ affects both the speed and pattern of colony expansion. For low values of $D_{s}=1.0-6\times 10^{-12}$ m^2^/s, finger formation is absent and colony growth is substantially reduced, likely because information cannot spread sufficiently rapidly across the colony to establish a global chemical gradient pointing outward toward the boundaries. Conversely, very high diffusion coefficients lead to more circular rather than finger-like growth. At these high values, signals diffuse rapidly and concentration gradients become shallow but far-reaching, extending beyond the colony boundaries. Such conditions inhibit finger formation because the highest gradient is no longer localised at the finger tips, where it guides directional growth, but instead decreases monotonically with distance from the colony centre, resulting in a unified expansion front without finger formation.

**Fig 22:**
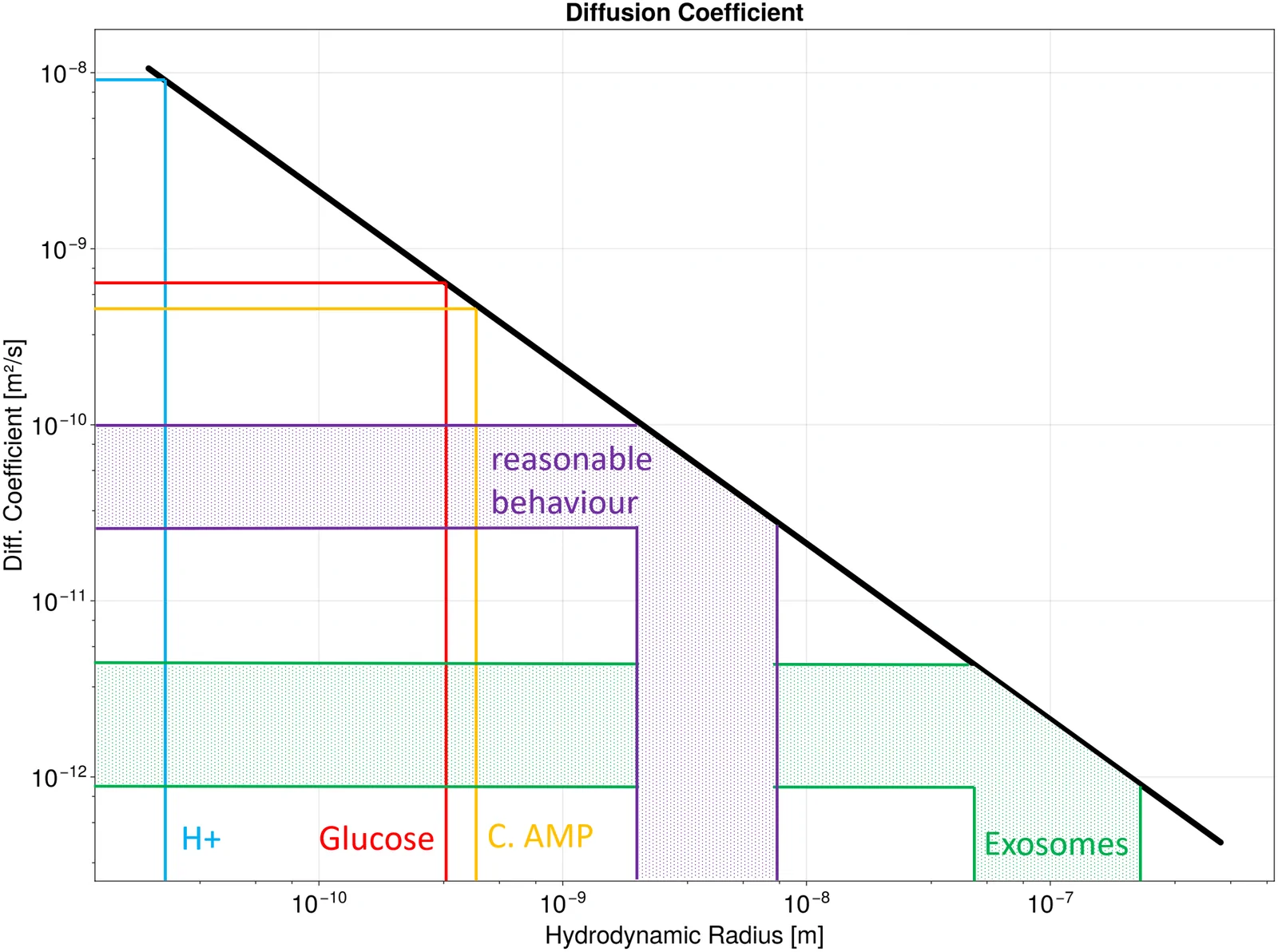
Diffusion coefficient as a function of hydrodynamic radius of diffusion particles in water according to the Einstein-Stokes equation. Model behavior similar to experiments occurs at diffusion coefficients between $2\times 10^{-11}$ and 10^-10^ m^2^/s, corresponding to hydrodynamic radii between 2–10 nm.

The range of simulated diffusion coefficients (10^−12^ to 10^−9^ m^2^/s) spans the known values for several proposed signalling agents, including pH (H^+^), cAMP, glucose, and small exosomes [3,32,34]. However, the model’s behavior most similar to experiments occurs within the range of $D_{s}=2\times 10^{-11}$ to 10^−10^ m^2^/s. Using the Einstein-Stokes equation


$$\begin{array}{c} D_{s}=\frac{k_{B}T}{6\pi \eta r}, \end{array}$$ (9)

where $k_{B}$ is the Boltzmann constant, *T* = 293.15 K is the absolute temperature, and η=0.001 kg/(m·s) is the dynamic viscosity of water at *T*, the corresponding hydrodynamic radius *r* of the molecule can be estimated to be between 2–10 nm (Fig 22).

Using established conversion estimations for molecular weights [65], such radii correspond to small proteins with molecular weights between 12.1–1690 kDa. Therefore, our results suggest searching for signaling proteins, macromolecules, or lipids in that weight range. It should be noted that this range of molecular weights and diffusion coefficients could be slightly shifted towards higher molecular weights or, respectively, lower diffusion coefficients, as the real colonies grow slower compared to our simulations, possibly allowing for slower diffusion.

### Model design choices

The present model establishes a robust foundation for understanding trypanosome social motility, with several aspects simplified to enable efficient computation and focused validation against existing experimental data. Each represents a model limitation but also an opportunity for future elaboration.

#### Point-like agent representation.

Our model treats trypanosomes as point agents, whereas individual cells possess elongated rod-like shapes with considerable aspect ratios roughly between 8 and 12 [27,28]. Cell shape directly relates to orientation, as trypanosomes swim by pulling themselves forward with their flagellum. Our volume-exclusion rule (one agent per grid cell) differs from the more complex excluded-volume constraints that rod-shaped agents would impose, changing their movement properties in crowded environments [66], which in turn could affect expansion rates and patterning.

#### Hydrodynamic interactions.

Abstracting the complex hydrodynamic swimming of trypanosomes [27,28,42] to dry active matter [41] enabled tractable simulation times but omits fluid-mediated interactions that would modify the agent movement equations [67] and could therefore also influence the pattern formation.

#### Boundary mechanics.

More sophisticated hydrodynamic boundary implementations have been demonstrated for bacterial systems [21,23,47] on smaller scales, but would require measurements of physical parameters (viscosity, surface tension, lubrication properties,...) not yet performed for *Trypanosoma brucei* colonies, as well as substantial computational resources. These would behave in a more complex way than our phenomenological boundary implementations and could therefore have a greater influence on pattern formation.

#### Nutrient availability.

The model assumes abundant, non-depleting nutrients. Nutrient depletion would reduce proliferation rates based on agents’ local environment, which could affect finger morphologies, as newly expanded areas at the finger tips would have higher nutrient concentrations. Future experimental manipulation of nutrient availability and cell proliferation rates during colony expansion could clarify whether metabolic constraints are a factor in real *Trypanosoma brucei* colonies, as observed in some bacterial systems [13,68], and therefore should be incorporated.

#### Heterogeneous conditions.

The resistance of the environment towards colony expansion is modeled by grid strength *k*_0_ and grid recovery rate $g_{rc}$, which are homogeneous throughout the simulation space. Agarose substrates in experiments invariably contain small defects or inhomogeneities that could locally change this resistance. Future experiments could specifically investigate whether this significantly affects colony pattern formation by mechanically modifying substrate properties on one side of a colony to determine how strongly and in which manner this influences expansion behavior. Such findings could then be validated and incorporated into the model by locally changing *k*_0_ and $g_{rc}$.

#### Chirality.

Previous studies have shown that social motility can also occur with chiral fingers [32,33]—a behaviour that is currently not captured by our model. Addressing this phenomenon would require formulating hypotheses about the underlying mechanisms that could alter local motility, such as asymmetric propulsion [69], and subsequently incorporating these processes into the model.

### Future directions

Based and beyond these considerations, several promising research directions emerge:

#### Computational optimisation.

Outsourcing the computationally limiting parts of the model from CPUs to GPUs could increase performance by an order of magnitude [70,71], enabling us to include more mechanistic details into the simulations (e.g., rod shape, hydrodynamics, boundaries,...).

#### Simulation-based inference.

Sensitivity analysis has inherent limitations: it is very difficult to explore parameter interactions and compensation effects (e.g., whether weak boundaries with fast diffusion can substitute for strong boundaries with slow diffusion), and exploring high-dimensional spaces (more than five parameters) through manual sweeps becomes computationally intractable and difficult to interpret intuitively. Once computational performance improves, formal parameter inference methods [72,73] such as approximate Bayesian computation (ABC) [74,75] or neural likelihood estimation [76] could systematically address these limitations. Such methods iteratively simulate with stochastically selected parameter combinations, compare outputs to experimental data using our metrics, and algorithmically refine parameter distributions; such methods have already been successfully applied to other agent-based models [77,78] in systems biology.

#### Trajectory analysis.

In addition to colony morphology, extracting agent trajectory data and comparing it with experimental single-cell tracking [4,33] from social motility assays would provide additional validation opportunities for our model results and the ability to test specific hypotheses, such as whether faster-growing colonies exhibit stronger boundary-directed movement.

#### Generalisation.

The framework could extend to other microorganisms. *Pseudomonas aeruginosa* [23,79], for example, exhibits many similar properties: microswimmers in colonies that form characteristic patterns, which could be modelled with our framework. However, organism-specific parameters (agent size, rhamnolipid secretion, etc.) would require model modifications and experimental measurements before quantitative predictions become possible.

## Conclusion

Our model reproduces the patterning characteristics of social motility of single colonies and demonstrates that individual movement properties of trypanosomes are critical for the emergence of finger-like patterns. Our main prediction is that boundary interactions have to be coupled with negative auto-chemotaxis and that previously proposed auto-chemotactically active substances cannot be directly responsible for social motility and likely represent correlation rather than causation. To advance this research, we have provided several testable hypotheses for experimental validation, including the motility properties of trypanosomes, the size of potential signalling agents, and the robustness with respect to mechanical boundary properties.

## Supporting information

S1 Appendix A.1: Area of Cells. A.2: Von Neumann Stability Analysis. A.3: Experimental Methods and Quantification from Previous Work. A.4: Default Parameter Values. A.5: Further metrics for grid and boundary properties. A.6: Lattice Anisotropy. (PDF)
