# Supplementary material for: An agent-based model of Trypanosoma brucei social motility to explore determinants of colony pattern formation
Source: PLoS Comput Biol. 2026 Aug 12;22(8):e1013698. doi: 10.1371/journal.pcbi.1013698 (PMC13489481; doi:10.1371/journal.pcbi.1013698)
Supplement: Supplementary file 1 [file pcbi.1013698.s001.pdf]

Supporting information to "An agent-based model of Trypanosoma brucei social motility to explore determinants of colony pattern formation"

Andreas Kuhn[1], Timothy Krüger[2], Markus Engstler[2], Sabine C. Fischer[1*]

1 Julius-Maximilians-Universität Würzburg, Biocenter, Chair for Computational and Theoretical Biology (CCTB), Klara-Oppenheimer-Weg 32, 97074 Würzburg, Germany
2 Julius-Maximilians-Universität Würzburg, Biocenter, Department of Cell and Developmental Biology, Am Hubland, 97074 Würzburg, Germany

* sabine.fischer@uni-wuerzburg.de

A.1 Area of Cells

The projected two-dimensional area of trypanosomes during a social motility assay was determined using scanning electron microscope images from [1]. The cell body was manually segmented with Fiji, and the pixel count was used to calculate an approximate area of 18.0 μm^2.

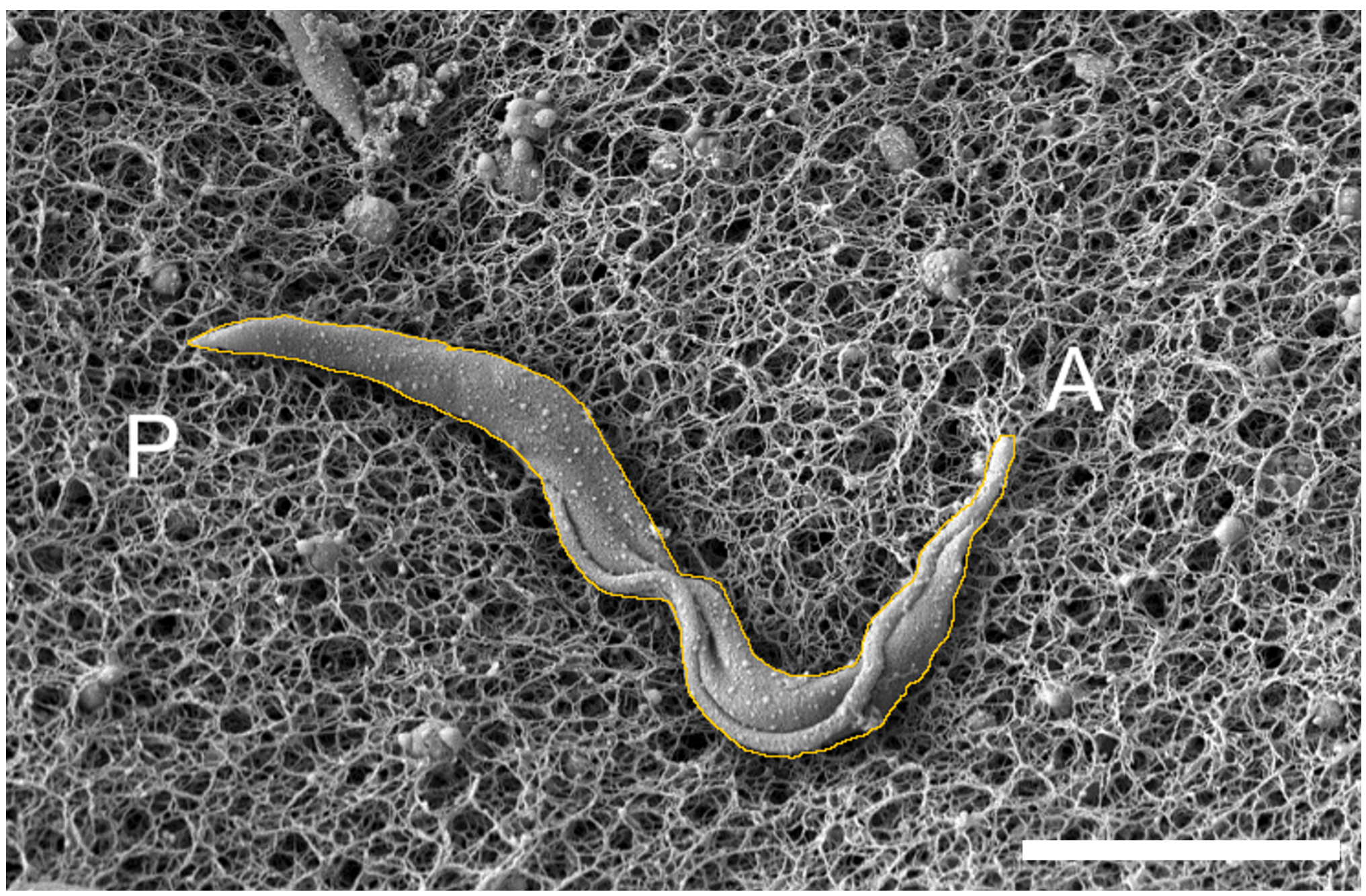

Fig A.1. Scanning electron micrograph of a single trypanosome fixed while swimming on the surface of an agarose gel. The manually determined outline of the cell body is shown in orange. A and P indicate the anterior and posterior ends, respectively. Scale bar: 5 μm. Image adapted from [1].

A.2 Von Neumann Stability Analysis

The von Neumann stability analysis for standard diffusion equations solved with finite difference schemes has been extensively documented in the literature [2–4]. Here, we focus specifically on the stability implications when extending the analysis to our modified diffusion equation that includes additional decay and adsorption terms

$$\frac{\partial s(x,t)}{\partial t} = D_s \nabla^2 s(x,t) - d_r s(x,t) + \frac{a_d}{s_f^2} \sum_{n=1}^{N} \delta(x - x_n), \tag{1}$$

where D_s is the diffusion coefficient, d_r represents the decay rate, s_f is the scaling factor between the ABM grid and gradient grid, a_d is the adsorption rate caused by the N agents at their ABM grid cells x_n, δ is the Dirac delta function.

For stability analysis, we consider the worst-case scenario where all ABM grid points are occupied by agents. In this case, the adsorption term $\frac{a_d}{s_f^2} \sum_{n=1}^{N} \delta(x - x_n)$ becomes a constant $\frac{s_f^2}{s_f^2} a_d = a_d = C$ at every gradient grid point. The equation can be written in the form

$$\frac{\partial s(x,t)}{\partial t} - D_s \nabla^2 s(x,t) + d_r s(x,t) = C. \tag{2}$$

Although this equation contains a non-homogeneous term C, the stability of the numerical scheme depends only on the homogeneous part of the equation. This can be seen by examining the round-off error $\epsilon_{i,j}^n$, which is the difference between the numerical solution $N_{i,j}^n$ and the solution $u_{i,j}^n$ with infinite machine precision

$$\epsilon_{i,j}^n = N_{i,j}^n - u_{i,j}^n. \tag{3}$$

Both solutions must satisfy our discretized equation

$$\begin{aligned} s_{i,j}^{n+1} = s_{i,j}^n + D_s \Delta t \left(\frac{s_{i+1,j}^n - 2s_{i,j}^n + s_{i-1,j}^n}{\Delta x^2} + \frac{s_{i,j+1}^n - 2s_{i,j}^n + s_{i,j-1}^n}{\Delta y^2} \right). \\ -d_r \Delta t \, s_{i,j}^n + C\Delta t \end{aligned} \tag{4}$$

If we let L^* represent our discretized operator, then for $u_{i,j}^n$

$$L^*(u_{i,j}^n) = \tag{5}$$

$$-\frac{u_{i,j}^{n+1} - u_{i,j}^n}{\Delta t} + D_s \left(\frac{u_{i+1,j}^n - 2u_{i,j}^n + u_{i-1,j}^n}{\Delta x^2} + \frac{u_{i,j+1}^n - 2u_{i,j}^n + u_{i,j-1}^n}{\Delta y^2} \right) - d_r u_{i,j}^n \tag{6}$$

$$= -C\Delta t \tag{7}$$

And similarly for the numerical solution

$$L^*(N_{i,j}^n) = -C\Delta t. \tag{8}$$

By the linearity of the operator L^*, the round-off error satisfies

$$L^*(\epsilon_{i,j}^n) = L^*(N_{i,j}^n) - L^*(u_{i,j}^n) = -C\Delta t + C\Delta t = 0. \tag{9}$$

Therefore, the round-off error $\epsilon_{i,j}^n$ satisfies the homogeneous equation, allowing us to apply the standard von Neumann stability analysis for the explicit forward time-centered space scheme.

The derivation follows the same steps as in [2], but extended to 2D (see [5] for the general 2D case) and includes an additional decay term. Therefore, we can write $\epsilon_{i,j}^n$ as a Fourier series

$$\epsilon_{i,j}^n = E_m(t)e^{ik_1x+ik_2y}, \tag{10}$$

$$\epsilon_{i\pm1,j}^n = E_m(t)e^{ik_x(x\pm\Delta x)+ik_yy}, \tag{11}$$

$$\epsilon_{i,j\pm1}^n = E_m(t)e^{ik_xx+ik_y(y\pm\Delta y)}, \tag{12}$$

$$\epsilon_{i,j}^{n+1} = \epsilon_{i,j}^n + D_s\Delta t\left(\frac{\epsilon_{i+1,j}^n - 2\epsilon_{i,j}^n + \epsilon_{i-1,j}^n}{\Delta x^2} + \frac{\epsilon_{i,j+1}^n - 2\epsilon_{i,j}^n + \epsilon_{i,j-1}^n}{\Delta y^2}\right) - d_r\Delta t\epsilon_{i,j}^n. \tag{13}$$

We examine the amplification factor $G = \left|\frac{\epsilon_{i,j}^{n+1}}{\epsilon_{i,j}^n}\right|$ which represents the relative change of error between iterations

$$G = 1 + D_s\Delta t\left(\frac{e^{ik_x\Delta x} + e^{-ik_x\Delta x} - 2}{\Delta x^2} + \frac{e^{ik_y\Delta y} + e^{-ik_y\Delta y} - 2}{\Delta y^2}\right) - d_r\Delta t. \tag{14}$$

Using the identity $e^{ik\Delta x} + e^{-ik\Delta x} = 2\cos(k\Delta x)$, this can be rearranged to

$$G = 1 + 2D_s\Delta t\left(\frac{\cos(k_x\Delta x) - 1}{\Delta x^2} + \frac{\cos(k_y\Delta y) - 1}{\Delta y^2}\right) - d_r\Delta t. \tag{15}$$

Since $\cos(\theta) - 1 \leq 0$ for all θ, the most restrictive condition occurs when $\cos(k_x\Delta x) = \cos(k_y\Delta y) = -1$ [5], giving

$$G = 1 - 4D_s\Delta t\left(\frac{1}{\Delta x^2} + \frac{1}{\Delta y^2}\right) - d_r\Delta t. \tag{16}$$

For stability, we require $|G| \leq 1$, which leads to

$$-1 \leq 1 - 4D_s\Delta t\left(\frac{1}{\Delta x^2} + \frac{1}{\Delta y^2}\right) - d_r\Delta t \leq 1. \tag{17}$$

The right inequality is always satisfied, and from the left inequality, we get

$$-1 \leq 1 - 4D_s\Delta t\left(\frac{1}{\Delta x^2} + \frac{1}{\Delta y^2}\right) - d_r\Delta t. \tag{18}$$

Which simplifies to:

$$4D_s\Delta t\left(\frac{1}{\Delta x^2} + \frac{1}{\Delta y^2}\right) + d_r\Delta t \leq 2. \tag{19}$$

As in our simulation setup $\Delta x = \Delta y$, this can be further simplified and rearranged to our final stability condition on Δt:

$$\Delta t \leq \frac{\Delta x^2}{4D_s + \frac{d_r\Delta x^2}{2}}. \tag{20}$$

For the standard diffusion equation (when $d_r = 0$), this reduces to the familiar condition $\Delta t \leq \frac{\Delta x^2}{4D_s}$ [5]. Importantly, the presence of the decay term d_r further restricts the maximum allowable time step, requiring an even smaller Δt to maintain numerical stability in our simulations.

A.3 Experimental Methods and Quantification from Previous Work

The experimental data and quantification methods used to validate our model are based on our previous work [6], which established quantitative metrics for characterising *Trypanosoma brucei* social motility at the colony level. We provide a brief summary here and refer readers to the original publication for complete methodological details.

Cell Culture and Motility Assays

Experiments were conducted using the pleomorphic AnTat 1.1 *Trypanosoma brucei* cell line grown in SDM-79-glc medium supplemented with 10% foetal bovine serum. To enable fluorescence imaging whilst maintaining natural colony behaviour, 20% fluorescently labelled cells (expressing tdTomato) were mixed with 80% wild-type cells before each experiment. Trypanosomes were concentrated to a density of 4×10^8 cells/ml, and 2 μl droplets were deposited onto agarose gel surfaces. This concentration was calculated to yield approximately 10^6 cells spread in a monolayer covering 30 mm^2, with a final concentration of 1.7×10^{10} cells/ml in the absorbed droplet volume of 0.06 μl after the culture medium was absorbed by the hydrogel.

Agarose gels were prepared by pouring 1 ml of SDM-79-glc medium containing 0.4% agarose into 35 mm Petri dishes, allowing gelation for 1 h with closed lids, then drying for 30 min under a laminar flow hood. The inherent variability in the pleomorphic AnTat 1.1 cell line naturally produced both SoMo-positive (actively migrating) and SoMo-negative (non-migrating) colonies on the same gel. Colonies were imaged over 48 h using an iBright CL1000 fluorescence imager (Thermo Fisher Scientific), capturing time-series data at regular intervals.

For the assay in Fig.1, gellan gum gels ($0, 2\%$ Gelrite, Serva) prepared in Cunningham´s medium [7] without sugars and $10mM$ glycerol were used, which resulted in the migration of more numerous and thinner fingers, but similar migration dynamics compared to agarose gel assays.

Image Analysis and Morphological Quantification

Fluorescence images were processed through a semi-automated pipeline to segment colonies from background and align them across time series. Segmentation was performed using the machine learning-based Trainable Weka Segmentation plugin in Fiji, producing binary images where pixels with value 1 belong to the colony and pixels with value 0 represent background. Alignment and stacking across time points were achieved using the StackReg plugin (Rigid Body transformation).

Colony morphology was quantified using two complementary metrics originally developed for yeast colonies [8] and adapted for the Trypanosoma system. The angular metric $S_\theta(i)$ measures radial variations by dividing colonies into $N = 360$ circular sectors centred on the initial colony centroid and counting pixels in each sector. The pair correlation metric $S_\sigma(i)$ quantifies deviations from circular symmetry by measuring angles between pixel pairs (using $N_p = 180$ angular bins) relative to the colony centroid. In finger-like colonies, the first peak in $S_\sigma(i)$ represents pixel pairs along the same finger, whilst subsequent peaks indicate spacing between neighbouring fingers.

From these raw metrics, four derived measures characterise colony morphology: (1) surface roughness W, measuring overall boundary irregularity; (2) coefficient of variation CV of the angular metric, quantifying radial fluctuations; (3) relative maximum peak height $MaxP1$ from the pair correlation metric, indicating finger prominence; and (4) mean Fourier amplitude I_k from the transformed angular metric,

quantifying periodic surface fluctuations. Together, these metrics distinguish circular from finger-like growth and track the transition between growth phases.

A.4 Default Parameter Values

Here we provide a more detailed explanation of why certain default parameter values (see Table 1) were chosen:

R_t was chosen to be as small as possible without introducing artefacts. If R_t becomes too small, the discrete nature of the grid can create ambiguous cases where no unique tangent can be determined, causing agents to become trapped in unrealistic scenarios.

For R_c, we conducted extensive parameter sweeps and found that it must exceed a threshold value (approximately $R_c > 5$, though this depends on other parameters, primarily k and g_{rc}) to allow multiple collision effects to compound. Below this threshold, the boundary develops fractal characteristics (particularly at low g_{rc} values), which contradicts experimental observations where surface tension maintains smooth boundaries.

s_f was chosen to be 4 because larger values made the gradient grid very coarse, which changed the expansion behaviour and caused the expanding fingers to be mapped to individual gradient grid points. Conversely, smaller values did not change the system behaviour but increased computation time significantly. Of course, this also depends on other parameters, especially Δx and L, and could require adjustment if these are changed from their default values.

A.4.1 Variations between Simulations

The variation between simulation runs is very small, as demonstrated in Fig. A.2, where the model was simulated five times with identical parameters and the resulting colony morphology after 10 h is displayed. By eye, there is hardly any difference to be spotted. This is confirmed by the calculated quantities in Fig. A.3, where the mean of these five runs together with their standard deviation is shown. The deviation between runs for relative area, cell number, adsorbed material, and cell density is so small that the error bars of the standard deviation are barely visible. The same is true for the metrics surface roughness W, coefficient of variation CV, relative maximum peak height $MaxP1$, and mean Fourier amplitude I_k for simulation times shorter than 5 h, or equivalently, a relative area increase below 5. Beyond this point, the metrics show small differences, with $MaxP1$ exhibiting the largest deviation as it is most sensitive to non-uniform finger distributions. Nevertheless, the deviations remain sufficiently small to justify running each parameter set only once, as results with similar parameters show minimal variation.

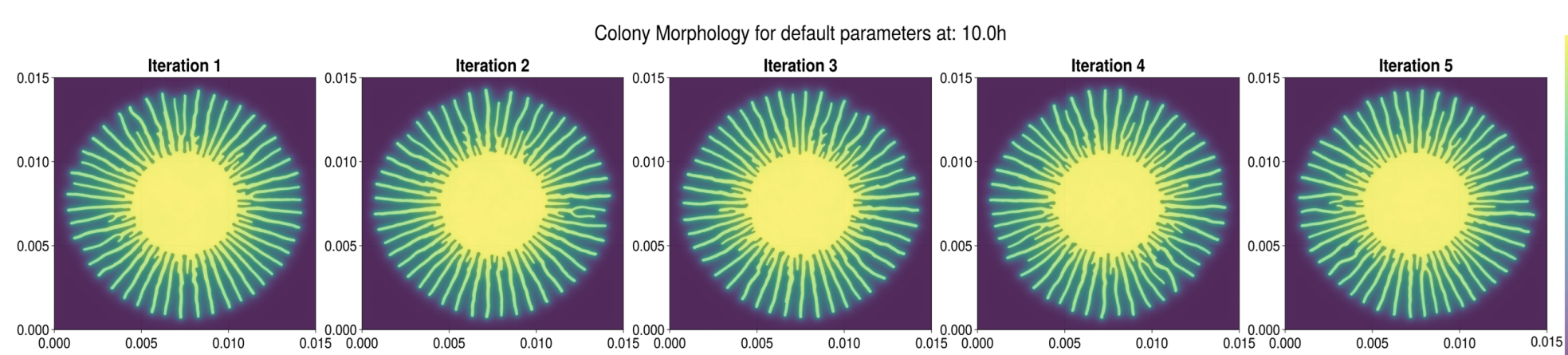

Fig A.2. Colony morphology for five iterations of the model after 10 h with default parameters (Table 1). For details of visualisation, see Materials and Methods.

One reason for this small variation is the large number of agents, which averages out the stochastic component (the noise term η_i in the direction update, equation 4).

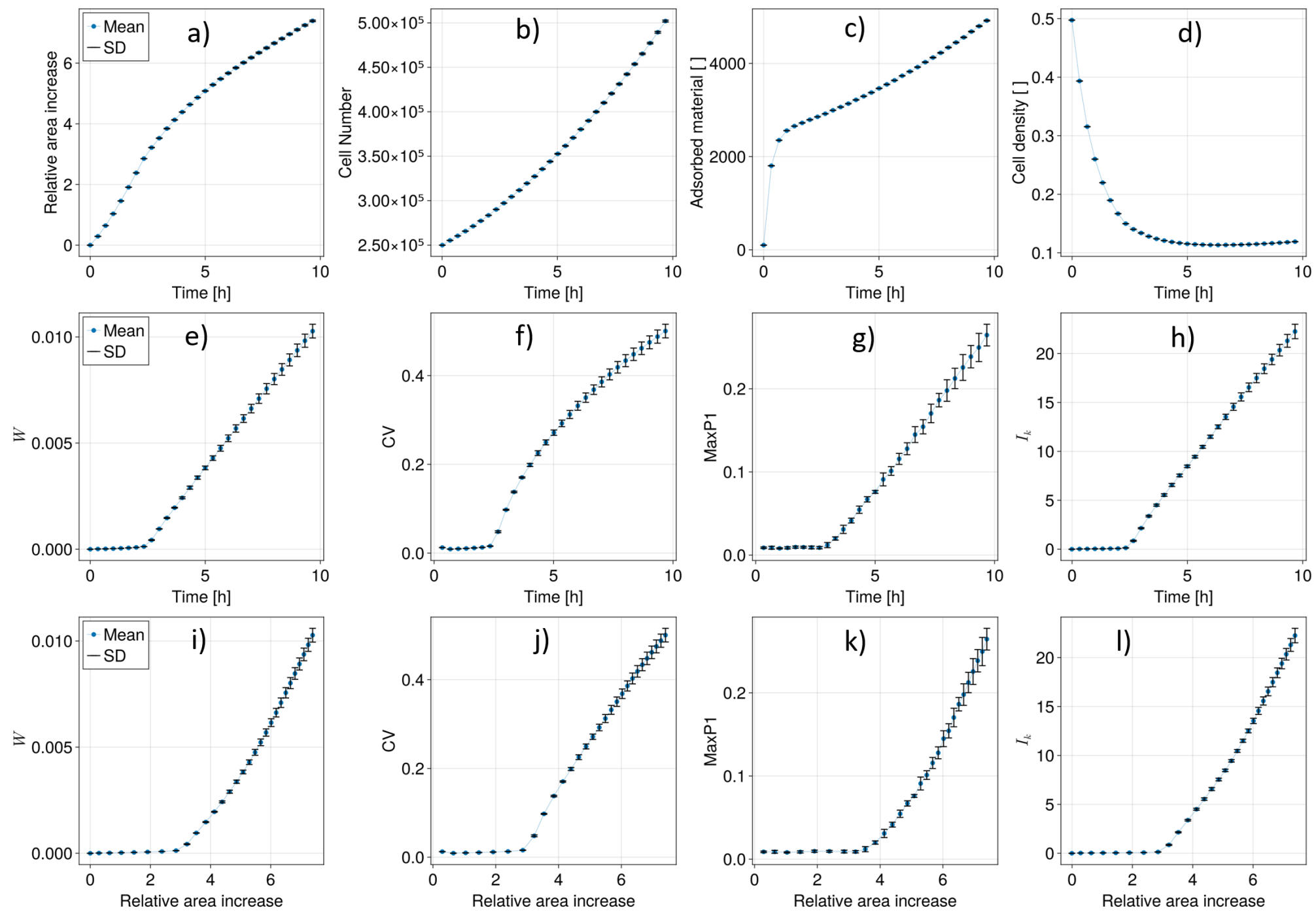

Fig A.3. Temporal evolution of mean morphological metrics with standard deviation over 10 h for default parameters (Table 1) for five iterations in 20-minutes intervals. (a) Relative area, (b) Cell number, (c) Adsorbed material, (d) Cell density within the colony, (e) Surface roughness W, (f) Coefficient of variation CV, (g) Relative maximum peak height $MaxP1$, and (h) Mean Fourier amplitude I_k. Evolution of mean morphological metrics with standard deviation relative to area increase: (i) Surface roughness W, (j) Coefficient of variation CV, (k) Relative maximum peak height $MaxP1$, and (l) Mean Fourier amplitude I_k.

A.5 Further metrics for grid and boundary properties

When varying the grid strength k_0 from -1000 to -25000 and the grid recovery rate g_{rc} from 0.0 to 25.0, cell number, adsorbed material and cell density showed behaviour similar to the default parameters. Therefore, they were not shown in the main manuscript in the subsection: **Grid and boundary properties affect expansion speed**, but can be seen here in Fig A.4.

A.6 Lattice Anisotropy

On-lattice cluster growth models can suffer from a phenomenon where the lattice symmetry becomes imprinted on the emerging structure, even with a completely random selection of growth sites [9–12]. In square lattices, this manifests as preferential growth along the major lattice axes (x and y) and their diagonals compared to other directions. This undesired anisotropy varies depending on the specific model and mitigation strategies employed. Most solutions require more complex surface site selection algorithms during the growth process to avoid anisotropic growth [10, 11, 13, 14].

Our on-lattice model of trypanosome colony geometry can be understood in a broader sense as a cluster growth model, although the colony does not ”grow” in the traditional sense. Instead, swimming trypanosomes push the colony boundary outward, with each agent influencing multiple grid cells inside the circular collision area when

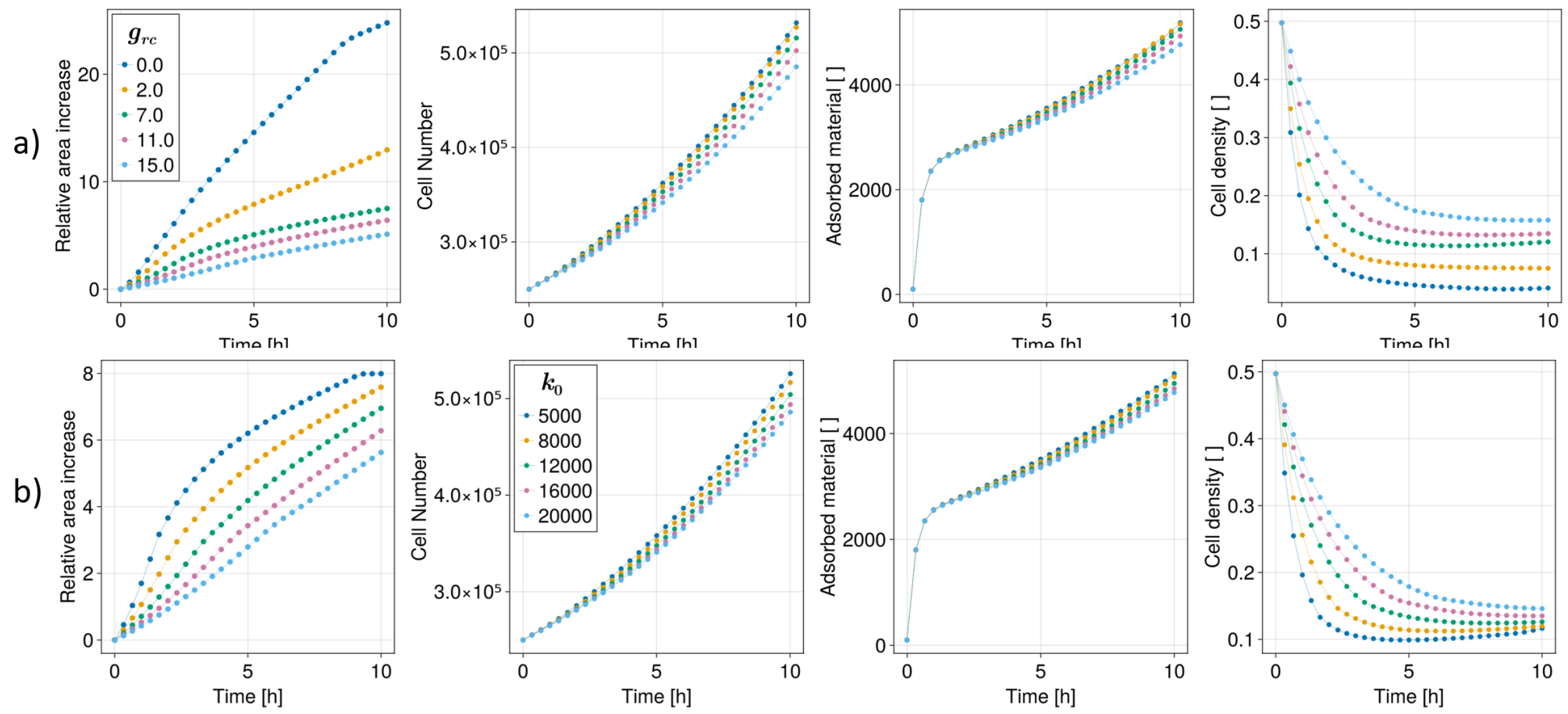

Fig A.4. Temporal evolution of morphological metrics (Relative area, Cell number, Adsorbed material and Cell density within the colony) over 10 h for default parameters with a) (a) five different grid recovery rate values g_{rc} and (b) five different absolute grid strength values $|k_0|$.

colliding with the boundary. Nevertheless, cell growth remains a significant factor enabling colony expansion.

To investigate whether colony anisotropies do emerge in our model and how they might be mitigated, we conducted a simulation with randomly moving agents and high boundary strength (similar to the noise reduction parameter m in Eden-like models, which enhance growth anisotropies [9]). The simulations were normalized to produce a similar relative area increase after 10 hours.

Anisotropy emerged when using a circular collision between agents and the boundary (Fig. A.5). The colony preferentially grows along the x and y lattice axes, visible as four distinct peaks in the angular metric.

Changing the collision geometry to a diamond shape reversed this trend completely, causing the colony to grow preferentially along the diagonals of the lattice. Conversely, a square-shaped collision area dramatically increased the initially observed preferential growth along the x and y axes.

Based on these findings, we implemented a hybrid collision area combining both circular and diamond geometries. This approach effectively balanced the competing anisotropies, resulting in more isotropic colony growth that better matches experimental observations.

References

1. Krüger T, Maus K, Kreß V, Meyer-Natus E, Engstler M. Single-cell motile behaviour of Trypanosoma brucei in thin-layered fluid collectives. The European Physical Journal E. 2021 Mar;44(3):37. doi:10.1140/epje/s10189-021-00052-7.

2. John D, Anderson J. Errors and an Analysis of Stability. In: Computational fluid dynamics: the basics with applications. McGraw Hill Education New York; 1995. p. 154-61.

3. Blazek J. Computational Fluid Dynamics: Principles and Applications. Butterworth-Heinemann; 2015.

4. Tu J, Yeoh GH, Liu C, Tao Y. Computational Fluid Dynamics: A Practical Approach. Elsevier; 2023.

5. Moin P. Fundamentals of Engineering Numerical Analysis. Cambridge University Press; 2010.

6. Kuhn A, Krüger T, Schüttler M, Engstler M, Fischer SC. Quantification of Trypanosoma brucei social motility indicates different colony growth phases. Journal of The Royal Society Interface. 2024 Dec;21(221):20240469. doi:10.1098/rsif.2024.0469.

7. Kay C, Peacock L, Gibson W. Trypanosoma congolense: In Vitro Culture and Transfection. Current Protocols in Microbiology. 2019;53(1):e77. _eprint: https://currentprotocols.onlinelibrary.wiley.com/doi/pdf/10.1002/cpmc.77. Available from: `https://onlinelibrary.wiley.com/doi/abs/10.1002/cpmc.77`. doi:10.1002/cpmc.77.

8. Binder BJ, Sundstrom JF, Gardner JM, Jiranek V, Oliver SG. Quantifying Two-Dimensional Filamentous and Invasive Growth Spatial Patterns in Yeast Colonies. PLOS Computational Biology. 2015 Feb;11(2):e1004070. doi:10.1371/journal.pcbi.1004070.

9. Batchelor MT, Henry BI. Limits to Eden growth in two and three dimensions. Physics Letters A. 1991 Jul;157(4):229-36. doi:10.1016/0375-9601(91)90057-F.

10. Bogoyavlenskiy VA. How to grow isotropic on-lattice diffusion-limited aggregates. Journal of Physics A: Mathematical and General. 2002 Mar;35(11):2533-8. doi:10.1088/0305-4470/35/11/301.

11. Alves SG, Jr SCF. Is it really possible to grow isotropic on-lattice diffusion-limited aggregates? Journal of Physics A: Mathematical and General. 2006 Mar;39(12):2843-52. doi:10.1088/0305-4470/39/12/001.

12. Paiva LR, Ferreira SC. Universality class of isotropic on-lattice Eden clusters. Journal of Physics A: Mathematical and Theoretical. 2006 Dec;40(1):F43. doi:10.1088/1751-8113/40/1/F05.

13. Yates CA, Baker RE. Isotropic model for cluster growth on a regular lattice. Physical Review E. 2013 Aug;88(2):023304. doi:10.1103/PhysRevE.88.023304.

14. Verhoef B, Hermsen R, De Graaf J. Fluid-derived lattices for unbiased modeling of bacterial colony growth; 2024. doi:10.1101/2024.12.23.630088.

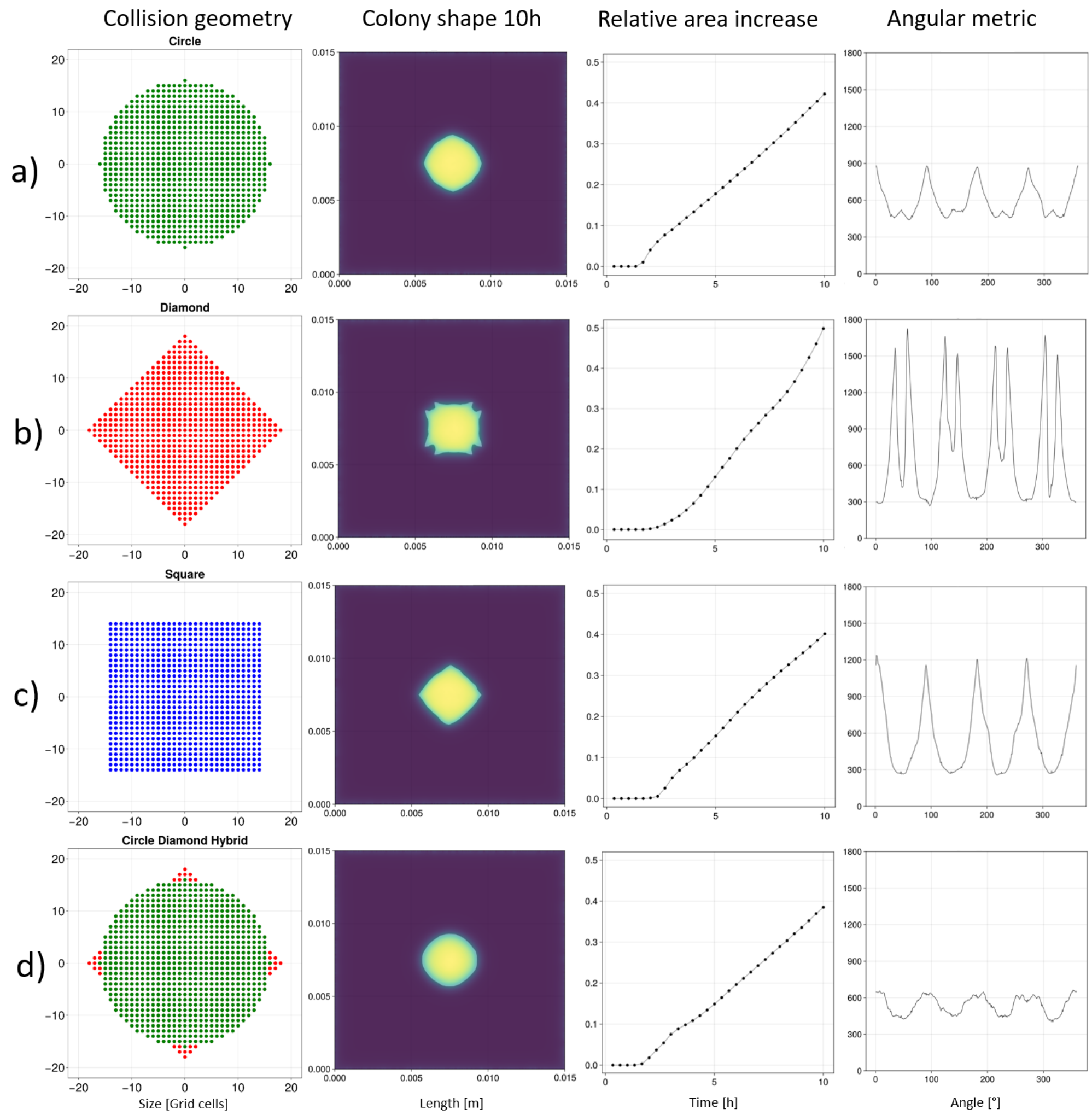

Fig A.5. Comparison of different collision area geometries and their effect on colony growth anisotropy, normalized for similar relative area gain over 10 hours. The angular metric shows the distinct periodic anisotropies in the surface morphology. (a) Using a circular collision area results in preferential growth along the x and y axes. (b) A diamond-shaped collision area reverses this trend, favouring diagonal growth. (c) A square-shaped collision area further enhances growth along the x and y axes. (d) Hybrid collision geometry combines circle and diamond shapes to produce the most isotropic growth.
