# Supplementary material for: An agent-based model of Trypanosoma brucei social motility to explore determinants of colony pattern formation
Source: PLoS Comput Biol. 2026 Aug 12;22(8):e1013698. doi: 10.1371/journal.pcbi.1013698 (PMC13489481; doi:10.1371/journal.pcbi.1013698)
Supplement: Supplementary file 2 [file pcbi.1013698.s002.pdf]

Review: An agent-based model of *Trypanosoma brucei* social motility to explore determinants of colony pattern formation

PLOS Computational Biology

Andreas Kuhn, Timothy Krüger, Markus Engstler, Sabine C. Fischer

Summary and Recommendation

This manuscript uses agent-based simulations implemented in `Julia` to model experimental colony patterns of the parasite *Trypanosoma brucei*. The mathematical model incorporates random motion and chemotaxis of *T. brucei* agents, a phenomenological treatment of boundary interactions, and explicitly models the concentration of a chemoattractant with a reaction–diffusion equation. Depending on model parameters, these features generate a wide variety of colony patterns, including the distinct thin fingering observed in experiments [1].

I enjoyed reading this paper. I think that the simulation results are interesting, and that the conclusions are mostly supported by the simulations shown. The `Julia` simulations will be useful to supplement future experiments as new candidate signalling molecules are identified. However, I have some questions that could be addressed in the text, and some aspects of the manuscript would benefit from further discussion and/or clarification. As such, I do not recommend publication as is, but instead recommend that a revision is carried out.

Major Comments

1. On p. 10, the manuscript includes a link to a GitHub repository containing the simulation code. However, the instructions provided under "How to Use" in this repository seem to be incorrect, and the documentation links to a 404 error. I did successfully run the file `run_im.jl` from the linked GitHub repository in `Julia 1.12.2`, but it did not generate the live animation. I think having a publicly-available code with documentation will be a strength of this work, but the authors should ensure that this potential is realised by checking and updating the instructions and documentation on GitHub before publication. They should also ensure that the provided code is sufficient to confirm that the results are reproducible, *e.g.* by including a version that can produce the visualisations.

2. In the model description and results sections, the manuscript would benefit from additional mechanistic explanations. These explanations would help to explain abstract features of the model, and to explain why the model yields certain trends in the spatial metrics in the results section. Specific examples of where this type of mechanistic explanation should be added include:
   - pp. 7–8: When describing the boundary model and k, what physical processes might the boundary strength and boundary weakening represent? As mentioned on pp. 11, 26–27 under (c), this might represent resistance to invasion through the agar medium. This concept should be discussed and expanded upon on p. 7–8 when the model is introduced.
   - pp. 13–15: With reference to features of the model, why does the initial circular growth phase cause rapid area increase but a reduction in cell density over the first ~ 4 hours? Is it related to the ease of weakening the boundary at early times, or the signalling gradient? Some discussion could be added to the text, for example surrounding Fig. 8 where this phenomenon is first seen.
   - p. 15–16: What might be causing the discrepancies between the simulation and experiment, especially for Experiment 1?
   - p. 17: Why does the orientation noise have these effects on morphology, cell density, and colony area? Perhaps the extra noise favours random motion rather than movement aligned with the chemical gradient, which reduces the collisions with the boundary and hence fingering. Additional discussion should be added surrounding Fig. 12.
   - p. 18: Similarly, a mechanistic explanation for how the turning rate influences morphology, cell density, and colony area should be added after Fig. 14.

- p. 22: Why do to k and g_{rc} parameters have these effects on morphology?

3. *T. brucei* are modelled as point agents, but as Figure S1 shows the individuals actually have an elongated rod-like shape with a fairly large aspect ratio. Although the agent's shape must presumably relate to its orientation (which should be emphasised in-text, if so), in on-lattice agent-based models the agent shape itself can impact how the population spreads (see [2], for example), as well as the volume exclusion rule. At present, this limitation is not addressed in the manuscript. The authors should add significant discussion about the limitation of neglecting agent shape, and comment on how this might affect our interpretation of the results, and/or possible discrepancies between the simulations and experiments. In practice, would the agent shape be related to its orientation?

4. The model does not consider nutrients explicitly, and instead effectively considers nutrients to be abundant and non-depleting by allowing each agent to proliferate with a constant rate r. The results in [1] show exponential area increase, but not necessarily an exponential increase in cell number. Based on results for other microbes, the rates of area increase in these colonies might be sufficiently large for nutrient depletion or gradients to become relevant [3]. Nutrient depletion would reduce the effective proliferation rate, and nutrient gradients can impact finger-like morphologies in bacteria [4]. Is it possible for nutrient depletion to impact the experimental results in a way that the model does not capture? If so, it could explain the faster growth observed in simulations compared to experiments. I think some discussion of this potential issue is needed in the manuscript.

Minor Comments

1. p. 3–8: To reinforce that the results arise from the model mechanisms rather than artefacts in the numerics, it might be useful to provide more details of how numerical parameters, *e.g.* Δx and s_f, affect the results (*e.g.* in the Appendix). Was there any significant reason for choosing to allow 4 boundary interactions per time step, or the particular choice of R_t? Does varying these parameters impact the results?

2. pp. 5–6: What were the boundary conditions associated with the reaction–diffusion equation for the chemoattractant, s (Eqn. 1)? How were they implemented numerically? The initial condition for s is said to be a 'small, randomly distributed chemical concentration'. In precise terms, what was the initial distribution for s?

3. p. 8: How likely is the fallback mechanism of reversal at the boundary to occur in the simulations?

4. p. 12: Although the spatial statistics were published elsewhere, it would be helpful to add one sentence each to this paper on what W, CV, $MaxPI$, and I_k specifically tell us about the morphology, so that the reader can follow the graphs without having to refer back to [1].

5. p. 16: For the ease of comparison, it would be useful to plot Figures 10 and 11 with the same vertical scales (or on the same axes), and to have the experimental doubling time included in the legend.

6. p. 17: It might be possible to draw an in-passing comparison between the non-*T. brucei* morphologies, which are themselves interesting simulations, and patterns seen in other microbes. For example, in Fig. 12 the leftmost pattern is similar to pseudohyphal growth of yeast colonies [5], [6], and the fourth panel resembles a 'dense branching morphology' with tip-splitting, *e.g.* [7].

7. p. 19: The information under the heading 'Small range of suitable parameter values for turning rate and orientation noise' probably does not warrant its own section, given that many of the points were already made in the preceding two sections and Fig. 16 could be appended to the second of these.

8. p. 22: I don't quite agree with the interpretation of Figure 17 that 'whilst a lower g_{rc} also causes the expansion behaviour to change to circular growth, a lower $|k_0|$ does not appear to change the expansion pattern'. It looks like increasing $|k_0|$ strongly suppresses the fingering pattern, so the intended meaning of this statement should be made clearer. My comment also impacts the discussion at the bottom of p. 26, where a similar sentence appears.

9. p. 22: In some of the simulations (*e.g.* the bottom left panel in Fig. 17, but also many other results), the colonies grow close enough to the boundaries that boundary effects will impact the spatial statistics at later times. Although I don't believe that boundary effects would impact the qualitative morphologies, they may still impact the simulations (*e.g.* the big fingers near the cardinal directions in the bottom right colonies of Fig. 20), so they should be acknowledged when discussing both the simulated colonies and the spatial statistics.

10. p. 27: When mentioning the quantitative predictions of D_s and the comparison to hypothesised signals, it might be worth mentioning that the model's assumptions could impact the accuracy of the predictions. For example, if the expansion speed in experiments is slower than in simulations, perhaps in practice slower diffusion than the model's prediction might still allow a colony-wide gradient in the signalling chemical to occur.

11. Some typographical comments that I came across:

- p. 3: The 2D position $\boldsymbol{x}(t)$ is bolded, and this convention of bolding the vector $\boldsymbol{x}$ should continue throughout the rest of the paper.
- p. 16: Letter labels are missing from Fig. 11.
- p. 17: There is a grammatical error 'Noise's' in the figure title.
- p. 25: Section number missing from Fig 21 and the caption should read '20-minute intervals'.
- There are a few instances of very short paragraphs, where the paragraph breaks do not introduce a new topic. These should be revised.
- The names of all microbial species should be italicised.
- Consistent terminology for the spatial statistics should be used. In different figures and text, these are referred to as 'colony metrics', 'morphological metrics', or 'colony properties'.

Bibliography

[1] A. Kuhn, T. Krüger, M. Schüttler, M. Engstler, and S. C. Fischer, "Quantification of Trypanosoma Brucei Social Motility Indicates Different Colony Growth Phases," *J. R. Soc. Interface*, vol. 21, no. 221, p. 20240469, 2024, doi: 10.1098/rsif.2024.0469.

[2] M. J. Simpson, R. E. Baker, and S. W. McCue, "Models of Collective Cell Spreading with Variable Cell Aspect Ratio: A Motivation for Degenerate Diffusion Models," *Phys. Rev. E*, vol. 83, no. 2, p. 21901, 2011, doi: 10.1103/PhysRevE.83.021901.

[3] H. Tronnolone *et al.*, "Diffusion-Limited Growth of Microbial Colonies," *Sci. Rep.*, vol. 8, p. 5992, 2018, doi: 10.1038/s41598-018-23649-z.

[4] H. Fujikawa and M. Matsushita, "Bacterial Fractal Growth in the Concentration Field of Nutrient," *J. Phys. Soc. Jpn.*, vol. 60, no. 1, pp. 88–94, 1991, doi: 10.1143/JPSJ.60.88.

[5] B. J. Binder, J. F. Sundstrom, J. M. Gardner, V. Jiranek, and S. G. Oliver, "Quantifying Two-Dimensional Filamentous and Invasive Growth Spatial Patterns in Yeast Colonies," *PLOS Comput. Biol.*, vol. 11, no. 2, p. e1004070, 2015, doi: 10.1371/journal.pcbi.1004070.

[6] K. Li *et al.*, "An Off-Lattice Discrete Model to Characterise Filamentous Yeast Colony Morphology," *PLOS Comput. Biol.*, vol. 20, no. 11, p. e1012605, 2024, doi: 10.1371/journal.pcbi.1012605.

[7] E. Ben-Jacob, I. Cohen, and H. Levine, "Cooperative Self-Organization of Microorganisms," *Adv. Phys.*, vol. 49, no. 4, pp. 395–554, 2000, doi: 10.1080/000187300405228.
