# Supplementary material for: An agent-based model of Trypanosoma brucei social motility to explore determinants of colony pattern formation
Source: PLoS Comput Biol. 2026 Aug 12;22(8):e1013698. doi: 10.1371/journal.pcbi.1013698 (PMC13489481; doi:10.1371/journal.pcbi.1013698)
Supplement: Supplementary file 3 [file pcbi.1013698.s003.pdf]

Manuscript Title: An agent-based model of *Trypanosoma brucei* social motility to explore determinants of colony pattern formation **Manuscript ID:** PCOMPBIOL-D-25-02262 **Journal:** *PLOS Computational Biology*

Dear Dr. Banga and the Editorial Team,

We would like to thank the editors and the reviewers for their constructive feedback and the opportunity to revise our manuscript. We have addressed all the editorial requirements and responded to the comments provided by each of the four reviewers below.

We believe these revisions address all concerns raised by the reviewers and hope the manuscript is now suitable for publication in *PLOS Computational Biology*.

Sincerely,

Sabine C. Fischer (on behalf of the authors)

Part 1: Responses to Editorial Requirements

[Editor Comment 1]: Complete CRediT author contributions for all authors.

[Author Response]: We have updated the submission form to ensure that the contributions for Andreas Kuhn, Timothy Krüger, Markus Engstler, and Sabine C. Fischer are fully acknowledged.

[Editor Comment 2]: Provide necessary files.

[Author Response]: We provide:

- the rebuttal letter that responds to each point raised by the editor and reviewers
- A marked-up copy of our manuscript that highlights changes made to the original version. In .pdf and .tex format.
- An unmarked version of your revised paper without tracked changes.

[Author Response]: All figures have been uploaded as separate files as .tif files.

[Editor Comment 4]: Remove supplementary Figures from the manuscript file and upload as 'Supporting Information'.

[Author Response]: Supplementary figures have been moved to separate files.

[Editor Comment 5]: Amend Financial Disclosure statement to include initials and full sentences.

[Author Response]: The statement has been revised to: "This project was funded by Project 492009575 of the Priority programme 2332 'Physics of Parasitism' of the German Research Foundation (DFG) to S.C.F.. The funders had no role in study design, data collection and analysis, decision to publish, or preparation of the manuscript."

[Editor Comment 6]: Update Data Availability Statement.

[Author Response]: The code is available at https://github.com/AndreasKuhn-ak/2026-Kuhn-et-al and will be archived at Zenodo upon publication.

Part 2: Responses to Reviewer #1

[Point 1]: While reading this paper I thought if a recent work I read Xue, Budrene, and Othmer that looks at radial fingering patterns in Proteus mirabilis. It may be worth citing or looking at for comparative purposes.

[Author Response]: Thanks for this suggestion. We were not aware of this system. We have added a paragraph in the introduction highlighting the similarities and differences.

[Point 2]: In all the images, it seems the boundary fingers were more or less the same length throughout time. Is there any scenario where this wouldn't happen, such as inhomogeneous colony boundary expansion conditions? That might be an interesting simulation to look at with the model in the future.

[Author Response]: While the current simulations show relatively uniform finger lengths, our model is capable of exploring inhomogeneous conditions, as the value of k that defines the boundary strength is currently uniform over the simulation space, but could be locally adjusted to account for inhomogeneities in the environment. We discussed this in the new Model Design Choices section in the discussion.

[Point 3a]: You mention that the model simulations grow faster outward than experimental data. Could this be fixed by adjusting the grid strength on the boundary interactions? I assume it is more complicated than that given that you probably tried that.

[Author Response]: Yes and no. Our parameter sensitivity analysis demonstrates that increasing the absolute grid strength $|k_0|$ reduces expansion speed without substantially altering the overall behaviour. The same holds for g_{rc}, albeit to a lesser extent. Consequently, there should exist parameter values for $|k_0|$ and g_{rc} that fit the experimental data better. The challenge lies in identifying these values, as we have already invested considerable time in determining the default parameters. Computational cost quickly becomes the limiting factor. We were therefore initially satisfied with establishing the default parameters and characterising boundary behaviour. However, our planned model extension prioritises rewriting portions of the code for GPU implementation to drastically reduce computation time, followed by simulation-based inference methods to improve data fitting. We discussed this in the new future direction part in the discussion.

[Point 3b]: Would incorporating some kind of excluded-volume constraint on the individual bacteria affect the expansion rate or patterning? It would make it computationally more difficult, but collisions or jamming could change the patterns observed.

[Author Response]: Currently, the model permits only one agent per ABM grid cell, providing a simple volume-exclusion mechanism. A full excluded-volume constraint accounting for rod-shaped agents would likely alter both expansion rate and patterning, with effects potentially tunable depending on the specific parameterisation. However, this was not implemented due to the computational efficiency requirements for an agent-based model of this scale. We discussed this in the new Model Design Choices section in the discussion.

[Point 4]: Do you believe this is generalizable to other microorganisms or possibly it depends on their movement mechanism, which may be unique to the strain studied?

[Author Response]: We believe our approach is generalisable. Specifically, we note that *Pseudomonas aeruginosa* exhibits colony morphology and microswimmer dynamics similar to those of *T. brucei*, suggesting that similarities at the microscale can produce similarities at the macroscale. However, many model parameters would require adjustment—for example, the lattice spacing (corresponding to agent size), as *Pseudomonas* cells are approximately 10× smaller than *T. brucei*. Additionally, the exact movement characteristics of *Pseudomonas* within colonies would need to be determined experimentally before quantitative predictions could be made. We discussed this in the new future direction part of the discussion.

[Minor Points]: 1. Mathematical equations should be treated as part of a sentence. So, if a sentence ends with an equation, that equation should end with a period. If it is in the middle of a sentence often a comma can be found at the end of the equation.

2. In Line 132, I would suggest a central dot. In equation editor you can use \cdot, so in equation editor it could be \delta(\cdot).

3. Line 605, fix the first quotation mark.

[Author Response]: We have corrected the punctuation of equations, updated the Dirac delta notation to $\delta(\cdot)$, and fixed the formatting of the quotation marks.

Part 3: Responses to Reviewer #2

[Point 1]: The main question I have is around the importance of features in the model. By varying parameters, the authors find that the chemical diffusion has a crucial role in the colony shape. Other models have used a similar approach but without the complex boundary mechanism introduced here and have produced similar fingering patterns. Could the authors comment on whether the patterns produced are due to the boundary mechanism or could be the result of diffusion limited growth. I believe this must be addressed satisfactorily for the paper to be published.

[Author Response]: Although bacterial colony patterns caused by diffusion-limited growth can appear morphologically similar to expanding trypanosome colonies, the underlying mechanisms are fundamentally different. In diffusion-limited growth, bacteria are largely sessile and proliferate preferentially at colony boundaries where nutrient availability exceeds that in the colony interior. In contrast, trypanosome colonies are dynamic entities in which cells move continuously, pausing only briefly at colony boundaries. Crucially, nutrients are not a limiting factor in these systems, and cells divide at a rate independent of their position within the colony. Colony cohesion is maintained primarily by hydrodynamic and mechanical forces—simulated through our boundary implementation—whilst outward expansion is driven by cellular motility. Thus, the observed pattern formation results from the interplay between boundary mechanics and autochemotactic alignment, which creates a preferred outward flux from the colony centre. We discussed this in the introduction.

[Point 2]: I would also like the authors to comment on the approach for parameter exploration. Was consideration given to a more formal approach, such as approximate Bayesian computation? This would give a more systematic comparison.

[Author Response]: This is something we have considered extensively and represents one of the most important future goals for this project. More formal approaches such as simulation-based inference (of which approximate Bayesian computation is a subset) typically require many iterative simulation runs to refine parameter values and improve agreement with experimental data. At present, the computational performance of the model is insufficient to complete this within a reasonable timeframe. Therefore, our immediate priority is to improve model performance by rewriting portions of the code for GPU implementation, after which we intend to employ such formal methods. We discussed this in the new future direction part of the discussion.

[Point 3a]: The variable t very likely represents time but should be clearly defined.

[Author Response]: We have explicitly defined t as time in the “Calculate diffusion” section of the Methods.

[Point 3b]: Does the gradient grid within one ABM cell have the same concentration? This was not clear and Figure 1 appears to suggest this is the case, which would seemingly remove the purpose of the finer grid.

[Author [Response]: It is the other way around. One gradient grid cell can map to 1,4,9,16,… finer ABM grid cells, depending on the scaling factor. We revise the part of the text in the “Model Architecture “ section that explains this relationship to make it clearer.

[Point 4]: Are the cell angles restricted in any way? How is this converted to a direction on the lattice?

[Author Response]: The agents are point-like particles characterized solely by their position and movement direction, without explicit geometric angles. At each time step Δt_a, both position and direction are updated: an agent moves from its current position $\mathbf{x}_i(t)$in the direction $\theta_i(t+\Delta t_a)$with velocity v_i, which is sampled from a normal distribution with mean $v_0$and standard deviation v_σ. The displacement

is calculated in continuous space as $\mathbf{x}_i(t + \Delta t_a) = \mathbf{x}_i(t) + v_i \Delta t_a [\cos\ \theta_i(t + \Delta t_a), \sin\ \theta_i(t + \Delta t_a)]^T$. Since the resulting position $\mathbf{x}(t + \Delta t_a)$is a continuous two-dimensional vector, it is mapped to the discrete ABM grid by rounding each component to the nearest grid cell. The direction θ_i itself is unconstrained, is never rounded, and can take any value in the $[0,2\pi)$, allowing free rotation in continuous angular space before discretisation onto the lattice occurs through the position update. We added a sentence in the Agent update section to clarify that.

[Point 5]: Is the abbreviation “SoMo” used later? Perhaps this does not need to be introduced. I would recommend being consistent with the capitalisation of “Social Motility”.

[Author Response]: We removed the abbreviation SoMo and changed it to the capitalization of social mobility to be consistent.

[Point 6]: Figure 3, It would be useful to include a statement in the caption to explicitly state what the green cells represent.

[Author Response]: We changed the figure caption in Figure 3.

[Point 7]: How are Rt and Rc chosen? While these are in category c, was there any motivation or are these just selected to make the model work?

[Author Response]:
We added a paragraph at the end of the boundary interaction section to further describe their purpose and a section in the appendix discussed these chosen values.

[Point 8]: Figure 4: If the shape used in the model is not a circle, I would update the figure to illustrate this.

[Author Response]: We changed Figure 4 and adapted the figure description.

[Point 8]: When specifying CPU performance, please add what a 2025 CPU represents in terms of computing power.

[Author Response]: We have added technical specifications for the "2025 CPU".

[Point 9]: Table 1: Stray *, which I believe is standing in for a multiplication sign. Consider expressing the absorption rate and decay rate in scientific notation too.

[Author Response]: We changed that.

[Point 10]: It’s noted that the variation between simulations was “remarkably small”. Can you quantify this? It would help the reader judge the impact of the choice not to repeat simulations. Could standard deviations be reported for one case?

[Author Response]: We added a new section to the appendix where we simulated the model 5 times with default parameter values and calculated the mean and standard deviation of all analyzed quantities. It can be seen that the variation in all these quantities, as well as in the emerging colony morphology, is very small across model runs with identical parameters.

[Point 11]: Can you explain why the faster-growing colony produces more fingers and more uniform fingers?

[Author Response]: We can only speculate at present. A faster-growing colony adsorbs more chemotactic material, creating a stronger outward-pointing gradient whilst the colony is initially confined to approximately the same area. This stronger gradient enhances the tendency of cells to move towards boundaries and drive outward expansion, resulting in more fingers. Additionally, higher cell density could make this process more spatially homogeneous throughout the colony, producing more regular finger spacing.
To confirm this hypothesis, we would need to analyse individual agent trajectories to verify whether faster-growing colonies indeed exhibit stronger boundary-directed motion. The model is capable of providing such trajectory data, but extracting and analysing it will require substantial modifications to our analysis pipeline—work we plan to undertake in future studies. We discussed this in the new **Model Design Choices: Heterogeneous conditions** and **Future directions: Trajectory analysis** part of the discussion.

[Point 12]: The formulation seems to switch between measuring angles in degrees and radians. One of these should be selected and used consistently.

[Author Response]: We switched to radians everywhere.

[Point 13]: Figure 12: Would the colony with noise 0.75 later form fingers if simulated for longer?

[Author Response]: No. When the noise level is too high, the agents effectively move in a total random manner, and therefore, finger formation is completely suppressed, and the colony expands slowly in a circular fashion indefinitely We clarified this in the "Orientation noise affects colony morphology" part in the results section .

[Point 14]: It's claimed that a visual analysis indicates the best experimental match when eta is 0.2 and alpha is 0.02-0.1. Do the metrics computed also support this claim?

[Author Response]: Yes, and we have now mentioned this in the "Turning rate affects expansion speed" section. We opted not to show the metrics themselves, as displaying all four metrics for the 16 tested parameter value pairs would create an enormous figure that is very hard to grasp and interpret. Since the metrics essentially only quantify the fingering pattern behaviour, it is not surprising that the colonies that look most similar to experiments are also the most similar in the metrics. Therefore, we believe this simplification is justified.

Part 4: Responses to Reviewer #3 (Review4.pdf)

[Major Point 1]: On p. 10, the manuscript includes a link to a GitHub repository containing the simulation code. However, the instructions provided under "How to Use" in this repository seem to be incorrect, and the documentation links to a 404 error. I did successfully run the file run_im.jl from the linked GitHub repository in Julia 1.12.2, but it did not generate the live animation. I think having a publicly-available code with documentation will be a strength of this work, but the authors should ensure that this potential is realized by checking and updating the instructions and documentation on GitHub before publication. They should also ensure that the provided code is sufficient to confirm that the results are reproducible, e.g. by including a version that can produce the visualisations.

[Author Response]: We have expanded and updated the repository documentation, providing step-by-step instructions for both interactive model exploration and automated parameter sweeps. The reworked codebase now supports two usage modes: an interactive interface for single-run experimentation and testing, and a parameter sweep framework for systematic exploration. Both modes are fully documented, and the associated analysis notebooks are usable for any researcher wishing to reproduce or extend our results.

[Major Point 2]: In the model description and results sections, the manuscript would benefit from additional mechanistic explanations. These explanations would help to explain abstract features of the

model, and to explain why the model yields certain trends in the spatial metrics in the results section. Specific examples of where this type of mechanistic explanation should be added include:

1. pp. 7–8: When describing the boundary model and k, what physical processes might the boundary strength and boundary weakening represent? As mentioned on pp. 11, 26–27 under (c), this might represent resistance to invasion through the agar medium. This concept should be discussed and expanded upon on p. 7–8 when the model is introduced.

[Author Response]: We expanded pages 7–8 to clarify that the grid strength k, the grid recovery rate grc, and the agent collision mechanism constitute a phenomenological model of the complex hydrodynamic interface between the semi-fluid medium (agarose) and the fluid medium (nutrient solution containing trypanosomes). This interface can exchange liquid vertically to increase the volume of the fluid medium whilst remaining laterally separated and maintaining cohesion through surface tension. Meanwhile, the trypanosomes actively drive outward expansion of the fluid medium through organised movement.

2. pp. 13–15: With reference to features of the model, why does the initial circular growth phase cause rapid area increase but a reduction in cell density over the first ∼4 hours? Is it related to the ease of weakening the boundary at early times, or the signalling gradient? Some discussion could be added to the text, for example surrounding Fig. 8 where this phenomenon is first seen.

[Author Response]: We expanded the section at the beginning of the results part, explaining this behavior.

3. p. 15–16: What might be causing the discrepancies between the simulation and experiment, especially for Experiment 1?

[Author Response]: The discrepancies are quantitative rather than qualitative: simulated colonies grow too rapidly and produce more fingers than observed experimentally, but they correctly exhibit finger formation and two-phase expansion. We believe that more thorough exploration of the parameter space would yield parameter values that better match the experimental data. However, as discussed in previous responses, computational performance currently limits such extensive parameter sweeps. We discussed this in the new sections of the discussion (Parameter Hierarchy and Pattern Formation, Simulation-based inference).

4. p. 17: Why does the orientation noise have these effects on morphology, cell density, and colony area? Perhaps the extra noise favours random motion rather than movement aligned with the chemical gradient, which reduces the collisions with the boundary and hence fingering. Additional discussion should be added surrounding Fig. 12.

[Author Response]: We expanded the section surrounding Fig. 12.

5. p. 18: Similarly, a mechanistic explanation for how the turning rate influences morphology, cell density, and colony area should be added after Fig. 14.

[Author Response]: We expanded the section surrounding Fig. 14.

6. p. 22: Why do to k and grc parameters have these effects on morphology?

[Author Response]: We expanded the section surrounding Fig. 20.

[Major Point 3]: T. brucei are modelled as point agents, but as Figure S1 shows the individuals actually have an elongated rod-like shape with a fairly large aspect ratio. Although the agent's shape must presumably relate to its orientation (which should be emphasised in-text, if so), in on-lattice agent-based models the agent shape itself can impact how the population spreads (see [2], for example), as well as the volume exclusion rule. At present, this limitation is not addressed in the manuscript. The authors should add significant discussion about the limitation of neglecting agent shape, and comment on how this might affect our interpretation of the results, and/or possible discrepancies between the simulations and experiments. In practice, would the agent shape be related to its orientation?

[Author Response]: We have added a significant Model Design Choices section in the discussion where we discussed this limitation, amongst others. To answer your last question, yes, the agent shape would be related to its orientation, as trypanosomes swim by pulling themselves forwards with their flagellum at the front part of the cell towards the direction where their rod-like shape is pointed.

[Major Point 4]:
The model does not consider nutrients explicitly, and instead effectively considers nutrients to be abundant and non-depleting by allowing each agent to proliferate with a constant rate r. The results in [1] show exponential area increase, but not necessarily an exponential increase in cell number. Based on results for other microbes, the rates of area increase in these colonies might be sufficiently large for nutrient depletion or gradients to become relevant [3]. Nutrient depletion would reduce the effective proliferation rate, and nutrient gradients can impact finger-like morphologies in bacteria [4]. Is it possible for nutrient depletion to impact the experimental results in a way that the model does not capture? If so, it could explain the faster growth observed in simulations compared to experiments. I think some discussion of this potential issue is needed in the manuscript.

[Author Response]: We have added a significant Model Design Choices section where we discussed this limitation, amongst others.

[Minor Points]:

1. p. 3–8: To reinforce that the results arise from the model mechanisms rather than artefacts in the numerics, it might be useful to provide more details of how numerical parameters, e.g. Δx and sf, affect the results (e.g. in the Appendix). Was there any significant reason for choosing to allow 4 boundary interactions per time step, or the particular choice of Rt? Does varying these parameters impact the results?

[Author Response]: As Δx^2 also represents the projected size of an agent, we did not vary this parameter; doing so would necessitate changes to the volume exclusion mechanism and other coupled parameters. This was therefore treated as a fixed parameter of the model (see section "Area of Cells" in the appendix). For the other mentioned parameters and mechanisms, we have added a section in the appendix discussing their selection.

2. pp. 5–6: What were the boundary conditions associated with the reaction–diffusion equation for the chemoattractant, s (Eqn. 1)? How were they implemented numerically? The initial condition for s is said to be a 'small, randomly distributed chemical concentration'. In precise terms, what was the initial distribution for s?

[Author Response]: We used Dirichlet boundary conditions with a fixed value of s on the boundary. The initial condition for s is drawn from a uniform distribution between 10^{-6} and 2 * 10^{-6}. These values are much smaller than the amount of s secreted by the agents; therefore, they do not change

the system behaviour, but they avoid numerical artefacts that can occur when initialised with zero due to limited floating-point precision. We clarify that in the "Initialisation" section and the "Calculate diffusion"section.

3. p. 8: How likely is the fallback mechanism of reversal at the boundary to occur in the simulations?

[Author Response]: We have clarified the section regarding the reflection mechanism and the fallback mechanism, including quantitative information on the frequency of fallback invocation.

4. p. 12: Although the spatial statistics were published elsewhere, it would be helpful to add one sentence each to this paper on what W, CV, $MaxPI$, and Ik specifically tell us about the morphology, so that the reader can follow the graphs without having to refer back to [1].

[Author Response]: We added one sentence to each at the end of the methods section.

5. p. 16: For the ease of comparison, it would be useful to plot Figures 10 and 11 with the same vertical scales (or on the same axes), and to have the experimental doubling time included in the legend.

[Author Response]: We merged Figure 10 and Figure 11 into one Figure (now Figure 11) where the experimental data is shown along the simulated colonies. We also included the experimental doubling time in the figure legend.

6. p. 17: It might be possible to draw an in-passing comparison between the non-T. brucei morphologies, which are themselves interesting simulations, and patterns seen in other microbes. For example, in Fig. 12 the leftmost pattern is similar to pseudohyphal growth of yeast colonies [5], [6], and the fourth panel resembles a 'dense branching morphology' with tip-splitting, e.g. [7].

[Author Response]: We added a new extended section in the discussion regarding the application to other systems.

7. p. 19: The information under the heading 'Small range of suitable parameter values for turning rate and orientation noise' probably does not warrant its own section, given that many of the points were already made in the preceding two sections and Fig. 16 could be appended to the second of these.

[Author Response]: We removed this section and added the content to the previous section.

8. p. 22: I don't quite agree with the interpretation of Figure 17 that 'whilst a lower grc also causes the expansion behaviour to change to circular growth, a lower $|k0|$ does not appear to change the expansion pattern'. It looks like increasing $|k0|$ strongly suppresses the fingering pattern, so the intended meaning of this statement should be made clearer. My comment also impacts the discussion at the bottom of p. 26, where a similar sentence appears.

[Author Response]: This requires clarification. In Fig. 17, the suppression of fingering patterns at higher | k_0 |values primarily reflect a temporal delay rather than a fundamental change in morphology. This distinction becomes clear in Fig. 19, where metrics are plotted against both time and relative area increase. When plotted against time, increasing | k_0 |appears to suppress fingering because expansion occurs more slowly. However, when the same metrics are plotted against relative area increase, all curves for different | k_0 |values align closely, demonstrating that colonies eventually develop similar morphologies—they simply reach these morphologies at different times. In contrast, low values of g_{rc}(particularly $g_{rc} = 0.0$ and $g_{rc} = 2.0$) produce fundamentally different morphologies even when

normalised by area increase, indicating a qualitative rather than merely temporal change in expansion behaviour. Thus, | k_0 |primarily affects the expansion timescale, whilst only very low g_{rc}values alter the fundamental nature of the expansion pattern from finger-like to circular growth. We discussed this further in the new Parameter Hierarchy and Pattern Formation section in the discussion.

9. p. 22: In some of the simulations (e.g. the bottom left panel in Fig. 17, but also many other results), the colonies grow close enough to the boundaries that boundary effects will impact the spatial statistics at later times. Although I don't believe that boundary effects would impact the qualitative morphologies, they may still impact the simulations (e.g. the big fingers near the cardinal directions in the bottom right colonies of Fig. 20), so they should be acknowledged when discussing both the simulated colonies and the spatial statistics.

[Author Response]: Although some simulated colonies shown in the paper grow close to the edge of the simulation space, none actually reached it. We systematically excluded all simulations where colonies reached the boundary (e.g., for very small values of | k_0 |or g_{rc}), as we believe the parameter ranges shown already capture the relevant system behaviour. Regarding the reviewer's observation about preferential finger growth near cardinal directions in some colonies (e.g., bottom right of Fig. 20): this pattern arises from the discrete lattice structure of the ABM grid rather than boundary proximity effects. The square lattice geometry introduces slight anisotropies that can favour growth along the cardinal and diagonal directions, particularly when combined with the collision radius $R_c$and the discrete nature of agent movement. As shown in Fig. S2 in the appendix, even with the implemented correction, there is still a slight preference for growth along the carnial directions. We have added a sentence at the beginning of the Methods section clarifying that all simulations shown in this work did not reach the boundary, and we now also acknowledge this lattice anisotropy effect in the description of Figure 20.

10. p. 27: When mentioning the quantitative predictions of Ds and the comparison to hypothesised signals, it might be worth mentioning that the model's assumptions could impact the accuracy of the predictions. For example, if the expansion speed in experiments is slower than in simulations, perhaps in practice, slower diffusion than the model's prediction might still allow a colony-wide gradient in the signalling chemical to occur.

[Author Response]: We added a sentence clarifying that in the discussion section.

Some typographical comments that I came across: ….

[Author Response]: We fixed all of those.

Part 5: Responses to Reviewer #4

[Major Point 1]: 1. Show experimental data. The key element of simulations is to reproduce experimental patterns. However, for me to know what the pattern looks like I had to go to the Kuhn et al. 2024 J Roy Soc Int to look it up. I think showing examples of the patterns formed, e.g., next to photo of the parasite (Fig S1), would be very useful. Fig 11 does show experimental data (well, some metrics extracted from the images), but they are hard to see or compare to simulations. Connecting points with lines and making sure y/x axes scales are the same between experiment and simulations could be useful. Also, for experiments, you need to provide much more information on how experiments/measurements were done than Fig 11 caption suggests. Also include these descriptions in Materials and methods.

[Author Response]: We have added a time series fluorescence image showing a Trypanosoma colony exhibiting social motility to the Introduction (Fig. 1), which demonstrates the characteristic transition from circular to finger-like growth. Additionally, we have included a detailed summary of the experimental methods from our published work in the appendix (S3), describing cell culture conditions, motility assay preparation, and image acquisition protocols. For Fig. 11 (now renumbered), we have improved the visualisation by combining the experimental data and simulation data into a single figure to facilitate direct comparison.

[Major Point 2]: Vary multiple parameters. I appreciate authors doing a through sensitivity analysis to understand impact of individual parameters on colony growth details. However, the conclusions that there may be a range of values for a given parameter that give the needed colony shape is very dependent on whether other parameters stay at "default" values. I can see that boundary resistance (k_0) and diffusion coefficient (D) may be complementary in terms of their "compensation", so defining a "critical range" may not be fully valid when multiple parameters are varied. Please investigate.

[Author Response]: We have added a Parameter Hierarchy and Pattern Formation section addressing these points and expanded the section on the diffusion coefficient. We also outline our future plans to implement formal inference methods in the new Future Directions section of the Discussion, once computational performance improvements (e.g., GPU implementation) make more comprehensive parameter exploration tractable.

[Major Point 3]: Focus on D as the key parameter may be biased. I fully agree with the goal of finding parameter values that allow the model to work or not to work, so conclusion about importance of D is nice. However, in simulations D was varied orders of magnitude (Fig 20), and some resemblance to experimental data was for D varied about 10 fold (2x10^-11 to 3x10^-10); yet, k_0 needed to vary even less to see similar patterns (Fig 17: 1)). So, I think the authors should be more rigorous at comparing which parameter variability/values are important.

[Author Response]: This requires clarification. The critical distinction lies in *how* parameters affect system behavior rather than *how much* they vary. We revised the discussion, pointing that out in the new Parameter Hierarchy and Pattern Formation section.
Moreover, the identified range (2×10^{-11} to 10^{-10}m²/s) for D_s corresponds to small proteins and macromolecules (12–1,690 kDa), providing a testable experimental hypothesis: search for secreted signalling molecules in this molecular weight range. This represents a concrete, falsifiable prediction that can guide experimental investigation, whereas phenomenological boundary parameters lack direct molecular interpretation.

[Major Point 4]: Lack of alternative mechanisms and poor intuitive explanations of model behavior. I understand the need to have a working model but the current model lacks a lot of details about parasite's dynamics, e.g., limitation by resources/food or change in parasite's phenotype depending on whether they are located. Are there alternative mechanisms that would be able to explain the colony patterns)(e.g., suggested from previous work)? What elements of the model are key at generating the pattern? From the analyses it seems that all are important but can you "rank" them in some way? Also, I found the description of results of the sensitivity analyses a bit mechanical, lacking intuitive insights. Perhaps adding some explanations of why the model gives patterns at particular parameter values but not with others could be useful for some readers.

[Author Response]: We expanded the descriptions in the results section and added a Parameter Hierarchy and Pattern Formation, a Model Design Choices, and a Future Directions section in the discussion to address your points.

[Major Point 5]: What is "boundary"? I understand that to generate the finger-like pattern there is a need for some heterogeneity at the border of the colony. However, where it may be coming from could be debated, e.g., is that the substrate/agar or the parasites at the border change so they are different. Can you discuss this a bit more and propose how this could be tested experimentally?

[Author Response]: We discussed this in the new Model Design Choices section of the discussion.

[Minor Points]:
In Fig 1b, I assume that -9 means boundary but I am not sure. Please explain all numberings in caption.

[Author Response]: We updated the figure caption.

Should it be \Delta_{t_a} in eqn (7)?

[Author Response]: Yes that is correct. As t_a is the timestep of the agent update equations.

Please make sure figure captions have sufficient detail to explain the figure without the need to go to materials and methods unless that is required. E.g., caption of Fig 6 simply does not explain much.

[Author Response]: We updated many of the figure captions.

Add a/b/c... to each panel for each figure; many figures do not have that. And be consistent with these labelings, e.g., Fig 17 labels rows of panels as 1), 2), 3)... You can use Ai/Aii/etc and Bi/Bii/Biii etc if necessary.

[Author Response]: We changed the figure labellings to be consistent across figures.

Try to not have paragraphs consisting of a single or two sentences (e.g., lines 471-78, 500-, etc).

[Author Response]: We changed the formatting in some parts of the manuscript.

Having 21 figures in main text is impressive. It is not a major issue but perhaps there is a way to reduce the number of figures and move some to supplement.

[Author Response]: Sorry, the number of figures even increased by one due to the other reviewers' comments. But we were able to merge Figures 10 and 11 into a single figure.
