# Supplementary material for: An agent-based model of Trypanosoma brucei social motility to explore determinants of colony pattern formation
Source: PLoS Comput Biol. 2026 Aug 12;22(8):e1013698. doi: 10.1371/journal.pcbi.1013698 (PMC13489481; doi:10.1371/journal.pcbi.1013698)
Supplement: Supplementary file 4 [file pcbi.1013698.s004.pdf]

# Reviewer Report

- **Title:** An agent-based model of *Trypanosoma brucei* social motility to explore determinants of colony pattern formation (R1)
- **Authors:** Andreas Kuhn, Timothy Krüger, Markus Engstler, Sabine C. Fischer
- **Journal:** *PLOS Comput. Biol.*
- **Recommendation:** Minor revision.

## Summary

The four reports provided comprehensive feedback on the original submission, and I appreciate the authors' efforts to address my comments and those of the other referees. The updated GitHub home page and documentation looks good, and will provide a useful resource for the community and for extending the research. After the revision and the authors' responses, I am satisfied with the overall scientific content of the article. There remain some minor issues to address before finalising the publication, which I will outline below.

## Comments

1. Some of the paragraph breaks appear to be inserted arbitrarily, rather than to separate distinct ideas. In general, I think the manuscript would benefit from another round of careful proofreading and editing, to ensure that the findings are expressed as clearly as possible. My points below include some of these possible edits.
2. p. 1, Abstract: The treatment of the colony boundary is a unique feature of the model that warrants a separate sentence, to more clearly explain what is meant by "reflective and mutable boundaries".
3. p. 3, Lines 65–66: Walkable and non-walkable lattice sites should be briefly explained here.
4. p. 4, Line 94: Vectors should be formatted consistently (in bold). Currently, on line 94 the agent position vector $\boldsymbol{x}$ appears in bold typeface, whereas this quantity appears in regular font in Eqn. (2), and the position vector $x$ on line 97 (and near Eqn. (1), and on p. 7) is also in regular font. On p. 4, since $\boldsymbol{x}(t)$ and $\theta(t)$ correspond to individual agents, they should be indexed with a subscript. Indices should always be defined when they are introduced, with a statement like "for $i = 1, \ldots, N$, where $N$ is …"
5. p. 6, Eqn (2): Although the meaning of $s_{i,j}$ is not mentioned explicitly in the text, it would be more conventional for the first index to vary when calculating $s_{xx}$, and for the second index to vary when calculating $s_{yy}$.
6. p. 7, Eqn (4): $\alpha$ and $\eta_i$ should be defined immediately after they appear in this equation.

7. p. 13, Line 352: It is worth mentioning that the large number of agents in the simulation is the reason for the similarity between simulation runs. In this sense, the result is as expected, rather than remarkable.
8. p. 13, Line 380: "… simulated the system for 20 h *of simulation time* using …"
9. p. 17: I think the discussion of the differences between the simulation and experiments could still be expanded. I can see how the different timescales of growth and number of fingers between the model and experiment may lead to the results in Figure 11, but can you identify the difference(s) between the model and experiment might cause these trends to occur? I agree that more finely tuned parameter values could yield better agreement, but model misspecification is also possible. Both possibilities should be acknowledged in the text of this section. Also, the paragraph that appears after the figure (Lines 444–446) seems misplaced, and the sentence "Furthermore, we find good agreement between the simulations and experiment 2 for the morphological metrics" needs clarification. Is it correct that the most important comparison is between the final data point (of either the blue or red experiment) and the final point on the yellow curve (which has similar doubling time to the experiment)? If so, this should be made clear in the text, as the agreement is otherwise not particularly close.
10. p. 30, Lines 738–739: How feasible are experimental measurements of nutrient concentrations and spatially resolved cell proliferation rates? Are you aware of any experimental techniques that would enable this type of data?
11. p. 30, Line 772: Another possible technique for simulation-based inference is neural likelihood estimation (see for example [1]). This method can significantly reduce the number of simulations required compared to ABC, which may make SBI more viable for your problem.

## Bibliography

[1] G. Papamakarios, D. Sterratt, and I. Murray, "Sequential Neural Likelihood: Fast Likelihood-Free Inference with Autoregressive Flows," in *Proceedings of the Twenty-Second International Conference on Artificial Intelligence and Statistics*, Proceedings of Machine Learning Research, 2019, pp. 837–848. [Online]. Available: https://proceedings.mlr.press/v89/papamakarios19a.html
