# Supplementary material for: An agent-based model of Trypanosoma brucei social motility to explore determinants of colony pattern formation
Source: PLoS Comput Biol. 2026 Aug 12;22(8):e1013698. doi: 10.1371/journal.pcbi.1013698 (PMC13489481; doi:10.1371/journal.pcbi.1013698)
Supplement: Supplementary file 5 [file pcbi.1013698.s005.docx]

*PLOS Computational Biology*

Manuscript ID: PCOMPBIOL-D-25-02262R1

Dear Dr. Banga, and Members of the Editorial Board,

We would like to thank the editors and the reviewers for their continued engagement with our manuscript and for their constructive feedback. We are pleased that Reviewer #3 finds the overall scientific content satisfactory and recommends only minor revisions, and we appreciate the focused comments from Reviewer #4. Below, we provide a point-by-point response to all raised issues. Changes to the manuscript are highlighted in red in the tracked-changes version accompanying this letter.

**Journal Requirements**

***Requirement 1: Section order***

*Your manuscript's sections are not in the correct order. Please amend to the following order: Abstract, Introduction, Results, Discussion, and Methods.*

**We respectfully request to retain the current section order (Abstract, Introduction, Methods, Results, Discussion). The model architecture involves several novel design choices that go beyond established methods and constitute a scientific contribution in their own right. Given this complexity, we believe that presenting the Methods before the Results is essential for the reader to correctly follow and interpret the subsequent Results and Discussion sections. We therefore kindly ask the editors to consider granting an exception to the standard section order.**

***Requirement 2: Data deposition***

*In the online submission form, you indicated that your data will be submitted to a repository upon acceptance. We strongly recommend all authors deposit their data before acceptance, as the process can be lengthy and hold up publication timelines.*

**The data presented in this manuscript were derived from raw simulation output comprising agent positions, movement directions, and grid states across approximately 10⁶ agents and multiple timepoints, amounting to roughly 4 TB in total. Uploading this volume of raw data to a public repository is not feasible for practical reasons, including transfer time and environmental cost. Instead, we will ensure full reproducibility by depositing (i) all intermediate results required to reproduce every figure, (ii) the configuration files for every simulation run, and (iii) a copy of the model code. Together, these allow any reader to either reproduce the figures directly from the intermediate data or recreate the full raw data by re-running the model with the provided settings. The latter is made particularly tractable by the remarkably small variance between simulation runs (see Appendix). We have already started preparing the data which will be put into a Zenodo repository in the next weeks containing all of the above, including an archived copy of the model repository, the intermediate data, unprocessed output plots, and the simulation settings files.**

**Reviewer #3**

We thank Reviewer #3 for their thorough and careful reading of the revised manuscript. We address each point in turn.

***Comment R3.1 — General: paragraph breaks and proofreading***

*Some of the paragraph breaks appear to be inserted arbitrarily, rather than to separate distinct ideas. In general, I think the manuscript would benefit from another round of careful proofreading and editing, to ensure that the findings are expressed as clearly as possible.*

**We changed and removed some paragraphs/line breaks to make the manuscript more concise and clearer. And implemented all your provided edits of the manuscript.**

***Comment R3.2 — Abstract: 'reflective and mutable boundaries' (p. 1)***

*The treatment of the colony boundary is a unique feature of the model that warrants a separate sentence, to more clearly explain what is meant by 'reflective and mutable boundaries'.*

**We have added a separate sentence to the abstract to clarify this point: *"The colony boundary is modelled using a grid-based approach in which interactions with agents can locally weaken and expand it."*.**

***Comment R3.3 — Walkable and non-walkable lattice sites (p. 3, Lines 65–66)***

*Walkable and non-walkable lattice sites should be briefly explained here.*

**We have added a sentence at this location briefly explaining the division of the simulation space into walkable and non-walkable lattice sites, and their role in representing the colony boundary.**

***Comment R3.4 — Vector notation and agent indexing (p. 4, Line 94)***

*Vectors should be formatted consistently (in bold). Currently, on line 94 the agent position vector x appears in bold typeface, whereas this quantity appears in regular font in Eqn. (2), and the position vector x on line 97 (and near Eqn. (1), and on p. 7) is also in regular font. On p. 4, since x(t) and θ(t) correspond to individual agents, they should be indexed with a subscript. Indices should always be defined when they are introduced, with a statement like 'for i = 1, …, N, where N is …'*

**We changed that to stick more clearly to the common math notion.**

***Comment R3.5 — Index convention for s_ij in Eqn. (2) (p. 6)***

*Although the meaning of s_{i,j} is not mentioned explicitly in the text, it would be more conventional for the first index to vary when calculating s_{xx}, and for the second index to vary when calculating s_{yy}.*

**You are right, we changed to be more in line with common notation.**

***Comment R3.6 — Definition of α and η_i in Eqn. (4) (p. 7)***

*α and η_i should be defined immediately after they appear in this equation.*

**We changed that and also added the range of α later in the text.**

***Comment R3.7 — Similarity between simulation runs (p. 13, Line 352)***

*It is worth mentioning that the large number of agents in the simulation is the reason for the similarity between simulation runs. In this sense, the result is as expected, rather than remarkable.*

**We removed the word remarkable and expanded the appendix to explain this further.**

***Comment R3.8 — Wording suggestion (p. 13, Line 380)***

*"… simulated the system for 20 h of simulation time using …"*

**We changed that.**

***Comment R3.9 — Discussion of model–experiment discrepancies (p. 17)***

*I think the discussion of the differences between the simulation and experiments could still be expanded. I can see how the different timescales of growth and number of fingers between the model and experiment may lead to the results in Figure 11, but can you identify the difference(s) between the model and experiment that might cause these trends to occur? I agree that more finely tuned parameter values could yield better agreement, but model misspecification is also possible. Both possibilities should be acknowledged in the text of this section. Also, the paragraph that appears after the figure (Lines 444–446) seems misplaced, and the sentence 'Furthermore, we find good agreement between the simulations and experiment 2 for the morphological metrics' needs clarification. Is it correct that the most important comparison is between the final data point (of either the blue or red experiment) and the final point on the yellow curve (which has similar doubling time to the experiment)? If so, this should be made clear in the text, as the agreement is otherwise not particularly close.*

**We completely rewrote the section discussing the comparison between simulation and experiments, addressing your concerns about the not-so-good quantitative agreement between them.**

***Comment R3.10 — Feasibility of experimental measurements (p. 30, Lines 738–739)***

*How feasible are experimental measurements of nutrient concentrations and spatially resolved cell proliferation rates? Are you aware of any experimental techniques that would enable this type of data?*

**We thank the reviewer for this observation. The original formulation was indeed overly optimistic. Direct measurement of spatially resolved nutrient concentrations and cell proliferation rates within a colony is experimentally very challenging, as cells are motile and continuously redistribute within the colony, making spatially fixed measurements impractical. We have revised the text to reflect this more accurately. That said, an indirect experimental approach is feasible: by systematically varying the initial concentration of nutrients such as sugars, amino acids, or serum, and quantifying the resulting effects on colony expansion behavior, growth rate, and cell density, the influence of nutrient availability can be assessed without requiring spatially resolved measurements.**

***Comment R3.11 — Neural likelihood estimation as SBI alternative (p. 30, Line 772)***

*Another possible technique for simulation-based inference is neural likelihood estimation (see for example [Papamakarios et al., AISTATS 2019]). This method can significantly reduce the number of simulations required compared to ABC, which may make SBI more viable for your problem.*

**We added this to the discussion section, and also mentioned an example where neural likelihood estimation has been successfully applied to an agent-based model.**

**Reviewer #4**

We thank Reviewer #4 for their comments.

***Comment R4.1 — Claim of good agreement with Fig. 11 (Line 445)***

*Line 445 – a good agreement between the model predictions and the data. Looking at Fig 11, I do not see this, the model predicted lines are well off from the data. The authors need to provide some metric to state that the model matches the data or explain why the match is pretty poor.*

**We completely rewrote the section discussing the comparison between simulation and experiments, addressing your concerns about the not-so-good quantitative agreement between them.**

***Comment R4.2 — Missing citations for Fig. 10 and Fig. 11***

*Fig 11 should be cited (line 427–430). I did not see where you cited Fig 10 – that should be done between Fig 9 and Fig 11 then.*

**We rearranged Fig. 10 and Fig. 11 so that both are now cited when they appear.**

We hope that the above responses and the revised manuscript address all outstanding concerns. We look forward to the editors' further evaluation.

Yours sincerely,

Andreas Kuhn, Timothy Krüger, Markus Engstler, and Sabine C. Fischer
