# Supplementary material for: An agent-based model of Trypanosoma brucei social motility to explore determinants of colony pattern formation
Source: PLoS Comput Biol. 2026 Aug 12;22(8):e1013698. doi: 10.1371/journal.pcbi.1013698 (PMC13489481; doi:10.1371/journal.pcbi.1013698)
Supplement: Peer review history

# Peer review: Decision Letter 0

**DOI:** 10.1371/journal.pcbi.1013698.r001

**Authors:** Dominik Wodarz, Julio R. Banga

18 Dec 2025

PCOMPBIOL-D-25-02262

An agent-based model of Trypanosoma brucei social motility to explore determinants of colony pattern formation

PLOS Computational Biology

Dear Dr. Fischer,

Thank you for submitting your manuscript to PLOS Computational Biology. After careful consideration, we feel that it has merit but does not fully meet PLOS Computational Biology's publication criteria as it currently stands. Therefore, we invite you to submit a revised version of the manuscript that addresses the points raised during the review process.

Please submit your revised manuscript by Feb 17 2026 11:59PM. If you will need more time than this to complete your revisions, please reply to this message or contact the journal office at ploscompbiol@plos.org. When you're ready to submit your revision, log on to https://www.editorialmanager.com/pcompbiol/ and select the 'Submissions Needing Revision' folder to locate your manuscript file.

Please include the following items when submitting your revised manuscript:

* A rebuttal letter that responds to each point raised by the editor and reviewer(s). You should upload this letter as a separate file labeled 'Response to Reviewers'. This file does not need to include responses to formatting updates and technical items listed in the 'Journal Requirements' section below.

* A marked-up copy of your manuscript that highlights changes made to the original version. You should upload this as a separate file labeled 'Revised Manuscript with Track Changes'.

* An unmarked version of your revised paper without tracked changes. You should upload this as a separate file labeled 'Manuscript'.

If you would like to make changes to your financial disclosure, competing interests statement, or data availability statement, please make these updates within the submission form at the time of resubmission. Guidelines for resubmitting your figure files are available below the reviewer comments at the end of this letter

We look forward to receiving your revised manuscript.

Kind regards,

Julio R. Banga, Ph.D.

Academic Editor

PLOS Computational Biology

Dominik Wodarz

Section Editor

PLOS Computational Biology

**Journal Requirements:**

1) Please ensure that the CRediT author contributions listed for every co-author are completed accurately and in full.

At this stage, the following Authors/Authors require contributions: Andreas Kuhn, Timothy Krüger, Markus Engstler, and Sabine C. Fischer. Please ensure that the full contributions of each author are acknowledged in the "Add/Edit/Remove Authors" section of our submission form.

The list of CRediT author contributions may be found here: https://journals.plos.org/ploscompbiol/s/authorship#loc-author-contributions

2) We ask that a manuscript source file is provided at Revision. Please upload your manuscript file as a .doc, .docx, .rtf or .tex. If you are providing a .tex file, please upload it under the item type u2018LaTeX Source Fileu2019 and leave your .pdf version as the item type u2018Manuscriptu2019.

3) Please upload all main figures as separate Figure files in .tif or .eps format. For more information about how to convert and format your figure files please see our guidelines:

https://journals.plos.org/ploscompbiol/s/figures

4) We notice that your supplementary Figures are included in the manuscript file. Please remove them and upload them with the file type 'Supporting Information'. Please ensure that each Supporting Information file has a legend listed in the manuscript after the references list.

5) Please amend your detailed Financial Disclosure statement. This is published with the article. It must therefore be completed in full sentences and contain the exact wording you wish to be published.

1) State the initials, alongside each funding source, of each author to receive each grant. For example: "This work was supported by the National Institutes of Health (####### to AM; ###### to CJ) and the National Science Foundation (###### to AM)."

2) State what role the funders took in the study. If the funders had no role in your study, please state: "The funders had no role in study design, data collection and analysis, decision to publish, or preparation of the manuscript."

Note: Please ensure that the funders match between the Financial Disclosure field and the Funding Information tab in the online submission form. Both locations should list the same funders, grant numbers, and recipients in the same order.

6) Please ensure that your Data Availability Statement is updated in the online submission form.

Note: If the reviewer comments include a recommendation to cite specific previously published works, please review and evaluate these publications to determine whether they are relevant and should be cited. There is no requirement to cite these works unless the editor has indicated otherwise.

**Reviewers' comments:**

Reviewer's Responses to Questions

**Comments to the Authors:**

**Please note that one review is uploaded as an attachment.**

Reviewer #1: The manuscript “An agent-based model of Trypanosoma brucei social motility to explore determinants of colony pattern formation” by Kuhn et al. focuses on developing and simulating a model capable of studying the underlying mechanisms got single-cell motility within a large colony. It is noted that choosing an agent-based model highlights their ability to isolate single-cell behavior among 10^5-10^6 simulated cells. Using the model the authors test a range of potential mechanisms that lead to differing colony pattern formations , specifically fingering patterns, on a two-dimensional surface. They identified the diffusion strength as well as the interplay between orientation noise and bacterial turning rate as key parameters in the macroscopic pattern formation. Not only do they identify key parameters but also give specific ranges to see the interesting large-scale behavior that could be tested experimentally. One particular element of note that I found most intriguing and particularly clever was how they handled colony boundary interactions using parameters for grid strength and grid recovery rate to make these interface interactions more realistic.

This is a great example where mathematical models can provide insight where experiment can be challenging. While the authors do not have their own experimental data to verify the model predictions, they verify against previous studies and make a convincing argument for research groups with those experimental capabilities to investigate the mechanisms uncovered here. Overall, this manuscript was very well-written, and it was a pleasure to read. My suggestions below are only minor tweaks, and I think this paper would fit well within the scope of the Journal and this work is accessible as written to readers from graduate students to senior scientists.

Remarks/Questions:

1. While reading this paper I thought if a recent work I read Xue, Budrene, and Othmer that looks at radial fingering patterns in Proteus mirabilis. It may be worth citing or looking at for comparative purposes.

2. In all the images it seems the boundary fingers were more or less the same length throughout time. Is there any scenario where this wouldn’t happen such as inhomogeneous colony boundary expansion conditions? That might be an interesting simulation to look at with the model in the future.

3. You mention that the model simulations grow faster outward than experimental data:

a. Could this be fixed by adjusting the grid strength on the boundary interactions? I assume it is more complicated than that given that you probably tried that.

b. Would incorporating some kind of excluded-volume constraint on the individual bacteria affect the expansion rate or patterning? It would make it computationally more difficult, but collisions or jamming could change the patterns observed.

4. Do you believe this is generalizable to other microorganisms or possibly it depends on their movement mechanism, which may be unique to the strain studied?

Minor Remarks:

1. Mathematical equations should be treated as part of a sentence. So, if a sentence ends with an equation, that equation should end with a period. If it is in the middle of a sentence often a comma can be found at the end of the equation.

2. In Line 132, I would suggest a central dot. In equation editor you can use \cdot, so in equation editor it could be \delta(\cdot).

3. Line 605, fix the first quotation mark.

Reviewer #2: This paper models the growth of Trypanosoma brucei using an agent-based model on a lattice. Different grids are used for the cells and a chemical field. Due to disagreement between the model and experiments, it is hypothesised that alternative chemical signals should be investigated. The paper generally reads well and justifies choices in the model formulation.

The main question I have is around the importance of features in the model. By varying parameters, the authors find that the chemical diffusion has a crucial role in the colony shape. Other models have used a similar approach but without the complex boundary mechanism introduced here and have produced similar fingering patterns. Could the authors comment on whether the patterns produced are due to the boundary mechanism or could be the result of diffusion limited growth. I believe this must be addressed satisfactorily for the paper to be published.

I would also like the authors to comment on the approach for parameter exploration. Was consideration given to a more formal approach, such as approximate Bayesian computation? This would give a more systematic comparison.

Other items to address:

The variable t very likely represents time but should be clearly defined.

Does the gradient grid within one ABM cell have the same concentration? This was not clear and Figure 1 appears to suggest this is the case, which would seemingly remove the purpose of the finer grid.

Are the cell angles restricted in any way? How is this converted to a direction on the lattice?

Is the abbreviation “SoMo” used later? Perhaps this does not need to be introduced. I would recommend being consistent with the capitalisation of “Social Motility”.

Figure 3: It would be useful to include a statement in the caption to explicitly state what the green cells represent.

How are Rt and Rc chosen? While these are in category c, was there any motivation or are these just selected to make the mode work?

Figure 4: If the shape used in the model is not a circle, I would update the figure to illustrate this.

When specifying CPU performance, please add what a 2025 CPU represents in terms of computing power.

Table 1: Stray *, which I believe is standing in for a multiplication sign. Consider expressing the absorption rate and decay rate in scientific notation too.

It’s noted that the variation between simulations was “remarkably small”. Can you quantify this? It would help the reader judge the impact of the choice not to repeat simulations. Could standard deviations be reported for one case?

Can you explain why the faster growing colony produces more fingers, and more uniform fingers?

The formulation seems to switch between measuring angles in degrees and radians. One of these should be selected and used consistently.

Figure 12: Would the colony with noise 0.75 later form fingers if simulated for longer?

It’s claimed that a visual analysis indicates the best experimental match when eta is 0.2 and alpha is 0.02-0.1. Do the metrics computed also support this claim?

Reviewer #3: Please see the attached PDF.

Reviewer #4: Positive feedback. It is well written paper with a lot of details given to explain how the model is formulated and analyzed, thank you. I liked all visualizations; however, some figures were quite small with very difficult to see labelings on individual panels. Overall, great job.

Major comments

1. Show experimental data. The key element of simulations is to reproduce experimental patterns. However, for me to know what the pattern looks like I had to go to the Kuhn et al. 2024 J Roy Soc Int to look it up. I think showing examples of the patterns formed, e.g., next to photo of the parasite (Fig S1), would be very useful. Fig 11 does show experimental data (well, some metrics extracted from the images), but they are hard to see or compare to simulations. Connecting points with lines and making sure y/x axes scales are the same between experiment and simulations could be useful. Also, for experiments, you need to provide much more information on how experiments/measurements were done than Fig 11 caption suggests. Also include these descriptions in Materials and methods.

2. Vary multiple parameters. I appreciate authors doing a through sensitivity analysis to understand impact of individual parameters on colony growth details. However, the conclusions that there may be a range of values for a given parameter that give the needed colony shape is very dependent on whether other parameters stay at "default" values. I can see that boundary resistance (k_0) and diffusion coefficient (D) may be complementary in terms of their "compensation", so defining a "critical range" may not be fully valid when multiple parameters are varied. Please investigate.

3. Focus on D as the key parameter may be biased. I fully agree with the goal of finding parameter values that allow the model to work or not to work, so conclusion about importance of D is nice. However, in simulations D was varied orders of magnitude (Fig 20), and some resemblance to experimental data was for D varied about 10 fold (2x10^-11 to 3x10^-10); yet, k_0 needed to vary even less to see similar patterns (Fig 17: 1)). So, I think the authors should be more rigorous at comparing which parameter variability/values are important.

4. Lack of alternative mechanisms and poor intuitive explanations of model behavior. I understand the need to have a working model but the current model lacks a lot of details about parasite's dynamics, e.g., limitation by resources/food or change in parasite's phenotype depending on whether they are located. Are there alternative mechanisms that would be able to explain the colony patterns )(e.g., suggested from previous work)? What elements of the model are key at generating the pattern? From the analyses it seems that all are important but can you "rank" them in some way? Also, I found the description of results of the sensitivity analyses a bit mechanical, lacking intuitive insights. Perhaps adding some explanations of why the model gives patterns at particular parameter values but not with others could be useful for some readers.

5. What is "boundary"? I understand that to generate the finger-like pattern there is a need for some heterogeneity at the border of the colony. However, where it may be coming from could be debated, e.g., is that the substrate/agar or the parasites at the border change so they are different. Can you discuss this a bit more and propose how this could be tested experimentally?

Minor comments.

In Fig 1b, I assume that -9 means boundary but I am not sure. Please explain all numberings in caption.

Should it be \Delta_{t_a} in eqn (7)?

Please make sure figure captions have sufficient detail to explain the figure without the need to go to materials and methods unless that is required. E.g., caption of Fig 6 simply does not explain much.

Add a/b/c... to each panel for each figure; many figures do not have that. And be consistent with these labelings, e.g., Fig 17 labels rows of panels as 1), 2), 3)... You can use Ai/Aii/etc and Bi/Bii/Biii etc if necessary.

Try to not have paragraphs consisting of a single or two sentences (e.g., lines 471-78, 500-, etc).

Having 21 figures in main text is impressive. It is not a major issue but perhaps there is a way to reduce the number of figures and move some to supplement.

**********

**Have the authors made all data and (if applicable) computational code underlying the findings in their manuscript fully available?**

The PLOS Data policy requires authors to make all data and code underlying the findings described in their manuscript fully available without restriction, with rare exception (please refer to the Data Availability Statement in the manuscript PDF file). The data and code should be provided as part of the manuscript or its supporting information, or deposited to a public repository. For example, in addition to summary statistics, the data points behind means, medians and variance measures should be available. If there are restrictions on publicly sharing data or code —e.g. participant privacy or use of data from a third party—those must be specified.

Reviewer #1: Yes

Reviewer #2: **No:**  While the code is provided via GitHub, the data generated by the model does not appear to be included there or elsewhere

Reviewer #3: Yes

Reviewer #4: Yes

**********

PLOS authors have the option to publish the peer review history of their article (what does this mean?). If published, this will include your full peer review and any attached files.

If you choose “no”, your identity will remain anonymous but your review may still be made public.

**Do you want your identity to be public for this peer review?**  For information about this choice, including consent withdrawal, please see our Privacy Policy.

Reviewer #1: **Yes:**  Shawn D. Ryan

Reviewer #2: No

Reviewer #3: No

Reviewer #4: No

[NOTE: If reviewer comments were submitted as an attachment file, they will be attached to this email and accessible via the submission site. Please log into your account, locate the manuscript record, and check for the action link "View Attachments". If this link does not appear, there are no attachment files.]

**Figure resubmission:**

While revising your submission, we strongly recommend that you use PLOS’s NAAS tool (https://ngplosjournals.pagemajik.ai/artanalysis) to test your figure files. NAAS can convert your figure files to the TIFF file type and meet basic requirements (such as print size, resolution), or provide you with a report on issues that do not meet our requirements and that NAAS cannot fix.

After uploading your figures to PLOS’s NAAS tool - https://ngplosjournals.pagemajik.ai/artanalysis, NAAS will process the files provided and display the results in the "Uploaded Files" section of the page as the processing is complete. If the uploaded figures meet our requirements (or NAAS is able to fix the files to meet our requirements), the figure will be marked as "fixed" above. If NAAS is unable to fix the files, a red "failed" label will appear above. When NAAS has confirmed that the figure files meet our requirements, please download the file via the download option, and include these NAAS processed figure files when submitting your revised manuscript.

**Reproducibility:**

To enhance the reproducibility of your results, we recommend that authors of applicable studies deposit laboratory protocols in protocols.io, where a protocol can be assigned its own identifier (DOI) such that it can be cited independently in the future. Additionally, PLOS ONE offers an option to publish peer-reviewed clinical study protocols. Read more information on sharing protocols at https://plos.org/protocols?utm_medium=editorial-email&utm_source=authorletters&utm_campaign=protocols

Attachment Submitted filename: Review_Kuhn2025.pdf

# Author comment: Author response to Decision Letter 1

**DOI:** 10.1371/journal.pcbi.1013698.r002

26 Mar 2026

Attachment Submitted filename: letter_to_editor.pdf

# Peer review: Decision Letter 1

**DOI:** 10.1371/journal.pcbi.1013698.r003

**Authors:** Amber M Smith, Julio R. Banga

17 May 2026

PCOMPBIOL-D-25-02262R1

An agent-based model of Trypanosoma brucei social motility to explore determinants of colony pattern formation

PLOS Computational Biology

Dear Dr. Fischer,

Thank you for submitting your manuscript to PLOS Computational Biology. After careful consideration, we feel that it has merit but does not fully meet PLOS Computational Biology's publication criteria as it currently stands. Therefore, we invite you to submit a revised version of the manuscript that addresses the points raised during the review process.

Please submit your revised manuscript by Jul 17 2026 11:59PM. If you will need more time than this to complete your revisions, please reply to this message or contact the journal office at ploscompbiol@plos.org. When you're ready to submit your revision, log on to https://www.editorialmanager.com/pcompbiol/ and select the 'Submissions Needing Revision' folder to locate your manuscript file.

Please include the following items when submitting your revised manuscript:

* A letter that responds to each point raised by the editor and reviewer(s). You should upload this letter as a separate file labeled 'Response to Reviewers'. This file does not need to include responses to formatting updates and technical items listed in the 'Journal Requirements' section below.

* A marked-up copy of your manuscript that highlights changes made to the original version. You should upload this as a separate file labeled 'Revised Manuscript with Track Changes'.

* An unmarked version of your revised paper without tracked changes. You should upload this as a separate file labeled 'Manuscript'.

If you would like to make changes to your financial disclosure, competing interests statement, or data availability statement, please make these updates within the submission form at the time of resubmission. Guidelines for resubmitting your figure files are available below the reviewer comments at the end of this letter.

We look forward to receiving your revised manuscript.

Kind regards,

Julio R. Banga, Ph.D.

Academic Editor

PLOS Computational Biology

Dominik Wodarz

Section Editor

PLOS Computational Biology

**Journal Requirements:**

If the reviewer comments include a recommendation to cite specific previously published works, please review and evaluate these publications to determine whether they are relevant and should be cited. There is no requirement to cite these works unless the editor has indicated otherwise.

1) Your manuscript's sections are not in the correct order.  Please amend to the following order: Abstract, Introduction, Results, Discussion, and Methods

2) In the online submission form, you indicated that your data will be submitted to a repository upon acceptance. We strongly recommend all authors deposit their data before acceptance, as the process can be lengthy and hold up publication timelines. Please note that, though access restrictions are acceptable now, your entire minimal dataset will need to be made freely accessible if your manuscript is accepted for publication. This policy applies to all data except where public deposition would breach compliance with the protocol approved by your research ethics board. If you are unable to adhere to our open data policy, please kindly revise your statement to explain your reasoning and we will seek the editor's input on an exemption.

**Reviewers' comments:**

Reviewer's Responses to Questions

**Comments to the Authors:**

**Please note here if the review is uploaded as an attachment.**

Reviewer #3: Please see the attached PDF.

Reviewer #4: Minor comments

Line 445 - a good agreement between the model predictions and the data. Looking at Fig 11, I do not see this, the model predicted lines are well off from the data. The authors need to provide some metric to state that the model matches the data or explain why the match is pretty poor.

Fig 11 should be cited (line 427-430). I did not see where you cited Fig 10 - that should be done between Fig 9 and Fig 11 then.

**********

**Have the authors made all data and (if applicable) computational code underlying the findings in their manuscript fully available?**

The PLOS Data policy requires authors to make all data and code underlying the findings described in their manuscript fully available without restriction, with rare exception (please refer to the Data Availability Statement in the manuscript PDF file). The data and code should be provided as part of the manuscript or its supporting information, or deposited to a public repository. For example, in addition to summary statistics, the data points behind means, medians and variance measures should be available. If there are restrictions on publicly sharing data or code —e.g. participant privacy or use of data from a third party—those must be specified.

Reviewer #3: Yes

Reviewer #4: Yes

**********

PLOS authors have the option to publish the peer review history of their article (what does this mean?). If published, this will include your full peer review and any attached files.

If you choose “no”, your identity will remain anonymous but your review may still be made public.

**Do you want your identity to be public for this peer review?**  For information about this choice, including consent withdrawal, please see our Privacy Policy.

Reviewer #3: No

Reviewer #4: No

[NOTE: If reviewer comments were submitted as an attachment file, they will be attached to this email and accessible via the submission site. Please log into your account, locate the manuscript record, and check for the action link "View Attachments". If this link does not appear, there are no attachment files.]

**Figure resubmission:**

-->While revising your submission, we strongly recommend that you use PLOS’s NAAS tool (https://ngplosjournals.pagemajik.ai/artanalysis) to test your figure files. NAAS can convert your figure files to the TIFF file type and meet basic requirements (such as print size, resolution), or provide you with a report on issues that do not meet our requirements and that NAAS cannot fix.

After uploading your figures to PLOS’s NAAS tool - https://ngplosjournals.pagemajik.ai/artanalysis, NAAS will process the files provided and display the results in the "Uploaded Files" section of the page as the processing is complete. If the uploaded figures meet our requirements (or NAAS is able to fix the files to meet our requirements), the figure will be marked as "fixed" above. If NAAS is unable to fix the files, a red "failed" label will appear above. When NAAS has confirmed that the figure files meet our requirements, please download the file via the download option, and include these NAAS processed figure files when submitting your revised manuscript.

**Reproducibility:**

To enhance the reproducibility of your results, we recommend that authors of applicable studies deposit laboratory protocols in protocols.io, where a protocol can be assigned its own identifier (DOI) such that it can be cited independently in the future. Additionally, PLOS ONE offers an option to publish peer-reviewed clinical study protocols. Read more information on sharing protocols at https://plos.org/protocols?utm_medium=editorial-email&utm_source=authorletters&utm_campaign=protocols

Attachment Submitted filename: Review_Kuhn2025_R1.pdf

# Author comment: Author response to Decision Letter 2

**DOI:** 10.1371/journal.pcbi.1013698.r004

20 Jul 2026

Attachment Submitted filename: Response_to_Reviewers.docx

# Editor report: Decision Letter 2

**DOI:** 10.1371/journal.pcbi.1013698.r005

**Authors:** Amber M Smith, Julio R. Banga

22 Jul 2026

Dear Mr Kuhn,

We are pleased to inform you that your manuscript 'An agent-based model of Trypanosoma brucei social motility to explore determinants of colony pattern formation' has been provisionally accepted for publication in PLOS Computational Biology.

Before your manuscript can be formally accepted you will need to complete some formatting changes, which you will receive in a follow up email. A member of our team will be in touch with a set of requests.

Please note that your manuscript will not be scheduled for publication until you have made the required changes, so a swift response is appreciated.

IMPORTANT: The editorial review process is now complete. PLOS will only permit corrections to spelling, formatting or significant scientific errors from this point onwards. Requests for major changes, or any which affect the scientific understanding of your work, will cause delays to the publication date of your manuscript.

Should you, your institution's press office or the journal office choose to press release your paper, you will automatically be opted out of early publication. We ask that you notify us now if you or your institution is planning to press release the article. All press must be co-ordinated with PLOS.

Thank you again for supporting Open Access publishing; we are looking forward to publishing your work in PLOS Computational Biology.

Best regards,

Julio R. Banga, Ph.D.

Academic Editor

PLOS Computational Biology

Amber Smith

Section Editor

PLOS Computational Biology

***********************************************************

I suggest adding the Zenodo link at this stage.

# Editor report: Acceptance letter

**DOI:** 10.1371/journal.pcbi.1013698.r006

**Authors:** Amber M Smith, Julio R. Banga

PCOMPBIOL-D-25-02262R2

An agent-based model of Trypanosoma brucei social motility to explore determinants of colony pattern formation

Dear Dr Kuhn,

I am pleased to inform you that your manuscript has been formally accepted for publication in PLOS Computational Biology. Your manuscript is now with our production department and you will be notified of the publication date in due course.

The corresponding author will soon be receiving a typeset proof for review, to ensure errors have not been introduced during production. Please review the PDF proof of your manuscript carefully, as this is the last chance to correct any errors. Please note that major changes, or those which affect the scientific understanding of the work, will likely cause delays to the publication date of your manuscript.

Soon after your final files are uploaded, unless you have opted out, the early version of your manuscript will be published online. The date of the early version will be your article's publication date. The final article will be published to the same URL, and all versions of the paper will be accessible to readers.

For Research, Software, and Methods articles, you will receive an invoice from PLOS for your publication fee after your manuscript has reached the completed accept phase. If you receive an email requesting payment before acceptance or for any other service, this may be a phishing scheme. Learn how to identify phishing emails and protect your accounts at https://explore.plos.org/phishing.

Thank you again for supporting PLOS Computational Biology and open-access publishing. We are looking forward to publishing your work!

With kind regards,

Sharmila Kamatchi

PLOS Computational Biology | Carlyle House, Carlyle Road, Cambridge CB4 3DN | United Kingdom ploscompbiol@plos.org | Phone +44 (0) 1223-442824 | ploscompbiol.org | @PLOSCompBiol
